# Bi-directional regulation between NAD/NAMPT and IFN-γ/PD-L1 axes via BRD4/IRF1 and mitochondrial respiration in metastatic cutaneous melanoma

**DOI:** 10.1186/s13046-026-03734-2

**Published:** 2026-05-14

**Authors:** Irene Fiorilla, Beatrice Ghezzi, Alessia Ponzano, Enrico Moiso, Federica Riccardo, Nicoletta Tommasi, Lidia Avalle, Giovanna Carrà, Filippo Ugolini, Edoardo Calussi, Alberto Maria Todesco, Sabrina Digiovanni, Filippo Casone, Giulia Rizza, Luca Ponzone, Anna Szumera-Ciećkiewicz, Maria Cavaletto, Paolo Ettore Porporato, Laura Conti, Chiara Riganti, Daniela Massi, Enzo Calautti, Valentina Audrito

**Affiliations:** 1 Department of Science and Technological Innovation, University of Eastern Piedmont, Alessandria, Italy; 2 Department of Epidemiology and Biostatistics, Memorial Sloan Kettering Cancer Center, New York, NY USA; 3 Department of Molecular Biotechnology and Health Sciences, Molecular Biotechnology Center “Guido Tarone”, University of Turin, Torino, Italy; 4 San Luigi Gonzaga Hospital, Regione Gonzole, Orbassano, Italy; 5 Department of Clinical and Biological Sciences, University of Turin, Torino, Italy; 6 Histopathology and Molecular Diagnostics, Careggi University Hospital, Florence, Italy; 7 Department of Oncology, Molecular Biotechnology Center “Guido Tarone”, University of Turin, Torino, Italy; 8 Biobank, Maria Sklodowska-Curie National Research Institute of Oncology, Warsaw, Poland; 9 Department of Pathology and Laboratory Medicine, Maria Sklodowska- Curie National Research Institute of Oncology, Warsaw, Poland; 10 Department of Sustainable Development and Ecological Transition, University of Eastern Piedmont, Vercelli, Italy; 11 Department of Health Sciences, Section of Pathology, University of Florence, Florence, Italy; 12 Department of Molecular Pathobiology, New York University - College of Dentistry, New York, USA; 13 Research Laboratories, Research and Innovation Department (DAIRI), Azienda Ospedaliera Universitaria AOU “SS. Antonio e Biagio e Cesare Arrigo”, Alessandria, Italy

**Keywords:** NAD, NAMPT, Interferon, BET protein, PD-L1, Metastatic melanoma, Mitochondrial respiration, Inflammation, Tumor microenvironment

## Abstract

**Background:**

Metastatic cutaneous melanoma (MCM) is primarily treated with BRAF/MEK inhibitors and immune checkpoint inhibitors (ICIs), but the long-term efficacy of these therapies is often limited by acquired resistance. Nicotinamide phosphoribosyltransferase (NAMPT), the rate-limiting enzyme in NAD biosynthesis, is frequently upregulated in MCM, supporting metabolic rewiring and targeted therapy resistance.

Interferon-γ (IFN-γ) signaling plays a central role in melanoma biology, exerting both antitumor and immunoregulatory effects, linked with the onset of therapeutic resistance. Emerging evidence suggests that metabolic pathways may critically modulate IFN-γ responses; however, the functional interplay between NAD/NAMPT metabolism and IFN-γ signaling in melanoma cells remains poorly defined.

**Methods:**

We integrated transcriptomic, bioinformatic, biochemical, and functional approaches in human and murine melanoma cell lines, together with analyses of TCGA datasets and a tissue microarray (TMA) cohort. Mechanistic studies included pharmacological and genetic perturbation of Bromodomain and Extra-Terminal motif (BET) epigenetic factor BRD4, Interferon Regulatory Factor 1 (IRF1), and NAMPT, chromatin immunoprecipitation (ChIP) assays, and metabolic analyses. Tumor-T cell co-culture systems were used to assess the impact of melanoma-cell NAMPT modulation on T-cell behavior.

**Results:**

IFN-γ induced NAMPT expression through a BRD4/IRF1-dependent transcriptional program. In turn, NAMPT activity was required to sustain IFN-γ signaling, as its inhibition impaired STAT1 activation and downstream transcriptional responses. Mechanistically, NAMPT-dependent NAD metabolism supported mitochondrial complex I activity and oxidative metabolism and was required for efficient BRD4 recruitment to IFN-responsive promoters, including CD274/PD-L1 and NAMPT itself. Across melanoma datasets and patient samples, NAMPT expression correlated with IFN-γ–responsive genes, including PD-L1. Functionally, modulation of NAMPT in melanoma cells influenced T-cell cytotoxicity and migration in co-culture systems.

**Conclusions:**

Overall, these findings identify NAMPT as a key metabolic component of the IFN-γ response network in melanoma cells, establishing a feed-forward regulatory circuit linking cytokine signaling, chromatin regulation, and mitochondrial metabolism. This work provides a framework to investigate how metabolic control of IFN-γ signaling shapes tumor-immune interactions.

**Supplementary Information:**

The online version contains supplementary material available at 10.1186/s13046-026-03734-2.

## Introduction

Interferons are critical immunomodulatory cytokines involved in orchestrating antimicrobial and antitumor immune responses. Among them, interferon-gamma (IFN-γ), predominantly secreted by immune effector cells, exerts multifaceted effects on tumor cells by modulating various intracellular signaling cascades including the canonical Janus kinase/signal transducer and activator of transcription (JAK/STAT) pathway [1]. In metastatic cutaneous melanoma (MCM), IFN-γ can mediate antitumor functions through induction of cell cycle arrest and cytotoxicity, primarily via activation of the JAK/STAT/IRF1 axis. However, due to its pleiotropic nature, IFN signaling can also contribute to the development of resistance mechanisms that ultimately undermine the durability of ICI-based therapies [1–3]. In fact, sustained low levels of IFN-γ promote the expression of cytotoxic T-lymphocyte associated protein-4 (CTLA-4), programmed death-1 ligand 1 (PD-L1) and 2 (PD-L2), and indoleamine 2,3-dioxygenase 1 (IDO1), all involved in immunosuppressive effects. Importantly, IFN-γ also plays a pivotal role in shaping the therapeutic response to immune checkpoint inhibitors (ICIs), which have become a central approach in the MCM clinical management [1–3]. Moreover, it has been proposed that resistance to ICIs shares common effector mechanisms that also play a role in the onset of therapeutic resistance to BRAF inhibitors (BRAFi) [4–8].

It has been reported that IFN-γ increases the expression of nicotinamide phosphoribosyltransferase (NAMPT), the key-limiting enzyme involved in nicotinamide adenine dinucleotide (NAD) biosynthesis, which can also act as a soluble factor modulating tumor-immune crosstalk in the tumor microenvironment (TME) [9–12]. We and others have previously demonstrated that increase in NAMPT expression is a key event in metabolic rewiring and targeted therapy (TT), and in particular in BRAFi resistance in MCM [13–17]. NAD metabolism is required in general to fuel energetic cellular needs and detoxification systems, especially during cellular stress conditions [18, 19]. This is also evident in immune cells like monocytes/macrophages upon viral infection, where NAD is rapidly depleted and regenerated from nicotinamide (NAM) or nicotinamide riboside (NR) precursors via interferon signaling activation of NAMPT or NR-kinase (NMRK) enzymatic activities, respectively [20]. Therefore, NAD emerged as key factor in regulation of immune cell function, with implications in immune homeostasis, inflammation, and disease [21, 22]. In immune cells (T cells and monocytes/macrophages) and antigen-presenting cells IFN-γ pathway activation is required for their cellular metabolic adaptation and vice versa [23, 24]. IFN-γ exposure in monocytes boosts their oxidative metabolism promoting the respiratory burst with increased mitochondrial respiration/oxygen consumption rate (OCR), and this phenotype is dependent on NAMPT-mediated NAD regeneration [25]. It has been also demonstrated that IFN-STAT1-inducible *Nampt* shapes the metabolic program and functions of tumor associated macrophages [26].

Importantly, the interaction between NAD/NAMPT axis and the IFN-γ-pathway is emerging also in oncological contexts. In fact, recent studies showed that in both liver and bladder carcinomas, NAMPT/NAD impinges on the IFN-γ-STAT1 axis, via a NAD-dependent epigenetic mechanism, which potentiates IFN-γ-induced PD-L1 expression and immune evasion, but at the same time it also increases the sensitivity of tumors to immunotherapy [27, 28]. Moreover, in pancreatic ductal adenocarcinoma (PDAC), IFN signaling promotes the consumption of NAD(H) through upregulation of poly (ADP-ribose) polymerases (PARPs) including PARP9, PARP10, and PARP14, thereby sensitizing tumor cells to NAMPT-targeting strategies [29]. NAMPT-induction via IFN-γ was also described in melanoma and was associated with the metabolic reprogramming of melanoma cells and increased growth of tumors in vivo [30]. However, to date the only known molecular mechanism that links IFN-γ signaling and NAMPT is STAT1-dependent transcriptional regulation [26], but the epigenetic determinants at the basis of NAMPT regulations and the overall implications of the crosstalk between the NAD/NAMPT and IFN-γ/PD-L1 axes in melanoma are still poorly understood.

In this study, we report a robust induction of NAMPT mRNA and protein expression in response to IFN-γ in human and murine BRAF-mutated (V600E) and BRAF wild-type (WT) MCM cell lines, which recapitulates the adaptive phenotype we previously described in BRAFi-resistant melanomas cells and tumors [14, 15, 31]. Mechanistically, we reveal that the molecular mechanisms of NAMPT regulation through IFN-γ involves Bromodomain and Extra-Terminal motif (BET) epigenetic factor BRD4 and Interferon Regulatory Factor 1 (IRF1). Moreover, our work uncovers a positive feed-forward loop whereby NAMPT is induced by IFN-γ, and the increase in NAMPT-dependent NAD metabolism is in turn required to further sustain IFN-γ signaling via activation of mitochondrial complex I/oxidative metabolism. Functionally, modulation of NAMPT in melanoma cells influenced T-cell cytotoxicity and migration in co-culture systems.

From a translational perspective, our findings highlight NAMPT as a metabolic regulator of IFN-γ responsiveness in melanoma cells, particularly regulating PD-L1 expression, establishing a feed-forward regulatory circuit linking cytokine signaling, chromatin regulation, and mitochondrial metabolism. This work provides a conceptual framework to investigate how metabolic control of IFN-γ signaling shapes tumor-immune interactions.

## Materials and methods

### Cell Culture

501MEL, A375, M14, SK-MEL-28 (BRAFV600E-mutated human melanoma cell lines), MEWO (BRAF wild-type WT human melanoma cell line, but characterized by NF1 loss of functions mutations), D4M 3A3 (BRAFV600E murine melanoma cell line) and B16-F10 (BRAF WT murine melanoma cell line) were previously obtained from the American Type Culture Collection (ATCC) or kindly provided as gift by collaborators [14, 31] and cultured in RPMI-1640 (Corning Inc., Cat #10-040-CV, Corning, NY, USA) or DMEM (Corning Inc., 10-013-CV) supplemented with 10% (v/v) fetal bovine serum (Capricorn Scientific, FBS-11 A, #CP21-4466, Ebsdorfergrund, Germany), 1% MEM Non-Essential Amino Acids Solution (Gibco, Thermo Fisher Scientific, #11140035, Segrate, MI, Italy), 1% MEM Vitamin Solution (Gibco, #11120037), 10 mM HEPES Buffer Solution (Capricorn Scientific, HEP-B, #CP24-7334), Sodium pyruvate solution 100 mM (Capricorn Scientific, NPY-B, #CP23-6474) and 1% Gentamycin (Gibco, #15750-037). Cells were maintained in these culture conditions for all experiments except where specifically indicated. All cell lines were routinely tested for Mycoplasma contamination.

### Signaling experiments

For one set of experiments, cell lines were treated for 24 h with human or murine IFN-γ 100 ng/ml (human #300-02, murine #315-05 Peprotech, Thermo Fisher Scientific), JQ1 5 µM (HY-13030) and AZD5153 100 nM (HY-100653) both from MedChemExpress (Sollentuna, Sweden), or their combination. For the second set of experiments cells were treated for 24 h with IFN-γ 100 ng/ml alone or in combination with NAMPT inhibitor, FK866 25 nM (FK, S2799, Selleckchem, Houston, Texas). For the eNAMPT quantification cells were treated for 48 h with IFN-γ 200 ng/ml.

### Quantitative real-time PCR (qRT-PCR) analysis and primers

RNA was isolated from cells using NucleoSpin RNA (Macherey-Nagel, #FC140955N, Düren, Germany) according to the manufacturer’s instructions and quantified using Qubit (Qubit RNA BR Assay Kits, #Q10210, Thermo Fisher Scientific, Milan, Italy) and a Spectrophotometer Varioskan Lux (Thermo Scientific, #VLBL00D0).

RNA was converted to cDNA using the High-Capacity cDNA Reverse Transcription kit (Thermo Fisher Scientific, 4368814). Commercially available primers are listed in Supplementary Materials and Methods. qRT-PCR was performed using the CFX384 Real-time System (Bio-Rad Laboratories Srl, Segrate MI, Italy). Relative mRNA expression of the indicated genes was measured by RT-qPCR and normalized to the selected housekeeping genes using the ΔCt method [14]. Data are expressed as relative expression (target/housekeeping) values.

### RNA sequencing

RNA sequencing via Illumina platforms, based on the mechanism of SBS (sequencing by synthesis), was performed exploiting Novogene mRNA sequencing service (Novogene Europe, Cambridge, UK) as described in Supplementary Material and Methods. RNAs were extracted, using the same method described in RT-PCR section, from BRAFV600E 501MEL cell line in the following conditions: untreated (UN), IFN-γ (100ng/ml) alone or in combination with FK866 (25 nM) from 24 h (3 biological replicates/condition).

Data were deposited to Gene Expression Omnibus (GEO) repository with the accession number GSE310354.

### Western blot analysis

Cells were lysed as previously described [14, 31] and protein concentration was measured using the Bradford assay (Bio-Rad, #5000006). Lysates were diluted in Laemmli buffer (Bio-Rad, #161–0747) and boiled for 5 min at 95 °C. Equal amounts of proteins were loaded on 4–20% Mini- PROTEAN^®^ TGX™ Precast Protein Gels (Bio-Rad, #4568084) and transferred to Nitrocellulose Transfer Membrane (Trans-Blot Turbo RTA Midi 0.2 μm Nitrocellulose Transfer Kit, for 40 blots #1704271). Membranes were blocked in 5% non-fat dry milk (SERVA Electrophoresis GmbH, #42590.01, Heidelberg, Germany) in Tris-buffer saline, 0.1% Tween20 (ITW Reagents S.R.L., #A4974,0500, Monza, MB, Italy) and incubated with the indicated antibodies (listed in Supplementary Materials and Methods) following the manufacturer’s instructions.

Western blot chemiluminescence reactions were visualized with ECL (Clarity Western ECL Substrate, Bio-Rad, #170–5060) using iBright™ CL1500 Imaging System (Thermo Fisher Scientific). Densitometric analyses performed using Bio-Rad Image lab software 6.1 version. Total proteins were normalized over total unphosphorylated protein when indicated or over actin or vinculin as loading control proteins. Data are expressed as relative expression values.

### Extracellular (e)NAMPT quantification

eNAMPT levels were detected in culture supernatants (SN) using human NAMPT ELISA kit (Adipogen, Epalinges, Switzerland; AG-45 A-0006YEK-KI01) and western blot, as described in Supplementary Material and Methods.

### Immunohistochemistry staining on Melanoma Tissue Microarray (TMA)

Immunohistochemistry was performed on a High-density tissue microarray (TMAs) including 129 formalin-fixed and paraffin-embedded (FFPE) samples of MCM (see Supplementary Material and Methods) using the Ventana automated stainer Discovery ULTRA. Section  (3 μm) were deparaffinized with EZ Prep (#950 − 102, Ventana Medical Systems, Tucson, AZ, USA) and antigen retrieval was carried out by incubation with Cell Conditioning Solution 1 (#950 − 124, Ventana Medical Systems), a Tris-based buffer. Sections were then incubated with the following primary antibodies: anti-NAMPT (mouse monoclonal, clone OMNI379, 20 A-0034, 1:300, Adipogen) and anti-PD-L1 (rabbit monoclonal, #13684, clone E1L3N, 1:50, Cell Signaling Technology).

The NAMPT signal was developed using an anti-mouse HRP detection system coupled with ChromoMap DAB (#760 − 159, ready-to-use, Ventana Medical Systems). The PD-L1 signal was developed using an anti-rabbit alkaline phosphatase detection system coupled with ChromoMap RED (#760 − 160, ready-to-use, Ventana Medical Systems). Sections were counterstained with Hematoxylin (#760–2208, ready-to-use, Ventana Medical Systems). Tissues were digitally scanned at 400× magnification using the Aperio AT2 platform (Leica Biosystems, Wetzlar, Germany). A semi-quantitative H-score analysis was performed to evaluate NAMPT staining in MCM tissue samples [32], as described in Supplementary Material and Methods.

### Genetic silencing

Silencing of target genes was performed using Lipofectamine 2000 (#11668-027 Invitrogen, Thermo Fisher Scientific) and specific siRNA BRD4 (#4390824, s23091), STAT1 (#4390824, s279), IRF1 (#4392420, s7501), NAMPT (AM16708, ID: 121562) all from Ambion, Thermo Fisher Scientific, while a negative control siRNA (Ambion Silencer Select Negative Control #4390843) was used as a control. The protocol was detailed in Supplementary Material and Methods. Two shRNA sequences targeting NAMPT (shA and shC), previously validated [33], along with a control shRNA sequence, were designed (see Supplementary Material and Methods). The shRNAs were cloned into the pLKO.1-Puro vector (Addgene plasmid #10878) and used for transient transfections with lipofectamine, as described for siRNA.

### Chromatin Immunoprecipitation and RT-PCR (ChIP)-RT-PCR

ChIP was performed on cultured cells using a standard protocol, detailed in Supplementary Material and Methods, using specific antibodies anti-BRD4 antibody (1:50, #13440), anti-IRF1 (1:50; #8478) or normal rabbit IgG (**#**2729) as control, all from Cell Signaling Technology. The following quantitative real-time PCR (qPCR) was performed using SYBR Green SuperMix (#1725271 Bio-Rad) and designed primers targeting five specific regions of the NAMPT promoter (see Supplementary Material and Methods). PD-L1 was used as positive control [34, 35]. The normalized fold enrichment (BRD4 over IgG or IRF1 over IgG) values for ChIP data were calculated with the ΔΔCt method from the Excel-based ChIP-qPCR analysis template [36].

The analysis of putative IRF1 and IRF2 binding motifs on NAMPT promoter were conducted using JASPAR2024 https://jaspar.elixir.no/. JASPAR is an open-access database of curated, non-redundant transcription factor (TF) binding profiles stored as position frequency matrices (PFMs) and TF flexible models (TFFMs) for TFs across multiple species in six taxonomic groups [37]. We selected “*vertebrata*” group and JASPAR Scan option, with a profile score threshold 80%, uploading FASTA sequencing of NAMPT promoter.

### Co-culture experiments followed by cytotoxicity and transwell assays

Splenocytes (SPC) were isolated, under sterile conditions, from the spleens of C57BL/6 HLA-A2.1 transgenic mice (Jackson Laboratory) [38]. Individual spleens were mechanically homogenized in DMEM supplemented with 10% FBS and 1mM HEPES. Cell suspensions were centrifuged at 1300 rpm for 10 min at room temperature (RT), then resuspended in red blood cell lysis buffer (155 mM NH4Cl, 15.8 mM Na2CO3, 1 mM EDTA, pH 7.3) and incubated for 45 s at RT. After lysis, cells were washed and resuspended to obtain single-cell suspensions. SPCs from individual mice were pooled prior to activation. For T-cell activation, 6-well plates were coated with purified anti-mouse CD3 antibody (BioLegend, San Diego, CA, #100208) at 1 µg/mL in sterile PBS and incubated at 37 °C in 5% CO2 for 2 h. Following removal of the coating solution, SPCs were resuspended in complete medium at a concentration of 3 × 10⁶ cells/mL and seeded into the pre-coated plates in the presence of soluble purified anti-mouse CD28 antibody (BioLegend, #102112) at 1 µg/mL. Cells were cultured for 72 h at 37 °C in a humidified incubator with 5% CO2. Flow cytometry analysis performed at the end of the activation protocol showed that T lymphocytes represented approximately 75% of CD45⁺ cells under these experimental conditions (data not shown).

Human HLA-A2^+^ 501MEL were treated for 48 h with IFN-γ 100 ng/ml, and in the last 24 h FK866 25 nM was added to the culture. For cytotoxicity assay, following treatment, target tumor cells were washed twice to remove the treatments, and co-cultured with pre-activated T cells derived from C57BL/6 HLA-A2.1 transgenic mice for 24 h at an effector to target ratio of 25:1. After co-culture, tumor cells were harvested and stained with 1 mg/mL 7-aminoactinomycin D (7-AAD; Beckman Coulter Life Sciences, #IM3630). Samples were acquired on a BD FACSCelesta flow cytometer and analyzed using FlowJo v10.5.3. Target tumor cell death was determined as the percentage of 7-AAD⁺ cells. We adopted a flow cytometry gating strategy based on forward and side scatter parameters to discriminate 501MEL melanoma cells from splenocytes. This gating approach was established and validated using single-population controls (tumor cells alone and splenocytes alone), as well as co-culture samples. Under these conditions, the two populations showed clear and reproducible separation, allowing reliable identification of tumor cells during the analysis. Spontaneous death was obtained by culturing target cells without T cells, and maximal cell death was obtained after treatment with 1% saponin. The percentage of specific lysis was calculated using the following formula: ([dead targets in sample (%) - spontaneously dead targets (%)]/ [dead target maximum (%) - spontaneously dead targets (%)]) x 100.

For the migration assay, pre-activated T cells derived from C57BL/6 HLA-A2.1 transgenic mice were resuspended in serum-free medium and 2.5 × 10⁵ cells were added to the upper chamber of Transwell inserts (8 μm pore size; Corning, #3422). The lower chambers contained pre-treated target melanoma cells (as described above) with their culture supernatants. Cells were incubated for 6 h at 37 °C in a humidified atmosphere containing 5% CO 2. After incubation, T cells that had migrated to the lower chamber were collected, centrifuged, resuspended in 300 µl DMEM and counted in a Burker chamber, using methylene blue to exclude dead cells. At least three independent experiments were performed on separate plates for both assays.

### Oxygen Consumption Rate (OCR) measurement with Resipher technology

Oxygen consumption rate (OCR) was measured using the Resipher System (Lucid Scientific, Atlanta, GA) equipped with a 32-sensor array (NS32-101 A, Lucid Scientific). Cells were seeded at a density of 5 × 10^3^ cells/well in a 96-well Falcon plate (Corning, CLS 353072), which is compatible with the Resipher insert. 501MEL and A375 cells were left to adhere overnight (~ 14–15 h) in serum-free medium (starvation condition), then treated with IFN-γ 100 ng/ml alone or in combination with FK866 25 nM or Metformin 5 mM (317240-5 mg Sigma Aldrich) in complete (10% FBS) medium. Doses were selected as reported in previous works [14, 39]. In a set of experiments OCR was measured in cells silenced for NAMPT or control cells for 48 h and stimulated with IFN-γ 100 ng/ml in the last 24 h. The sensor array was positioned on the plate, and OCR measurements were acquired continuously over 24 h starting at the beginning of the treatment. OCR values were subsequently normalized to OD measurements.

### Mitochondria isolation and activity of electron transport chain (ETC) complexes

Mitochondria were extracted according to [40] in 501MEL and A375 cells treated as described before in OCR method. A 100 µL aliquot was sonicated and used for the measurement of protein content and the enzymatic activities indicated below. The remaining not-sonicated part was used to measure the ETC activity. The rate of ETC from mitochondrial complex I (MCI) to complex III (MCIII) and the activity of complexes I were measured spectrophotometrically with a Synergy HT microplate reader (Bio-Tek Instruments, Winooski, VT) on non-sonicated mitochondria, as detailed in [41]. In a set of experiments MCI and ETC were measured in cells silenced for NAMPT or control cells for 48 h and stimulated with IFN-γ 100 ng/ml in the last 24 h. Results were expressed as nanomoles of reduced cytochrome c /min/mg mitochondrial proteins (ETC rate from complex I to complex III), nanomoles of NAD^+^/min/mg mitochondrial proteins (complex I activity).

### NDI1 transfection and treatment

Plasmid for NADH-ubiquinone reductase (H(+)-translocating known as NDI1 from S. cerevisiae pcDNA3.1/Ndi-1/myc-His was a gift from Shashi Jain & Dieter Wolf (plasmid # 127503; http://n2t.net/addgene:127503, Addgene, Watertown, MA, USA) [42].

NADH-ubiquinone reductase (H(+)-translocating) NDI1 overexpression was obtained by transient transfection of the pCDNA3.1/Ndi1/myc-His plasmid (Addgene 127503) in MCM cells. This plasmid encodes for S. cerevisiae *Ndi1* gene and allows the expression of NDI1 protein fused to myc and his tags at its C terminus. pCDNA3.1 empty vector was used as negative control. NDI1 expression was assessed 48 h post-transfection by WB analysis using an anti-Myc-Tag antibody (9B11) (Cell Signaling Technology #2276). Experimental design was detailed in Supplementary Material and Methods.

### Signatures score calculation and correlations on TCGA and CCLE datasets

IFN-γ pathway score (IFNG pathway score) was calculated as the geometric mean of the IFN-γ pathway genes in TCGA- Skin Cutaneous Melanoma SKCM samples (both primary and metastatic, *n* = 471 transcripts-per million (TPM) expression data and in the Broad Cancer cell lines encyclopedia (CCLE) [43] samples (*n* = 57) classified with the SKCM ONCOTREE code, expressed as reads per kilobase million (RPKM).

The gene sets used in this study were retrieved from: Protein Interaction Database [44], gene signatures for IFN gamma signaling and HUGO Gene Nomenclature Committee (HGNC) for mitochondrial complex I subunit (MCI).

Pancancer association analysis between *CD274* and *NAMPT* expression levels TCGA expression data were downloaded from https://gdac.broadinstitute.org/. Illumina Hiseq v2 RSEM gene normalized data were used for the association analysis. Expression data from [45] were downloaded from GEO, accession: GSE154996. The pre-post IFN-γ treatment delta value for NAMPT and CD274 was calculated by subtracting the FPKM of the pre-condition to the FPKM value of the post treatment.

To evaluate association between NAMPT expression data and ICI response outcomes, data were downloaded from [46]. Pairwise Wilcoxon Rank Sum test was performed for NAMPT levels in each ICB response category, using the wilcox.test() R function.

All statistical analysis of this part were performed with R statistical software (*R: A Language and Environment for Statistical Computing | BibSonomy*, n.d.) v 4.3.1. Correlation analysis was performed with *cor.test()* function with methods ‘*spearman’* and/or ‘*pearson’*.

### Statistical analysis

Statistical analyses were performed using GraphPad Prism version 9.0 (GraphPad Software Inc., La Jolla, CA, USA), if not specifically indicated. Statistical significance was assessed using an unpaired or paired Student’s t-test, following a Shapiro-Wilk test to evaluate the normality of data distribution. One-way ANOVA was also used where indicated.

Unless otherwise indicated, data in the Figures are presented as the mean ± SEM. For all statistical tests, the 0.05 level of confidence was accepted for statistical significance. Significance was represented as: * *p* ≤ 0.05, ** *p* ≤ 0.01, *** *p* ≤ 0.001, **** *p* ≤ 0.0001.

## Results

### IFN-γ signaling activation induces NAMPT mRNA and protein expression in melanoma cell lines

NAMPT as intracellular NAD-biosynthetic enzyme is emerging as an IFN-γ-inducible gene in monocytes/macrophages [25, 26, 47] and in some cancer cell lines [28–30, 48]. We aimed to investigate in depth the regulation of NAMPT expression via IFN-γ in our cellular models. For this purpose, we selected three human melanoma cell lines BRAFV600E 501MEL, A375 and M14 in which we first assessed the expression levels of interferon gamma receptor 1 (*IFNGR1*). 501MEL cells were endowed with the highest receptor expression, whereas in A375 and M14 the levels were comparably lower (Supplementary Fig. 1A). Time-kinetic analysis revealed that *NAMPT* mRNA transcription peaked after 24 h-IFN-γ treatment in all cell lines (Supplementary Fig. 1B). For subsequent experiments, we selected mainly 501MEL and A375 cells.

**Fig. 1:**
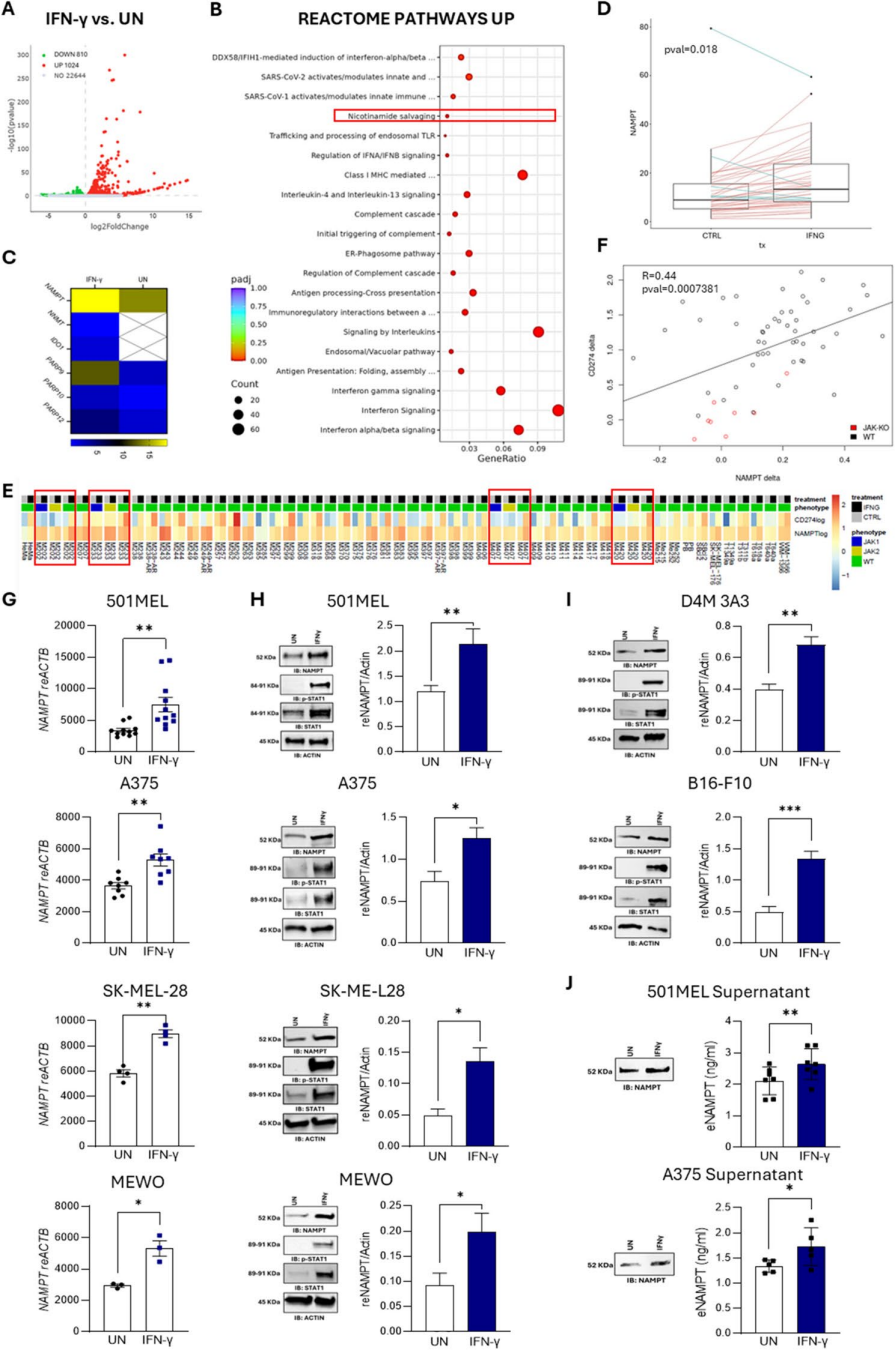
NAMPT upregulation is a defining feature of the IFN-γ-driven gene program in melanoma cells.** A** Volcano plot showing differentially expressed genes (|log2(FoldChange)| >= 0 & pvaladj < = 0.05) in IFN-γ–treated (100 ng/ml, 24 h) vs. untreated (UN) 501MEL cells. Red and green dots indicate up- and downregulated genes, respectively; grey dots indicate non-significant (NO) genes. Y-axis: −log₁₀ P values; X-axis: log₂ fold change. **B** Bubble plots of enriched Reactome pathways in IFN-γ–treated 501MEL cells. Bubble size and color indicate the number and significance of enriched genes, respectively. Red rectangle highlights nicotinamide pathway. **C** Heatmap of IFN-γ–regulated genes in the nicotinamide/nicotinate pathway. FPKM values range from white (undetectable) to blue (low) to yellow (high expression). **D** Box plot representing the *NAMPT* levels in 48 human melanoma cell lines published (dataset GSE154996) untreated (CTRL) or treated with IFN-γ (IFNG). Pval calculated with the Wilcoxon test is reported. **E** Heatmap representing the log10 FPKM of *NAMPT* and *CD274* in untreated (CTRL, grey) and IFNG treated (black) conditions for the different cell lines, with WT background (green) or JAK1 KO (blue) or JAK2 KO (yellow). Red rectangles highlight the 4 cell lines reporting expression values in WT and JAK1/JAK2 KO variants. Data are derived from dataset GSE154996. **F** Scatter plot of the pre-post *NAMPT* and *CD274* expression delta for WT (black), JAK1/JAK2 KO melanoma cell lines (red). The full black line shows the linear regression line between the two deltas, highlighting a positive correlation calculated with Spearman method. Data are derived from dataset GSE154996. **G** Histograms representing *NAMPT* mRNA in 501MEL (*n* = 11); A375 (*n* = 8); SK-MEL-28 (*n* = 4) and MEWO (*n* = 3) cells after IFN-γ treatment (100 ng/ml, 24 h) vs. untreated (UN). Data are reported as relative expression values (*NAMPT/ACTB*). Bar graphs show mean ± SEM. **H-I** Representative blots for NAMPT, pSTAT1 and STAT1 protein levels in human MCM cell line (**H**) 501MEL, A375, SK-MEL-28, MEWO, and murine MCM (**I**) D4M 3A3 and B16-F10. On the right of the immunoblots, histograms reporting the cumulative data of NAMPT protein levels. Data are reported as relative expression values (NAMPT/Actin). pSTAT1/STAT1 confirmed IFN-γ pathway activation. Bar graphs show mean ± SEM (≥ 4 replicates). **J** eNAMPT levels (ng/ml) measured by ELISA assay after IFN-γ exposure (200 ng/ml, 48 h) vs. UN in 501MEL (*n* = 7) and A375 (*n* = 5). Bar graphs show mean ± SEM. On the left, representative immunoblots of soluble eNAMPT detected by western blot in 10x concentrated supernatants (200 ng/ml, 24 h). Three independent experiments were performed. Statistical analysis: paired t-test (*p ≤ 0.05, **p ≤ 0.01, ***p ≤ 0.001)

To identify gene signatures activated by IFN-γ in melanoma cells, we first carried out RNA sequencing (RNA-seq) followed by a comprehensive analysis of transcriptional profiles in 501MEL cells, which were found to be the most responsive to IFN-γ. IFN-γ-treated cells displayed 1824 differentially expressed genes relative to untreated cells. Among these, 1024 genes were upregulated and 810 downregulated (Fig. 1A and Supplementary materials). Interrogation of the upregulated genes set using functional annotation tools confirmed an enrichment for Gene Ontologies (GO) biological processes (Supplementary Fig. 2A) and Reactome pathways (Fig. 1B) classically associated with IFN signaling (Supplementary materials). Most significantly enriched categories were response to virus (pval 1,16E-22); activation of immune response (pval 9,46E-14); interferon signaling (pval 1,40E-21); signaling by interleukins (pval 1,09E-07); antigen processing (pval 9,46E-09). Apolipoprotein L6 (*APOL6*) emerged as the individual gene that showed the highest fold change in response to IFN-γ (*log2FC 14.47)*, which is consistent with recent findings obtained in tumors derived from patients that display strong immunotherapeutic responses across various types of cancer [49]. As expected, Interferon-induced transmembrane protein 1 (*IFITM1*,* log2FC 11.18*), *CXCL10 (log2FC 10.92)*, *IRF1 (log2FC 4.64)*,* STAT1 (log2FC 2.14)* were among the most significantly upregulated genes. Interestingly, genes belonging to the nicotinate metabolism/nicotinamide salvaging pathway including *NAMPT*, *NNMT*, *PARP14*, *PARP9*, *PARP10*, *IDO1* (pval 0.0002, Fig. 1B, red rectangle) emerged among the upregulated gene, as shown in the heatmap of Fig. 1C. Although *NAMPT* was expressed already under basal conditions (untreated, UN), its mRNA levels were further increased by IFN-γ exposure. On the contrary, *NNMT* and *IDO1* mRNAs were almost undetectable in UN conditions and increased significantly after treatment. Notably, IFN-γ-upregulated genes also comprise genes within the Toll-like Receptor Cascades category (pval 0.0004, 21 genes included), which have been linked to the activation of the immunomodulatory NF-kB signaling pathway [17] (Supplementary materials). Downregulated categories were mostly related to processes such as cell adhesion, wound healing and cellular movement as shown in Supplementary Fig. 2B, C.

To explore if the induction of NAMPT expression via IFN-γ could also be found in multiple melanoma cell lines, we analyzed the published dataset GSE154996 [45] reporting RNAseq data of 48 human melanoma cell lines either untreated or treated with IFN-γ. These data indicated that vast majority of cells upregulated *NAMPT* expression (pval 0.018) following IFN-γ exposure (Fig. 1D). Importantly, as shown in the heatmap of Fig. 1E and in Fig. 1F, cell lines that had *JAK1* (blue box) or *JAK2* (yellow box) natural or induced loss-of-function mutations and that were unable to signal through the IFN-γ receptor displayed a reduction in IFN-γ-induced NAMPT expression. Of note, in IFN-γ-responsive cell lines, NAMPT relative induction showed a strong positive correlation with CD274/PD-L1 (R 0.44, pval 0.0007381, Fig. 1F).

The validation of IFN-γ-induced NAMPT expression observed in RNA-seq data was carried out in four human melanoma cell lines, 501MEL, A375 SK-MEL-28 (BRAF V600E) and MEWO (BRAF WT), and two murine melanoma cell lines, D4M 3A3 (BRAF V600E) and B16-F10 (BRAF WT), either untreated or after exposure to IFN-γ for 24 h. All cell lines displayed significant upregulation of both *NAMPT* mRNA (Fig. 1G) and protein (Fig. 1H, I). As readouts of IFN-γ signaling activation we also monitored the phosphorylation status of STAT1 (Fig. 1H, I and Supplementary Fig. 3A-D and Supplementary Fig. 4A, B), along with the expression of two IFN-γ-inducible genes, *CXCL10* and *CD274* (Supplementary Fig. 4C). Total STAT1 protein levels were also found increased upon IFN-γ stimulation (Fig. 1H, I), consistently with previous reports [50].

The measurements of NAD levels in cell lines treated with IFN-γ for 24 h did not reveal significant differences in total NAD relative to untreated cells (data not shown), consistent with the possibility that increases in NAMPT-dependent NAD biosynthesis could be balanced by increased NAD consumption, as previously reported [48]. However, we cannot rule out the occurrence of IFN-γ-induced increases of NAD levels within specific subcellular compartments.

Moreover, upon IFN-γ exposure the increases in intracellular NAMPT levels were also coupled to an increased release of soluble eNAMPT in both cell lineages (Fig. 1J).

Overall, these data indicate that several genes within the NAD metabolic network are upregulated upon IFN-γ–mediated signaling activation, and that the induction of NAMPT expression is a defining feature of the IFN-γ gene program in melanoma cells, independent of BRAF mutational status. Remarkably, IFN-γ exposure triggers transcriptional and metabolic changes that at least partially overlap with those observed during the metabolic adaptation of BRAFi-resistant melanoma cells, in which NAMPT plays a central role [15, 17].

### Expression of NAMPT is positively correlated with IFN-γ-signaling activation and PD-L1 expression in melanoma patients

**Fig. 2:**
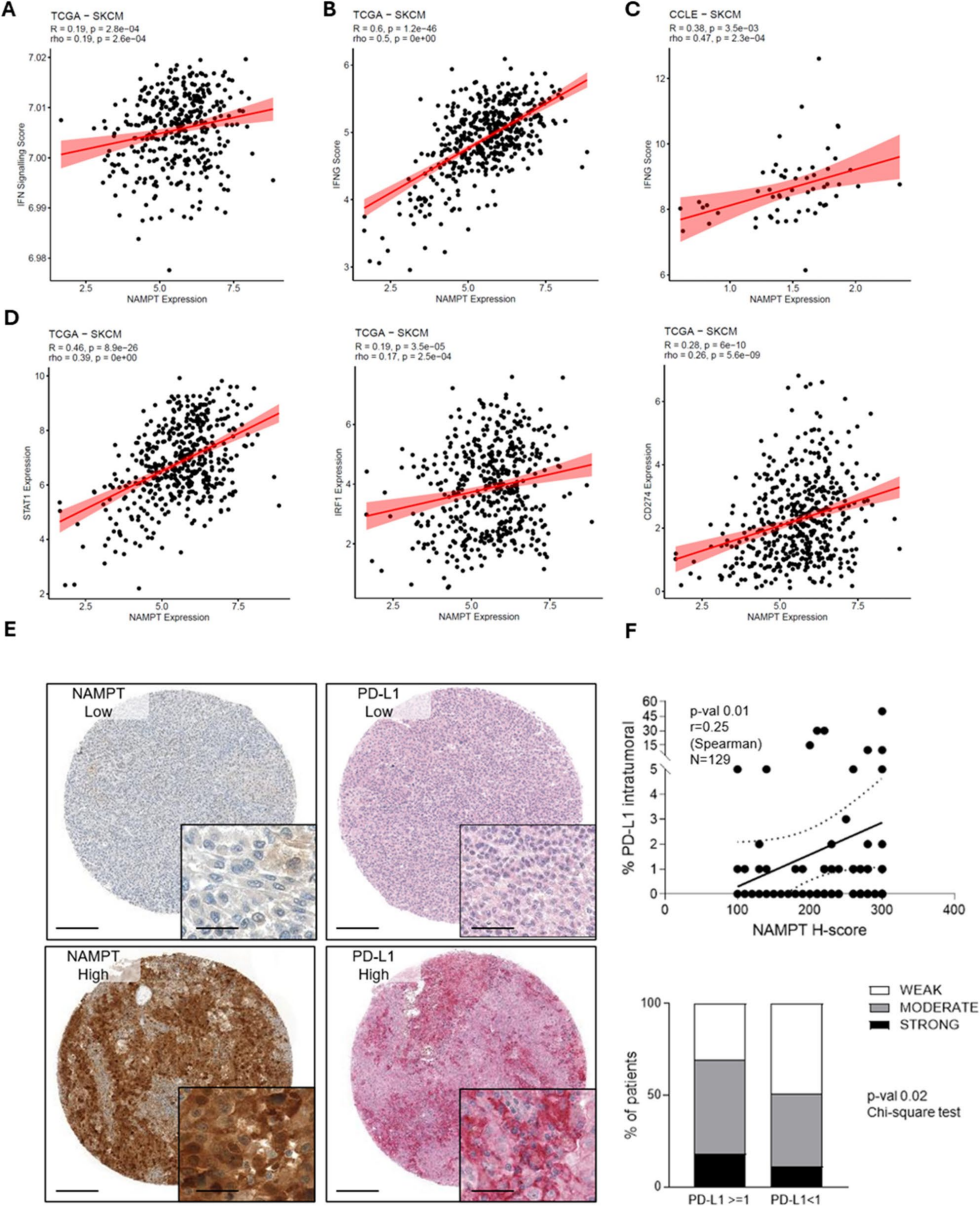
NAMPT expression strongly correlates with PD-L1 levels in melanoma. **A-C** Scatter plots correlating *NAMPT* expression and IFN signaling (**A**), or IFNG score (**B**) in TCGA Skin Cutaneous Melanoma SKCM cohort (*n* = 471). In (**C**) the correlation is performed in melanoma cell lines included in CCLE SKCM (*n* = 57). Pearson (R) and Spearman (rho) correlations and p-value are shown. **D** Scatter plots correlating *NAMPT* expression and *STAT1*, *IRF1*, and *CD274*, respectively. Each dot represents a sample of the TCGA SKCM cohort (*n* = 471). Pearson (R) and Spearman (rho) correlations and p-value are shown. The expression of all three genes positively correlates with *NAMPT* expression. **E** Representative IHC staining for NAMPT in PD-L1-negative melanoma samples (top panels) and NAMPT in PD-L1-positive melanoma samples (bottom panels). As shown in the representative figures NAMPT expression correlates with PD-L1 expression. Magnification 10×, inset 20×; scale bars: 100 μm and 50 μm, respectively. **F** Correlation between % of PD-L1 intratumoral expression and NAMPT expression (reported as NAMPT H-score divided into weak, moderate and strong expression) derived from TMA IHC analysis. Spearman (rho) correlations and p-value are shown. The graph below shows the percentages of patients expressing NAMPT (three different scores) in MCM patients divided according to the % of PD-L1 expression (≥ 1 or < 1). Chi-square test was used to determine the p-value

To further extend the data obtained in melanoma cell lines, we decided to analyze the The Cancer Genome Atlas Program (TCGA) melanoma cohort (SKCM metastatic and primary samples), and melanoma cell lines included in Cancer Cell Line Encyclopedia (CCLE). We observed a significant association between global IFN signaling score and *NAMPT* expression in Skin Cutaneous Melanoma SKCM-TCGA cohort (*n* = 103 primary and 368 metastatic samples, Fig. 2A). The analysis correlating IFN**-γ** (IFNG) score and *NAMPT* expression appeared stronger (R 0.6, pval 1.2e-46) in SKCM-TCGA (Fig. 2B), and this association was confirmed also by analysis of the 57 SKCM cell lines in CCLE dataset (Fig. 2C). Consistently, we also showed in SKCM-TCGA cohort a high positive correlation with the IFN-γ-induced genes, i.e., *STAT1*, *IRF1* and *CD274*/PD-L1 and *NAMPT* expression (Fig. 2D). The correlation *NAMPT*/*STAT1* emerged as the most robust (R 0.46, pval 8.9e-26). It has been reported that upon IFN-γ signaling activation, STAT1 binds to an enhancer element within the *NAMPT* promoter, thereby driving its transcription in tumor-associated macrophages [26]. *CD274*/PD-L1, extensively implicated in immune evasion mechanisms of tumors [51], was previously linked to NAD metabolism. In fact, Lv et al. found that the boost of NAD/NAMPT activity synergizes with IFN-γ/IRF1 signaling, in regulating PD-L1 expression in multiple tumors and this promotes immune evasion [28]. At the mRNA level in TCGA-SKCM cohort we obtained a significant correlation (R 0.28, pval 6e-10, Fig. 2D) between NAMPT/PD-L1 expression.

Notably, the correlation between NAMPT and *CD274*/PD-L1 expression was found to be positive and significant across all tumor types included in TCGA dataset, except for Pheochromocytoma, Paraganglioma (PCPG) and Testicular Germ Cell Tumors (TGCT), as reported in Supplementary Table 1.

To verify this correlation at the protein level in tumor tissue derived from melanoma patients, we also analyzed a melanoma tumor tissue microarray (TMA) composed by 129 samples. Immunohistochemistry analysis confirmed the correlation NAMPT^high^/PD-L1^high^ expressing patients vs. the NAMPT^low^/PD-L1^low^ ones (Fig. 2E, F). This analysis revealed that patients with a NAMPT moderate/strong score are more represented within the cohort with PD-L1 ≥ 1% (Fig. 2F).

Together, these results strongly support the connection between elevated levels of NAMPT and PD-L1 expression in melanoma as well as in the majority human cancers.

### BRD4 regulates NAMPT expression

**Fig. 3:**
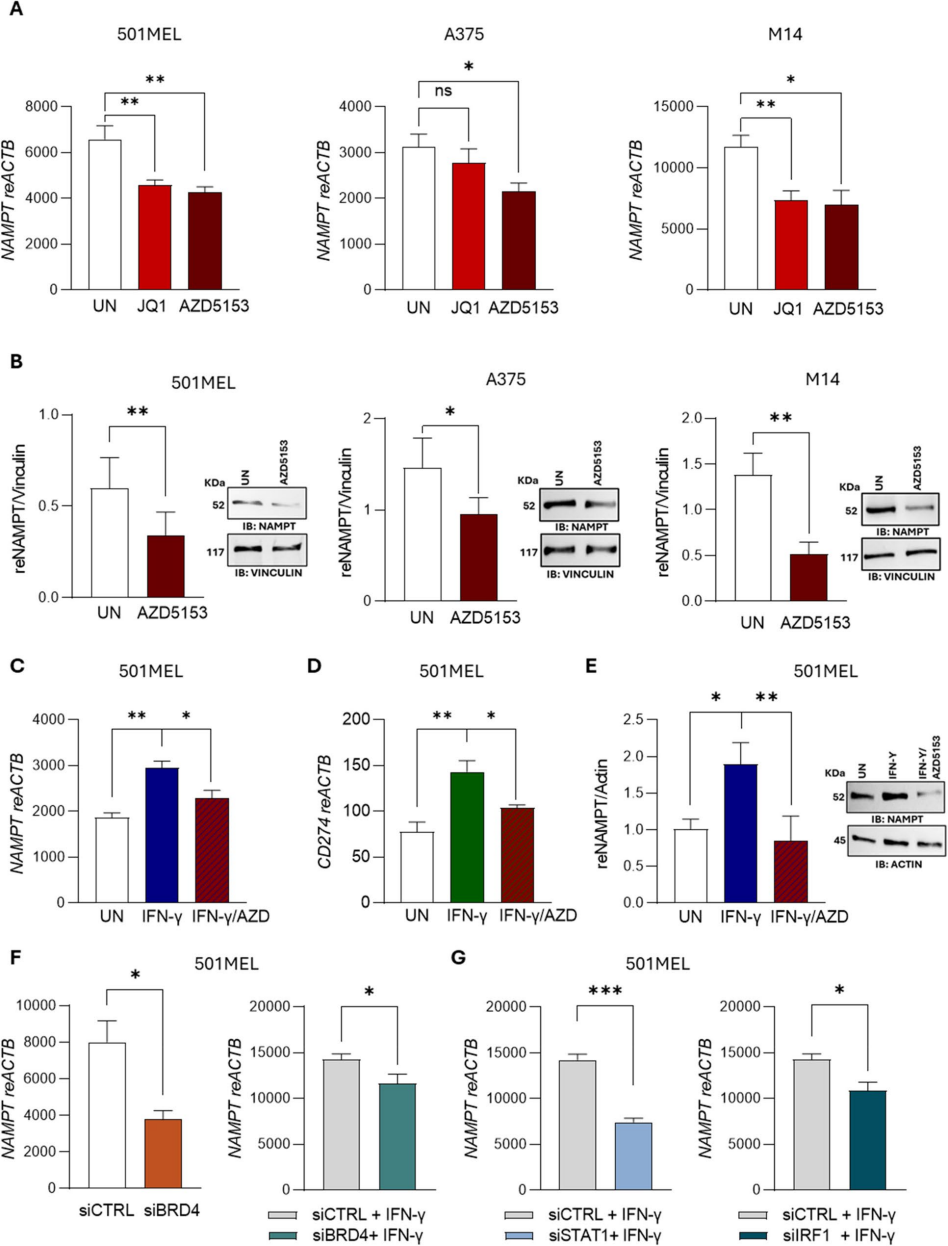
BRD4/STAT1/IRF1 modulation regulates basal and inducible NAMPT expression. **A** Histograms representing *NAMPT* mRNA levels in 501MEL, A375, and M14 melanoma cells after 24 h of treatment with JQ1 (5 µM) or AZD5153 (100 nM) vs. untreated (UN) (*n* ≥ 4). Data are reported as relative expression values (*NAMPT/ACTB*). One-way ANOVA, multiple comparisons over UN: *p ≤ 0.05, **p ≤ 0.01, ***p ≤ 0.001, ****p ≤ 0.0001. **B** NAMPT protein expression in the same cell lines by immunoblot. Data are reported as relative expression values (NAMPT/Vinculin; *n* ≥ 4 replicates); representative blots on the right. Paired t-test vs. AZD5153: *p ≤ 0.05, **p ≤ 0.01, ***p ≤ 0.001. **C** *NAMPT* mRNA expression in 501MEL cells after treatment with IFN-γ (100 ng/ml) alone or combined with AZD5153 (100 nM) for 24 h (*n* ≥ 4). Data are reported as relative expression values (*NAMPT/ACTB*). One-way ANOVA, multiple comparisons over IFN-γ: *p ≤ 0.05, **p ≤ 0.01, ***p ≤ 0.001, ****p ≤ 0.0001. (D) *CD274*/PD-L1 mRNA expression in 501MEL cells under the same conditions described in C. *CD274*/PD-L1 is used as positive control, (*n* ≥ 4). Data are reported as relative expression values (*CD274/ACTB*). One-way ANOVA, multiple comparisons over IFN-γ **p ≤ 0.01). **E** NAMPT protein expression in 501MEL cells by immunoblot; data are reported as relative expression values (NAMPT/Actin; *n* = 7). One-way ANOVA, multiple comparisons over IFN-γ-: **p ≤ 0.01. **F** *NAMPT* mRNA expression in 501MEL cells after 48 h of BRD4 silencing (siBRD4) vs. control siRNA (siCTRL) at basal condition (graph on the left) or treated with IFN-γ (24 h; 100 ng/ml). Data are reported as relative expression values (*NAMPT/ACTB*; n = 4); unpaired t-test: *p ≤ 0.05. **G** *NAMPT* mRNA expression in 501MEL cells after 48 h in the presence of siSTAT1 (graph on the left) or siIRF1 followed by IFN-γ (24 h; 100 ng/ml) in ≥ 4 replicates. Data are reported as relative expression values (*NAMPT/ACTB*; *n* = 4); unpaired t-test: *p ≤ 0.05. All bar graphs show mean ± SEM

Since our data indicate NAMPT as an IFN-γ-inducible gene, this prompted us to investigate the molecular basis of NAMPT induction in melanoma cells. In tumor-associated macrophages, STAT1 was shown to be implicated in the transcriptional regulation of *NAMPT* downstream of IFN-γ. This occurs via binding of STAT1 to a conserved element located within the first intron of the *NAMPT* gene, thereby termed Nampt-Regulatory Element-1 (NRE1) [26, 30]. However, it is likely that additional mechanisms contribute to NAMPT regulation downstream of IFN signaling, and the epigenetic bases of NAMPT regulation are currently unknown. Given the strong co-expression of NAMPT and PD-L1 in melanoma patients and in the majority human cancers, and because PD-L1 is regulated at the transcriptional level via IFN-γ/IRF1 axis through the BET epigenetic factor BRD4 [35, 52, 53], we hypothesized that a similar mechanism may also take place in IFN-γ induced NAMPT expression in melanoma cells. BET proteins make up a highly conserved family of epigenome readers that play pivotal roles at the interface between chromatin remodeling and transcriptional regulation. By recognizing acetylated lysine residues on histones, BET proteins recruit chromatin-modifying enzymes to target promoters, where they may either act as co-activators or co-repressors [54]. BRD4 in particular, is known for its role in super-enhancers organization and oncogenes expression regulator in cancer [55]. Moreover, BRD4 was implicated in tumor maintenance in melanoma by promoting the expression of key cell-cycle and survival regulatory genes [56]. To investigate if BRD4 contributes to NAMPT expression regulation, we adopted different complementary approaches. JQ1 is a pan-BET proteins inhibitor that preferentially inhibits BRD4 [52, 57, 58], while AZD5153 is a potent and specific BRD4 inhibitor [59]. Treatment of 501MEL, A375 and M14 cells for 24 h with these compounds induced downregulation of basal NAMPT expression at both the mRNA (Fig. 3A) and protein levels (Fig. 3B and Supplementary Fig. 5A). In 501MEL, the cell line most responsive to IFN-γ, treatment of cells for 24 h with JQ1 and AZD5153 in combination with IFN-γ attenuated overall *NAMPT* mRNA expression levels (Fig. 3C and Supplementary Fig. 5B). As expected, in these cells we also observed a significant downregulation of the PD-L1 encoding *CD274* gene (Fig. 3D), used here as readout of BRD4 inhibition [35, 52, 53]. Of note, BRD4 inhibitors also caused a reduction of IFN-γ-inducible NAMPT protein level, and this effect was more pronounced with the AZD5153 compound (Fig. 3E and Supplementary Fig. 5C). Of note, not only BRD4 pharmacological blockade but also its siRNA-mediated gene knockdown (Supplementary Fig. 5D) obtained a similar result in 501MEL cells, causing a reduction of both constitutive- and IFN-γ-induced *NAMPT* expression (Fig. 3F). To assess the potential regulation of IFN-γ-inducible NAMPT by STAT1 and IRF1, we transiently silenced each transcription factor via siRNA in 501MEL cells exposed to IFN-γ for 24 h (Supplementary Fig. 5E). Compared to control conditions, knockdown of both STAT1 and IRF1 significantly blunted the *NAMPT* mRNA induction in response to IFN-γ stimulation (Fig. 3G). Silencing STAT1 resulted in a more pronounced decrease in *NAMPT* transcript levels, indicating its direct involvement in the transcriptional regulation of NAMPT expression, as previously reported [26, 30].

Overall, both pharmacological and genetic manipulation of the epigenetic reader BRD4 impacts on the regulation of constitutive- and IFN-γ-inducible NAMPT mRNA and protein expression, and both STAT1 and IRF1 transcription factors are implicated in the regulation of NAMPT expression.

### *NAMPT* is a BRD4/IRF1 target gene

It has been shown that BRD4 and IRF1 cooperate in triggering PD-L1 expression downstream of IFN-γ by forming a functional complex at the regulatory regions of the *CD274* gene [35, 49, 56]. We therefore investigated whether BRD4 and IRF1 could also be recruited to the *NAMPT* promoter upon IFN-γ treatment, thereby mirroring the regulatory mechanism described for PD-L1.

**Fig. 4:**
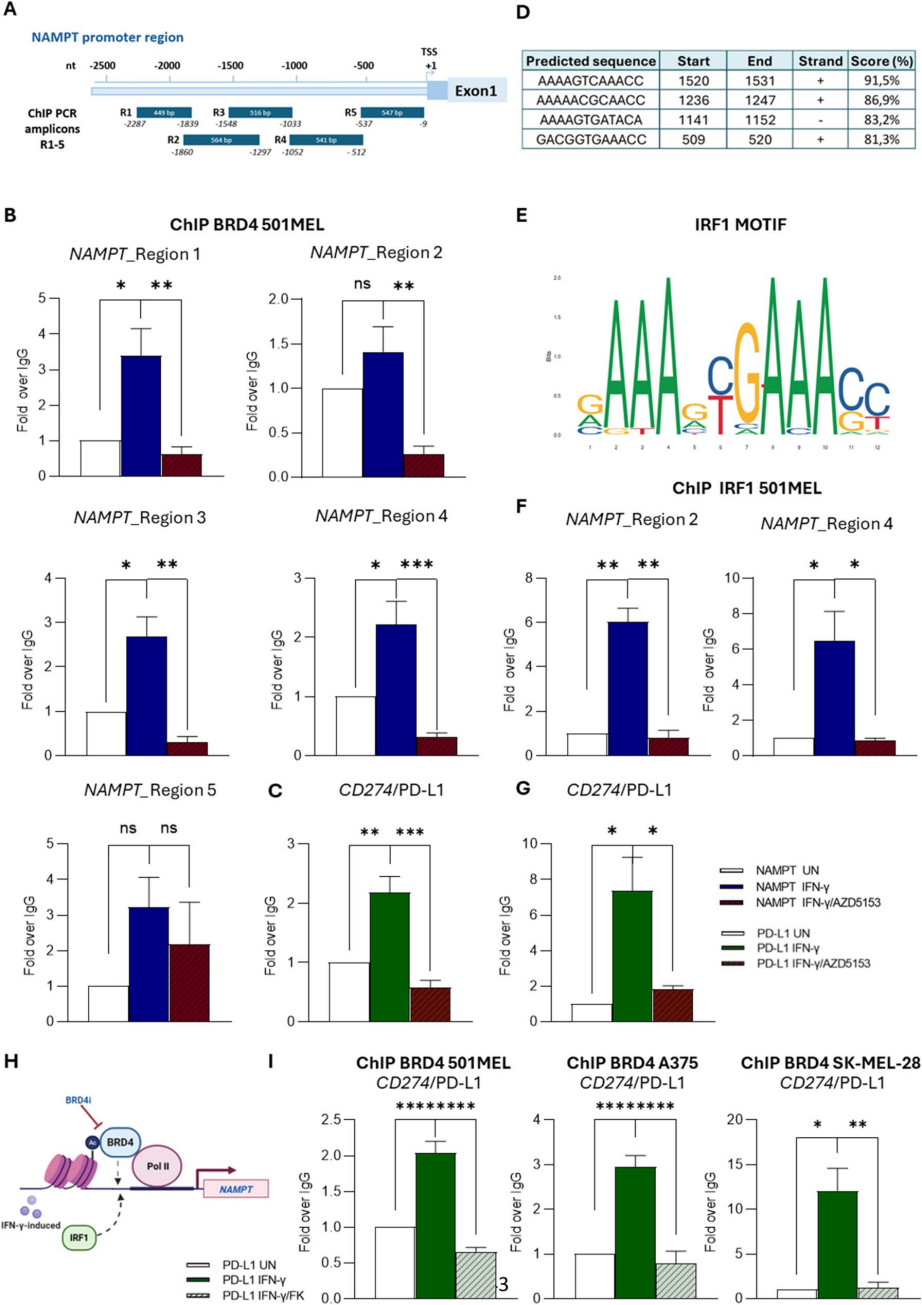
*NAMPT* is a direct target gene of BRD4 and IRF1. **A** Scheme of the *NAMPT* promoter divided in 5 regions covered by specific primers used in ChIP-qPCR. **B** Graphs showing the results of BRD4 ChIP-qPCR experiment (*n* = 3) in 501MEL cells. ChIP–RT-qPCR results are expressed as fold enrichment over negative control IgG used in ChIP and normalized to untreated cells (UN). Graphs represent the effects of IFN-γ (100 ng/ml) treatment alone or in combination with AZD5153 (100nM) for 24 h on BRD4 binding capacity to *NAMPT* promoter regions. **C** *CD274*/PD-L1 was used as a positive control of BRD4 activity for ChiP-qPCR experiment. Treatment conditions as in B. **D** The table reports the predicted IRF1 binding sites on the *NAMPT* promoter, as identified by JASPAR, indicating the corresponding score percentage. **E** Consensus sequence of the IRF1 binding motif located in the promoter region of *NAMPT*, determined by in silico motif analysis. **F** Graphs showing the results of IRF1 ChIP-qPCR experiment (*n* = 3). ChIP–RT-qPCR results are expressed as fold enrichment over negative control IgG used in ChIP and normalized to untreated cells (UN). Graphs represent the effects of IFN-γ (100 ng/ml) treatment alone or in combination with AZD5153 (100 nM) for 24 h on the IRF1 binding to *NAMPT* promoter regions. **G** *CD274*/PD-L1 was used as a positive control for IRF1 ChiP-qPCR experiment, as in C. **H** Model reporting the regulation of *NAMPT* transcription via BRD4 and IRF1 upon IFN-γ signaling activation. Pharmacological BRD4 inhibition using JQ1 or AZD5153 blocks BRD4/IRF1 *NAMPT* promoter occupancy. Figure made with BioRender.com. **I** Graphs showing the results of BRD4 ChIP-qPCR experiment in 501MEL (*n* = 3), A375 (*n* = 2) and SK-MEL-28 (*n* = 1). ChIP–RT-qPCR results are expressed as fold enrichment over negative control IgG used in ChIP and normalized to untreated cells (UN). Graphs represent the effects of IFN-γ (100 ng/ml) treatment alone or in combination with FK866 (25 nM) for 24 h on the BRD4 binding capacity to *CD274*/PD-L1 promoter region, as assessed by ChiP-qPCR. One-way ANOVA was used to assess statistical significance, multiple comparisons over the IFN-γ condition in all ChIP-qPCR experiments (*p ≤ 0.05, **p ≤ 0.01, ***p ≤ 0.001, ****p ≤ 0.0001). All bar graphs show mean ± SEM

To this end, we performed ChIP-qPCR analysis of BRD4 in 501MEL cells to determine its binding to the *NAMPT* promoter following IFN-γ stimulation. The *NAMPT* promoter was divided into five regions covered by specific primers, as depicted in Fig. 4A. IFN-γ treatment significantly increased BRD4 binding across multiple NAMPT regulatory regions (regions 1–4, Fig. 4B), and this effect was markedly reduced upon treatment with the BRD4 inhibitor AZD5153 (most prominently in region 4, Fig. 4B). *CD274*/PD-L1 was used as a positive control for BRD4 binding (Fig. 4C). Notably, BRD4 protein expression remained unchanged following IFN-γ treatment (Supplementary Fig. 5F). Comparable results were obtained in A375 cells (Supplementary Fig. 6A, B).

To further investigate the involvement of IRF1 in NAMPT regulation, we performed motif analysis of the *NAMPT* promoter using the JASPAR algorithm [36]. This analysis identified four putative IRF1 consensus binding sites (relative profile score threshold 80%), three located on the positive strand and one on the negative strand (Fig. 4D, E). These sites were primarily mapped to promoter Regions 2 (highest score) and 4. Interestingly, the first IRF1 consensus site overlapped with an IRF2 binding motif (GAAAAGTCAAACCATCTC), suggesting potential binding by IRF2.

To experimentally validate IRF1 recruitment, ChIP analysis was performed in 501MEL cells using an anti-IRF1 antibody followed by RT-qPCR targeting all the regions of *NAMPT* promoter. Consistent with JASPAR predictions, IFN-γ treatment strongly enhanced IRF1 binding, particularly at region 2 (Fig. 4F; Supplementary Fig. 6C). This recruitment was significantly impaired by AZD5153 treatment. These findings indicate that *NAMPT* transcription is regulated by coordinated BRD4 and IRF1 recruitment to its promoter in response to IFN-γ signaling, in a manner analogous to *CD274*/PD-L1 regulation (Fig. 4G). As summarized in Fig. 4H, these data support the notion that NAMPT is a direct transcriptional target of the BRD4/IRF1 complex.

Importantly, co-treatment of cells (501MEL, A375 and SK-MEL-28) with IFN-γ and the NAMPT inhibitor FK866 significantly reduced BRD4 binding to both the *CD274*/PD-L1 and *NAMPT* promoters (Fig. 4I and Supplementary Fig. 7A), in the absence of a parallel decrease in the abundance of total BRD4 protein (Supplementary Fig. 7B). These findings indicate that NAMPT catalytic activity is required for efficient BRD4 recruitment to IFN-responsive regulatory regions. Thus, NAMPT is not only induced downstream of IFN-γ signaling but also contributes to maintaining the transcriptional competence of IFN-γ target genes. Together, these results support a model in which the BRD4/IRF1/NAMPT axis operates as a feed-forward regulatory circuit integrating transcriptional and metabolic control of the IFN-γ response.

### NAMPT activity regulates IFN-γ-signaling activation

**Fig. 5:**
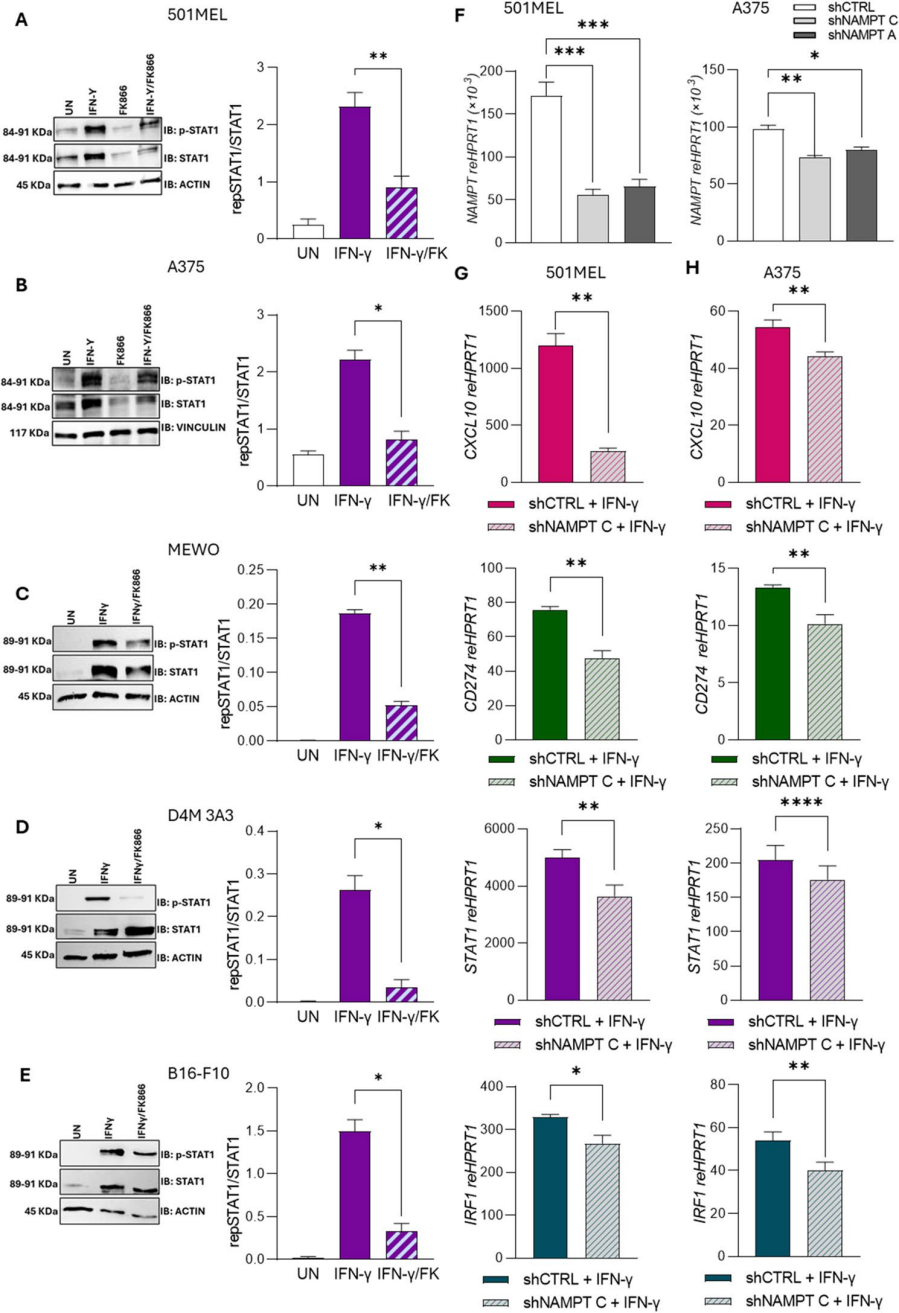
NAMPT activity inhibition and genetic silencing reduce IFN-γ-signaling activation.** A-E** Representative western blot analysis of pSTAT1 and STAT1 expression in human and murine MCM cell lines (501MEL, A; A375, B; MEWO, C; D4M 3A3, D and B16-F10, E) treated with IFN-γ (100 ng/ml) alone or in combination with FK866 (FK, 25 nM) for 24 h. Histograms, on the right, show cumulative data from band quantification of pSTAT1, expressed as relative expression total STAT1 protein levels (pSTAT1/STAT1). Data are reported as relative expression values. Bar graphs show mean ± SEM (≥ 4 replicates. Paired T-test was used to calculate statistical significance (*p ≤ 0.05, ** *p* ≤ 0.01, *** *p* ≤ 0.001). **F** Histograms report *NAMPT* mRNA expression levels in 501MEL (left graph) and A375 (right graph) after 48 h of transient *NAMPT* genetic silencing with two different shNAMPT (**A** and **C**) or shCTRL. *NAMPT* mRNA expression was normalized over *HPRT1* mRNA expression levels in both cell lines and represented in the graphs as relative expression (*NAMPT/HPRT1*) values divided by 1000 (×10⁻^3^) for better visualization. One-way ANOVA, multiple comparisons over siCTRL, was used to assess statistical significance (*p ≤ 0.05, **p ≤ 0.01, ***p ≤ 0.001, ****p ≤ 0.0001). **G-H** Histograms report *CXCL10*,* CD274*, *STAT1*, and *IRF1* mRNA expression levels in 501MEL (**G**) and A375 (**H**) cells silenced for *NAMPT* (shNAMPT C, 48 h) and treated with IFN-γ in the last 24 h (100 ng/ml). Data are reported as relative expression values (*target gene*/*HPRT1*) in both cell lines. Data from 4 independent experiments for each line. Paired T-test was used to calculate statistical significance (*p ≤ 0.05, **p ≤ 0.01, ***p ≤ 0.001, ****p ≤ 0.0001). Bar graphs show mean ± SEM (≥ 4 replicates)

To test the hypothesis that NAMPT activity may function not only as a downstream effector but also as a regulatory component of the IFN-γ network, we investigated the impact of NAMPT/NAD axis manipulation on IFN-γ signaling. We analyzed a panel of melanoma cell lines, including BRAFV600E 501MEL and A375 (human), D4M 3A3 (murine), and BRAF WT MEWO (human) and B16-F10 (murine), treated with IFN-γ alone or in combination with the NAMPT inhibitor FK866 (25 nM) for 24 h. This treatment condition effectively inhibits NAD biosynthesis without affecting cell viability [14]. FK866 treatment significantly reduced IFN-γ-induced STAT1 phosphorylation and total STAT1 protein levels (Fig. 5A-E; Supplementary Fig. 8A-E). To complement these findings, we performed genetic silencing of *NAMPT* in 501MEL and A375 cells using two independent shRNA constructs (shA NAMPT and shC NAMPT), alongside a scrambled control (shCTRL), as previously validated [33]. NAMPT expression was significantly reduced 48 h after transfection, as confirmed by RT-qPCR and WB (Fig. 5F; Supplementary Fig. 9A), and cells were subsequently stimulated with IFN-γ for 24 h. Under these conditions, NAMPT silencing resulted in a marked reduction in IFN-γ–induced expression of *CXCL10*, *CD274*/PD-L1, *STAT1*, and *IRF1* mRNA in both cell lines (Fig. 5G, H; Supplementary Fig. 9B, C).

Taken together, these results demonstrate that both pharmacological inhibition and genetic silencing of NAMPT impair key downstream components of the IFN-γ signaling program in melanoma cells.

### IFN-γ-induced oxygen consumption is reverted by NAD/NAMPT-dependent inhibition of complex I activity

We hypothesized that the reciprocal regulation between NAMPT/NAD axis and IFN-γ signaling occurs at the metabolic level, where IFN-γ-inducible NAMPT serves to secure adequate NAD levels to support mitochondrial respiration, a process in which mitochondrial complex I (MCI, *NADH: ubiquinone oxidoreductase*) has a central role [60, 61]. In monocytes/macrophages, MCI-dependent regeneration of NAD regulates IFN-γ-signaling [23], and IFN-γ-mediated gene expression promotes a respiratory burst that increases the oxygen consumption rates (OCR) [25]. However, the roles of NAMPT-dependent respiration in the responses of melanoma cells to IFN-γ are currently unknown.

**Fig. 6:**
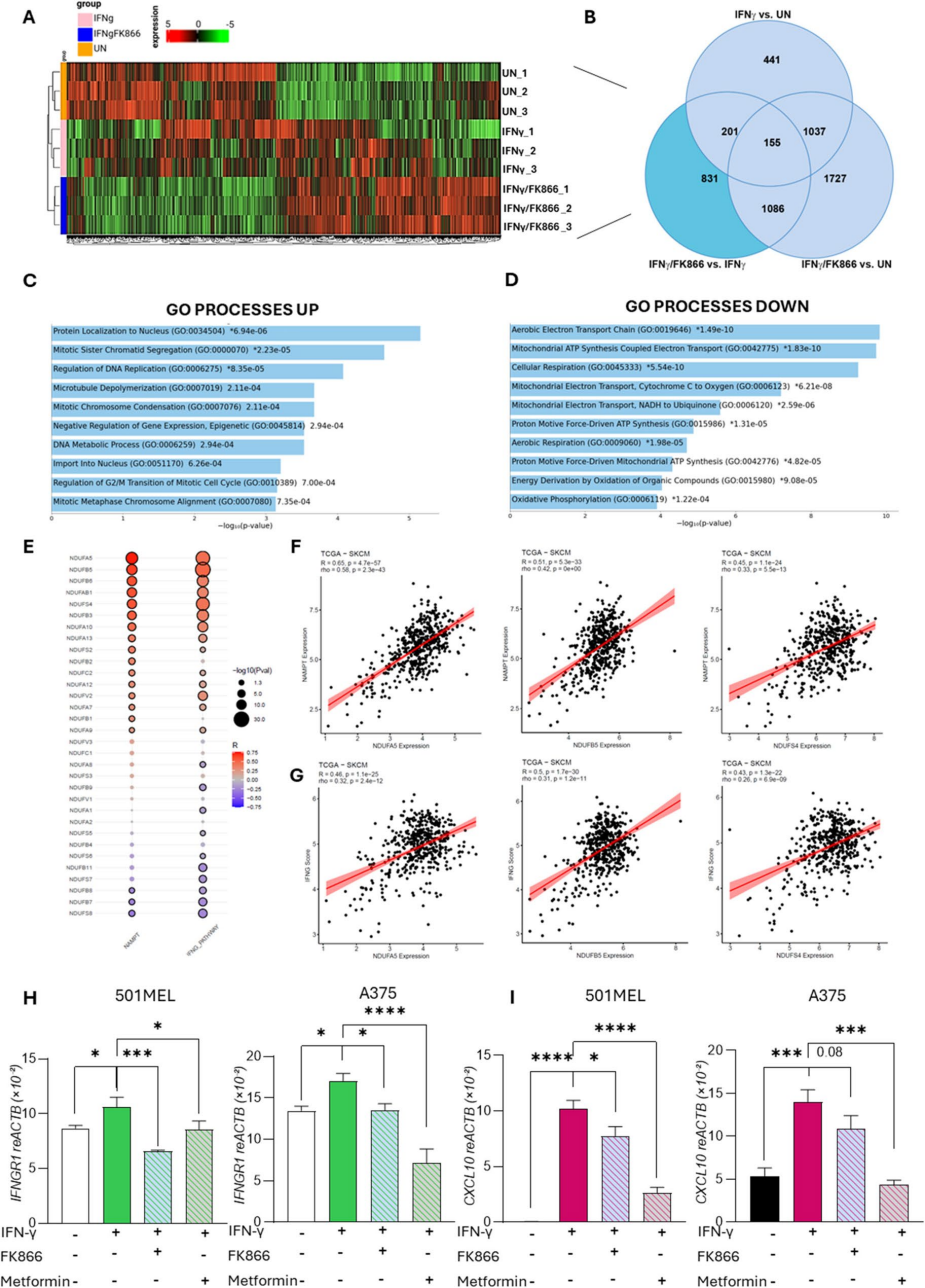
IFN-γ signaling and NAD/NAMPT axis impact on mitochondrial complex I.** A** Unsupervised heatmap showing differential gene expression, derived from RNAseq data, between 501MEL treated or not (untreated, UN) with IFN-γ (100 ng/ml) alone or in combination with FK866 (25 nM, IFN-γ/FK866) for 24 h. Red and green shadings represent higher and lower relative expression levels, respectively. Biological triplicates are shown. **B** Venn diagram depicting common and unique genes detected by RNAseq analysis in 501MEL cells. The Venn part highlighted in blue represents the unique gene signature (831 genes) of IFN-γ/FK866 condition. **C-D** Bar charts of top 10 enriched terms (GO_Biological_Process_2025 gene set library) up-regulated (**C**) or downregulated (**D**) among the 831 uniquely differentially expressed in IFN-γ/FK866 condition. The -log10(p-value) with the actual p-value are shown next to each term. Analysis was performed using EnrichR tool. **E** Heatmap-scatter plot correlating *NAMPT* expression or IFNG pathway with MCI genes in TCGA Skin Cutaneous Melanoma SKCM cohort (*n* = 471). Pearson (R) correlation and –log10(*p*-value) are shown. **F-G** Scatter plots correlating *NAMPT* expression (**F**) or IFNG score (**G**) with MCI genes including *NDUFA5*, *NDUFB5*, *NDUFS4*, respectively. Each dot represents a sample of the TCGA SKCM cohort. Pearson (R) and Spearman (rho) correlations and p-value are shown. **H-I** Histograms reporting *IFNGR1* (**H**) or *CXCL10* (**I**) mRNA expression levels in 501MEL and A375 treated with IFN-γ (100 ng/ml; 24 h) alone or in combination with FK866 (25 nM) or with Metformin (5 mM). Relative expression (*target gene/ACTB*) values were divided by 100 (×10⁻²) for a better visualization in the graphs. One-way ANOVA, multiple comparisons over IFN- γ condition was used to assess statistical significance (*p ≤ 0.05, **p ≤ 0.01, ***p ≤ 0.001, ****p ≤ 0.0001). Bar graphs show mean ± SEM (≥ 4 replicates)

Unsupervised clustering of the differentially expressed genes emerged by RNAseq analysis of the three conditions (UN, IFN-γ and IFN-γ/FK866, Fig. 6A) clearly indicated that the combination treatment up- or down-regulated blocks of selective genes compared to the other conditions. Venn diagrams of Fig. 6B illustrates the strategy chosen to identify the IFN-γ/FK866 vs. IFN-γ signature. Focusing on the 831 differentially expressed genes resulting from the comparison IFN-γ/FK866 vs. IFN-γ alone, 388 genes turned out to be upregulated while 373 were downregulated while the remaining 70 genes showed minimal changes (− 0.1 < log2FC < 0.1) and were therefore not considered (Supplementary materials). Gene ontologies enrichment analysis reported among the up-modulated genes revealed a gene signature linked with increased intracellular transport to the nucleus (Fig. 6C and Supplementary materials). This process could be related with the creation of a gradient of increased cellular stress driven by a metabolic dysfunction, that is known to deregulate nucleocytoplasmic traffic modulating importins expression and translocation of different proteins [62–65] Of note, overrepresented GO categories emerged from the analysis of downregulated genes were energetic metabolism pathways specifically related to disruption of mitochondrial respiration (Fig. 6D and Supplementary materials), in line with the key role of NAD redox factor in cellular respiration and in MCI activity [18, 19].

To explore the connections between IFN-γ signaling, NAMPT and MCI genes in melanoma patients, we conducted a systematic quantification of the association between *NAMPT* expression, IFN-γ pathway and the expression of individual genes encoding for the MCI subunit in primary and metastatic samples of human skin cutaneous melanoma from TCGA (TCGA-SKCM). 19 out of 32 MCI genes (59%) were found to be significantly correlated with *NAMPT* (Pearson correlation coefficient nominal pvalue < 0.05). Most of these genes, 16/19 (84%), were positively correlated with *NAMPT* (Fig. 6E). We further validated these associations in skin cutaneous melanoma cell lines found in the cancer cell line encyclopedia (CCLE-SKCM). Positive correlations between *NAMPT* and various MCI genes were confirmed across the 57 CCLE-SKCM cell lines (Supplementary Fig. 10A). This positive association with most of MCI genes and NAMPT reflect the key role of NAD homeostasis within the mitochondria [19].

Next, to further assess the crosstalk between *NAMPT* expression, metabolic activation and immune regulation, we quantified the correlation between the 32 MCI genes with genes belonging to the IFN-γ pathway. Interestingly, 14/16 genes (87.5%) positively associated with *NAMPT* were found also positively associated with the overall set of IFN-γ genes (Fig. 6E). *NDUFA5*, belonging to the *NADH: ubiquinone oxidoreductase subunit A5*, was the most significantly correlated gene with *NAMPT* (R 0.65) and with IFN-γ score (R 0.46) as depicted in Fig. 6E-G. *NDUFB5* is a second MCI gene positively associated with both *NAMPT* and IFN-γ score (Fig. 6E-G) but no data are available of its previous role related with this NAD metabolism and IFN-γ signaling. *NDUFS4* was also found to be highly correlated with *NAMPT* (R 0.45) and IFN score (R 0.43) as reported in Fig. 6E-G.

To experimentally validate the contribution of MCI in IFN signaling in our cellular models, we exposed 501MEL and A375 cells to IFN, IFN-γ/FK866, or IFN-γ plus Metformin, a well-known MCI inhibitor [66, 67], under the same conditions used for RNA-seq analysis. Results confirmed that interfering pharmacologically with MCI activity/ oxidative phosphorylation (OXPHOS) lead to the downregulation of IFN-γ signaling activation, negatively modulating the expression of IFN-γ receptor (Fig. 6H) and the downstream target *CXCL10* (Fig. 6I). Under these conditions, metformin was more efficient than FK866 in its capacity to dampen *CXCL10* mRNA levels (Fig. 6I).

**Fig. 7:**
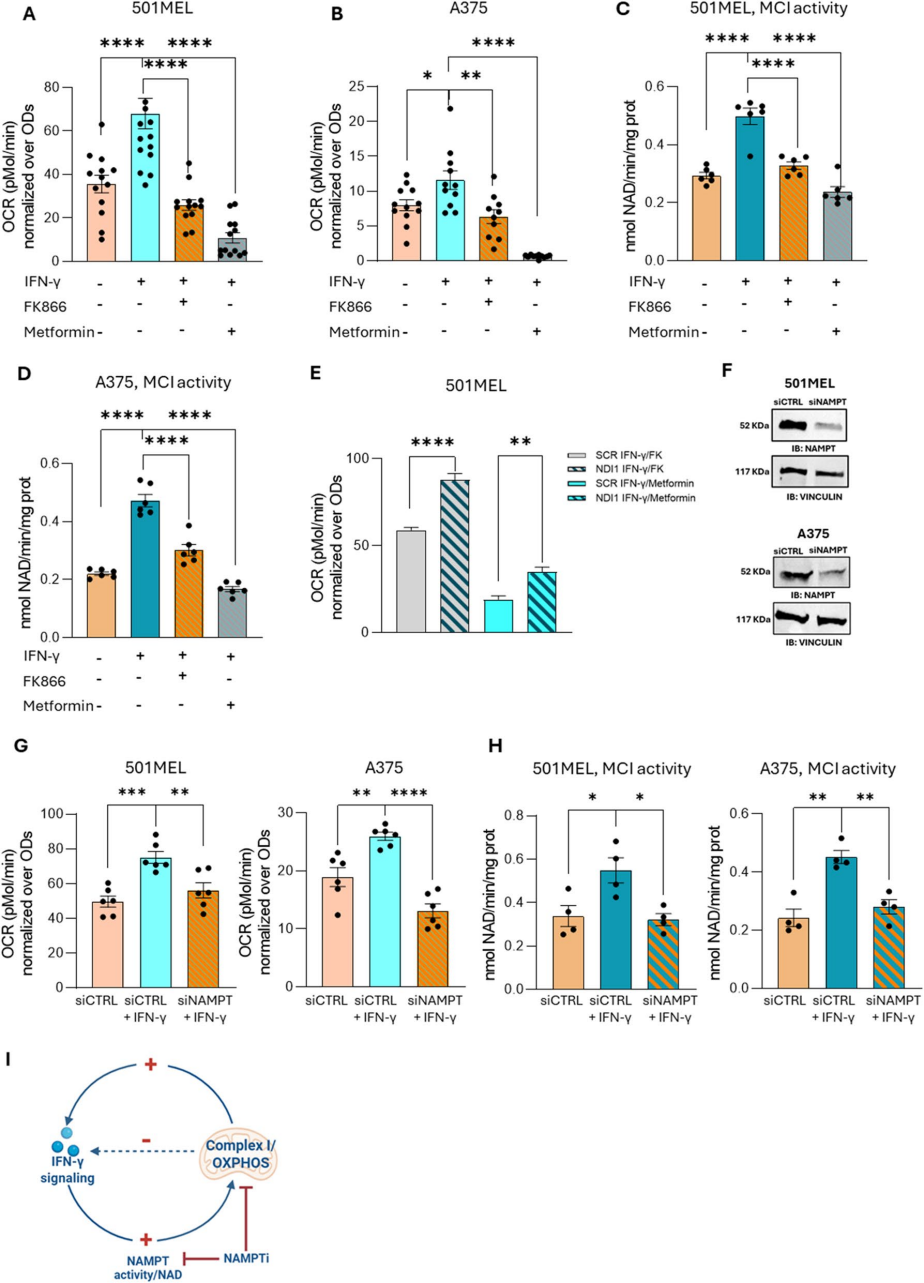
IFN-γ-induced oxygen consumption is reverted by NAD/NAMPT-dependent inhibition of complex I activity. **A**-**B** Histograms report cumulative data of oxygen consumption rate (OCR, pMol/min), measured using Resipher, measured after 24 h of treatment: in basal/untreated UN condition, after IFN-γ (100 ng/ml); IFN-γ/+FK866 (25 nM), and IFN-γ/+Metformin (5 mM). OCR data were normalized over optical density (ODs) for each sample [*n* = 12 501MEL (**A**); *n* = 7 A375 (**B**)]. **C**-**D** Histograms showing the Complex I activity (nmol cyt/min/mg protein) in 501MEL (**C**) and A375 (**D**) cells. Data from 3 independent experiments for each line, 2 technical replicates. One-way ANOVA, multiple comparisons vs. IFN-γ condition, was used to assess statistical significance (*p ≤ 0.05, **p ≤ 0.01, ***p ≤ 0.001, ****p ≤ 0.0001). **E** Histogram reporting cumulative data of OCR normalized over ODs in 501MEL (control) SCR or NDI1 transfected cells (48 h) treated for the last 24 h with IFN-γ/+FK866 (25 nM) or IFN-γ/+Metformin (5 mM). Unpaired T-test was used to calculate statistical significance (*p ≤ 0.05, ** *p* ≤ 0.01, *** *p* ≤ 0.001; ****p ≤ 0.0001). Data from 3 independent experiments, 2 technical replicates. **F** Representative western blot analysis to confirm NAMPT protein genetic silencing (48 h) using siNAMPT compared to siCTRL in 501MEL and A375 cell lines. **G** Histograms report cumulative data of OCR measured in 501MEL and A375 cells silenced for NAMPT for 48 h and tretated in the last 24 h with IFN-γ (100 ng/ml) (siNAMPT/+ IFN-γ). Control cells transfected (48 h) with a siCTRL and left untreated or treated (siCTRL/+IFN-γ) in the last 24 h with IFN-γ (100 ng/ml). OCR data were normalized over optical density (ODs) for each sample. Data from 3 independent experiments for each line, 2 technical replicates. **H** Histograms showing the Complex I activity (nmol cyt/min/mg protein) in 501MEL and A375 cells in the same condition reported in G. Data from 2 independent experiments for each line, 2 technical replicates. One-way ANOVA, multiple comparisons vs. siNAMPT/+IFN-γ condition, was used to assess statistical significance (*p ≤ 0.05, **p ≤ 0.01, ***p ≤ 0.001, ****p ≤ 0.0001). For all graphs, data are shown as mean ± SEM. **I** Cartoon of the bi-directional regulation between Mitochondrial Complex I/ETC activity and IFN-γ signaling via NAD/NAMPT axis. Figure made with BioRender.com

Lastly, we evaluated the impact of IFN-γ treatment in combination with FK866 or Metformin on OCR and MCI activity in 501MEL and A375 cells. OCR was monitored using Resipher device, which allows continuous measurement of OCR over several days [68]. We observed, in both cell lines, that IFN-γ led to an increase of OCR measured for 24 h after the starting of treatments (Fig. 7A, B and Supplementary Fig. 11A), and this effect was more significant in 501MEL cells, that also exhibit a higher basal OCR (Supplementary Fig. 11B). The IFN-γ-dependent increase of OCR was drastically reverted in the presence of FK866 or Metformin (Fig. 7A, B and Supplementary Fig. 11A). Consistently, also the global mitochondrial ETC activity followed the same trend (Supplementary Fig. 11C), likely due to an impairment of mitochondrial OXPHOS subsequent to the MCI block. Finally, to verify the effect of IFN-γ alone or in combination with FK866/Metformin on MCI, we directly measured its activity under our conditions. As expected, the data were in line with those observed for OCR and ETC activity; MCI activity increased in response to IFN-γ, and this increase was robustly blunted by either NAMPT inhibition or metformin treatment, as shown in Fig. 7C, D.

To further corroborate these results, we took advantage of the properties of the yeast NADH dehydrogenase enzyme NDI1, which is capable of rescuing the loss MCI/ETC functions when exogenously expressed in mammalian cells and is insensitive to Metformin [42, 67]. Our data indicate that in 501MEL cells transfected with a NDI1-expression construct (Supplementary Fig. 11D), the inhibitory activity of both FK866 and Metformin’s on IFN-γ- induced OCR was significantly reduced (Fig. 7E). To determine the impact of NAMPT genetic manipulation on IFN-γ-mediated metabolic activation we transiently silenced NAMPT for 48 h using a specific siRNA (Fig. 7F) and evaluated the OCR (Fig. 7G), MCI activity (Fig. 7H) and ETC efficiency (Supplementary Fig. 11E). Consistent with previous data, these results showed a significant impairment of these metabolic parameters.

Overall, these data reinforce the crucial role of MCI-dependent regeneration of NAD as a regulator of IFN-γ-signaling and IFN-γ-mediated gene expression in melanoma cells, as summarized in the cartoon diagram of Fig. 7I.

### NAMPT inhibition in melanoma cells antagonizes IFN-γ–induced immunomodulatory functions

**Fig. 8:**
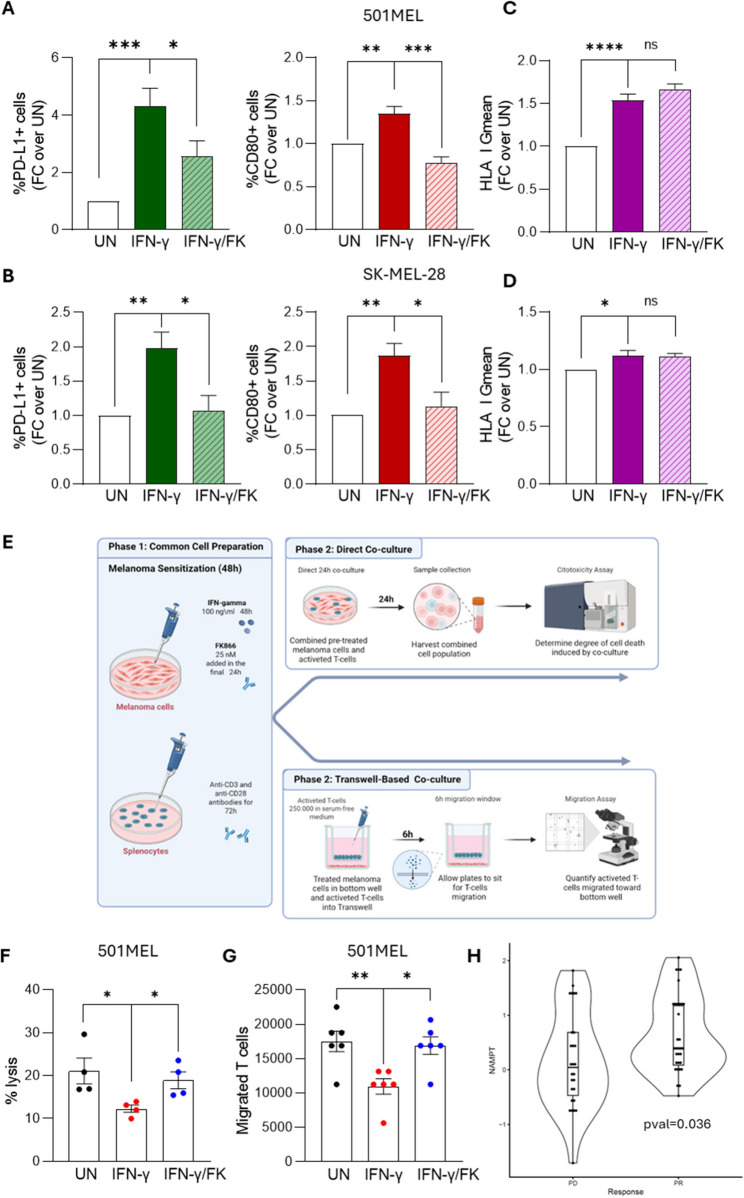
NAMPT inhibition in melanoma cells antagonizes IFN-γ–induced immunomodulatory functions.** A-B** Histograms showing the percentage of PD-L1-positive cells and CD80-positive cells (501MEL, **A** and SK-MEL-28, **B**) measeured using flow cytometry. Cells were treated with IFN-γ (100 ng/ml) alone or combined with FK866 (25 nM) for 24 h. Data are reported as fold change (FC) compared untreated condition. **C-D** Histograms showing the HLA I Gmean values detected by FACS analysis in 501MEL, (**C**) and SK-MEL-28 (**D**) cells treated as described in (A-B). Data are reported as fold change (FC) compared untreated condition. Bar graphs show mean ± SEM (*n* ≥ 4 replicates for A-D). One-way ANOVA, multiple comparisons over IFN-γ: *p ≤ 0.05, **p ≤ 0.01, ***p ≤ 0.001. **E** Schematic workflow of co-culture models used to analyze the interaction between melanoma cells and activated T-cells. Melanoma cells and mice-derived splenocytes were pre-treated as indicated and then direct or transwell medited co-culture was performed to asses T cell mediated tumor cytotoxicity and T-cell migration. Image created in BioRender. Audrito, V. (2026) https://BioRender.com/ydm514k. **F** Histogram showing the percentage of lysis of 501MEL cells treated or not for 48 h with IFN-γ (100 ng/ml) alone or combined in the last 24 h with FK866 (FK, 25 nM) and then co-cultured for 24 h with activated T cells, evaluated by FACS analysis (tumor cell death was determined as the percentage of 7-AAD⁺ cells and calculated as reported in Materials and Methods). Bar graphs show mean ± SEM (*n* = 2 biological replicates with 2 technical replicates). **G** Histogram showing the number of migrated activated T cells in transwell system attracted by 501MEL cells treated as indicated in F. Bar graphs show mean ± SEM (*n* = 3 biological replicates). One-way ANOVA, multiple comparisons over IFN-γ: *p ≤ 0.05, **p ≤ 0.01. **H** Violin plots reporting the association between *NAMPT* expression levels and anti-PD1 treatment outcome (75 MCM patients) included in this study [73]: patients that experienced partial response (PR) to anti-PD1 have significantly higher *NAMPT* transcript respect to those that didn’t respond to the treatment (progressive disease, PD). Pairwise Wilcoxon Rank Sum test was used to define pval

It is known that IFN-γ exposure induces a state of adaptive immune resistance in melanoma cells that negatively impacts both T cell recruitment and effector function [1]. To evaluate how NAMPT inhibition in melanoma cells affects this IFN-γ-mediated reprogramming of tumor cells toward an immunosuppressive phenotype, we first analyzed the expression of costimulatory and immune checkpoint molecules at melanoma cell surface. Results showed that FK866 treatment caused a significant decrease of IFN-γ-inducible PD-L1, CD80, and CD86 surface protein expression, as revealed by FACS analysis of 501MEL and SK-MEL-28 (Fig. 8A, B and Supplementary Fig. 12A). In A375 cells the expression of these molecules was barely detectable, and this prevented their quantitative assessment (data not shown). On the contrary, the expression of HLA-I antigens, crucial mediators of antigen presentation to the immune cells, was unaffected by NAMPT inhibition (Fig. 8C, D). To evaluate at the functional levels how NAMPT manipulation affects the interplay between melanoma cells and activated T effector cells, we established an experimental model consisting of a co-culture of pre-activated T cell-enriched splenocytes derived from C57BL/6 HLA-A2.1 transgenic mice and matched human HLA-A2^+^ 501MEL cells. Melanoma cells were either kept untreated or treated with IFN-γ for 48 h or IFN-γ plus FK866. The killing of 501MEL by activated T cells was then measured by FACS analysis (see workflow in Fig. 8E). Consistent with previous reports [69], IFN-γ-pre-treated 501MEL were significantly protected from T-mediated cytotoxicity. Importantly, NAMPT inhibition reverted this effect (Fig. 8F). Lastly, in a similar experimental setting (workflow in Fig. 8E) we also measured how NAMPT inhibition in tumor cells modulates T cell migration through transwell inserts. Results showed that 501MEL cells pre-treated with IFN-γ reduced their capacity to attract activated T cells (Fig. 8G). NAMPT inhibition in tumor cells restored this effect to levels closed to those of untreated control cells (Fig. 8G). Within these in vitro contexts, these data indicate that manipulation of NAMPT signaling in tumor cells can influence tumor-immune interactions, suggesting that NAMPT inhibition in melanoma cells may also attenuate at least some immunosuppressive effects induced by IFN-γ. However, similarly to IFN-γ-induced PD-L1 expression [70–72], also IFN-γ-induced NAMPT may promote immune evasion on one hand, while on the other hand may render tumors more responsive to ICIs. To explore this possibility in a clinical setting we have analyzed a cohort of 75 melanoma patients treated with anti-PD1 published in [73] that revealed a statistically significant association between treatment outcome and NAMPT expression levels. In particular, patients that experienced partial response (PR) to anti-PD1 have significantly higher *NAMPT* transcript respect to those that did not respond to the treatment (progressive disease, PD) as reported in Fig. 8H. Moreover, lower NAMPT expression also correlates with an overall worse prognosis in the cohort of patients treated with anti-PD1 (R 0.51, pval 0.013).

## Discussion

Our study identifies a reciprocal regulatory circuit between IFN-γ signaling and the NAD-biosynthetic enzyme NAMPT, providing new insight into the metabolic control of cytokine responses in melanoma cells. We demonstrate that IFN-γ transcriptionally induces NAMPT expression through a coordinated mechanism involving STAT1, IRF1, and the epigenetic reader BRD4. In turn, NAMPT activity is required to sustain IFN-γ signaling output, as its inhibition impairs STAT1 activation and the expression of IFN-γ-responsive genes. Mechanistically, this effect is linked to NAD-dependent regulation of mitochondrial complex I (MCI) activity and oxidative metabolism. Together, these findings position NAMPT as a functional component of the IFN-γ response network rather than a mere downstream target, defining a novel immuno-metabolic axis that integrates cytokine signaling, chromatin remodeling, and redox metabolism in MCM cells.

The identification of NAMPT as an IFN-γ-inducible gene in melanoma extends previous observations in macrophages and other tumor models [25, 26], and indicates that NAD metabolism is embedded within canonical IFN transcriptional programs. In our system, NAMPT induction, observed in human and murine melanoma cell lines independently of BRAF mutational status, occurs alongside classical IFN-γ targets such as STAT1, IRF1, CXCL10, and PD-L1, suggesting coordinated regulation of immune signaling and metabolic adaptation. Notably, we previously observed increased NAMPT expression in BRAFi-resistant melanoma, where it was primarily interpreted as a tumor-intrinsic metabolic adaptation [14, 15, 17]. The present findings suggest that, in addition to intrinsic stress responses, IFN-γ signaling may contribute to NAMPT upregulation in tumor cells exposed to an inflammatory microenvironment, thereby linking metabolic rewiring to immune-derived cues.

The clinical relevance of this axis is supported by the consistent association between NAMPT expression and IFN-γ transcriptional signatures across melanoma datasets, as well as by the positive correlation between NAMPT and PD-L1 observed at both transcriptomic and protein levels in MCM patient samples. These findings support a link between NAD metabolism and IFN-responsive tumor phenotypes. However, while PD-L1 expression has been associated with response to immune checkpoint blockade in some contexts [74, 75], our data do not establish a direct causal relationship between NAMPT levels and therapeutic outcome, and this aspect requires further investigation in appropriately designed clinical and experimental studies.

At the mechanistic level, our work uncovers a previously unrecognized role for BRD4 and IRF1 in regulating NAMPT transcription downstream of IFN-γ. While STAT1-dependent control of NAMPT has been described [26, 30], our data indicate that efficient transcriptional activation requires the coordinated recruitment of BRD4 and IRF1 to the NAMPT promoter, mirroring the regulatory architecture previously reported for PD-L1 [35]. This defines a shared transcriptional module that integrates chromatin remodeling and cytokine signaling to coordinate metabolic and immunoregulatory gene expression. Given the ongoing clinical evaluation of BET inhibitors [76], these findings suggest that modulation of BRD4 activity may impact not only oncogenic transcriptional programs but also tumor-cell IFN responsiveness and associated metabolic states.

A key finding of this study is the demonstration that NAMPT is not only induced by IFN-γ but is also required to sustain IFN-γ signaling. Pharmacological inhibition or genetic silencing of NAMPT attenuated STAT1 activation and reduced the expression of multiple IFN-γ target genes, indicating that NAMPT-dependent NAD metabolism is necessary for full pathway activation. NAD^+^/NADH is involved in OXPHOS process within the mitochondria, where NAD^+^ is reduced to NADH in the tricarboxylic acid (TCA) cycle and is subsequently oxidized to NAD^+^ in ETC specifically through MCI for ATP generation [19]. Functional and transcriptomic analyses further revealed that this effect is mediated, at least in part, through regulation of mitochondrial respiration. In particular, NAMPT inhibition impaired MCI activity and oxygen consumption, thereby limiting the metabolic support required for IFN-γ–driven transcriptional responses. These findings are consistent with previous studies in immune cells showing that IFN-γ-induced metabolic reprogramming depends on NAD regeneration to sustain mitochondrial function [23, 25].

The integration of signaling and metabolic data supports a model in which melanoma cells establish a feed-forward regulatory circuit, whereby IFN-γ induces NAMPT expression to preserve NAD homeostasis, and NAMPT activity in turn maintains mitochondrial metabolism required for sustained IFN-γ signaling (Fig. 7I). The relevance of this axis is further supported by the observed correlation between NAMPT expression, IFN-γ transcriptional signatures, and the expression of multiple mitochondrial complex I subunits (*NDUFA5*,* NDUFB5*,* NDUFS4*) [77] in melanoma datasets. Together, these observations define a coordinated NAD/NAMPT–OXPHOS–IFN-γ axis that links cytokine signaling to cellular metabolic state.

Importantly, our co-culture experiments provide initial functional evidence that modulation of NAMPT activity in melanoma cells influences tumor-T cell interactions. Although these experiments were performed using a T cell-enriched splenocyte population rather than fully purified T lymphocytes, activated T cells represented the predominant immune population under our experimental conditions. Specifically, NAMPT inhibition attenuated IFN-γ-induced changes in melanoma cells that affect T cell cytotoxicity and migration. While these assays do not recapitulate the full complexity of the tumor microenvironment, they indicate that tumor-cell intrinsic regulation of IFN-γ signaling by NAMPT can have measurable consequences on T cell behavior. The biological implications of this axis are likely to be context dependent. IFN-γ itself exerts both pro-inflammatory and immunosuppressive effects in cancer, a phenomenon often referred to as the “IFN-γ paradox” [78]. In line with this concept, NAMPT-dependent reinforcement of IFN-γ signaling may contribute to adaptive immune resistance in certain contexts, for example through induction of PD-L1 and other inhibitory pathways, while in other settings it may be associated with tumor states that are more responsive to immunotherapy. Consistent with this possibility, we observed an association between higher NAMPT expression and improved response in a cohort of patients treated with anti-PD1 therapy. However, our data do not allow us to predict the net effect of targeting NAMPT in combination with immune checkpoint blockade, and further studies in more complex models will be required to address this question.

From a translational perspective, our findings highlight NAMPT as a metabolic regulator of IFN-γ responsiveness in melanoma cells and suggest that NAD metabolism may influence tumor-immune interactions. At the same time, the dual and context-dependent nature of IFN-γ signaling underscores the need for caution in extrapolating these results to therapeutic strategies. Future studies integrating tumor-intrinsic and immune components in vivo will be essential to determine whether modulation of the NAMPT/NAD axis can be exploited to improve responses to existing therapies.

## Conclusion

In conclusion, our work identifies NAMPT as a key component of the IFN-γ signaling network in melanoma cells, linking cytokine signaling, chromatin regulation, and mitochondrial metabolism within a self-reinforcing regulatory circuit. These findings provide a conceptual framework to further investigate how metabolic control of IFN-γ signaling contributes to tumor cell behavior and tumor-immune interactions.

## Supplementary Information

Supplementary Material 1.

Supplementary Material 2.

Supplementary Material 3.

Supplementary Material 4.

Supplementary Material 5.

Supplementary Material 6.

## Data Availability

The datasets used and/or analyzed during the current study are available from the corresponding author on reasonable request. The RNA-seq data were deposited on Gene Expression Omnibus (GEO) repository with the accession number GSE310354.

## Abbreviations

BET protein: bromodomain and extra-terminal protein
BRAFi: BRAF inhibitors
BRD4: bromodomain-containing protein 4
CCLE: cancer cell line encyclopedia
CD274: cluster of differentiation 274
ChIP: chromatin immunoprecipitation
CTLA-4: cytotoxic T-lymphocyte–associated protein 4
CXCL10: C-X-C motif chemokine ligand 10
ETC: electron transport chain
eNAMPT: extracellular nicotinamide phosphoribosyltransferase
ICIs: immune checkpoint inhibitors
IDO1: indoleamine 2,3-dioxygenase 1
IFN-γ: interferon gamma
IFNGR1: interferon gamma receptor 1
iNAMPT: intracellular nicotinamide phosphoribosyltransferase
IRF1: interferon regulatory factor 1
MCI: mitochondrial complex I
MCM: metastatic cutaneous melanoma
NAD: nicotinamide adenine dinucleotide
NAMPT: nicotinamide phosphoribosyltransferase
NDI1: NADH dehydrogenase (yeast)
NDUFA5: NADH: ubiquinone oxidoreductase subunit A5
NDUFB5: NADH: ubiquinone oxidoreductase subunit B5
NDUFS4: NADH: ubiquinone oxidoreductase subunit S4
NNMT: nicotinamide N-methyltransferase
OCR: oxygen consumption rate
OXPHOS: oxidative phosphorylation
PARP: poly(ADP-ribose) polymerase
PD-1: programmed death 1
PD-L1: programmed death-ligand 1
RNA-seq: RNA sequencing
SKCM: skin cutaneous melanoma
STAT1: signal transducer and activator of transcription 1
TCGA: the cancer genome atlas
TMA: tissue microarray
TME: tumor microenvironment
TT: targeted therapy
