# Supplementary material for: Bi-directional regulation between NAD/NAMPT and IFN-γ/PD-L1 axes via BRD4/IRF1 and mitochondrial respiration in metastatic cutaneous melanoma
Source: J Exp Clin Cancer Res. 2026 May 14;45:147. doi: 10.1186/s13046-026-03734-2 (PMC13326467; doi:10.1186/s13046-026-03734-2)
Supplement: Supplementary file 4 — Supplementary Material 4. [file 13046_2026_3734_MOESM4_ESM.pdf]

FIGURE 1H

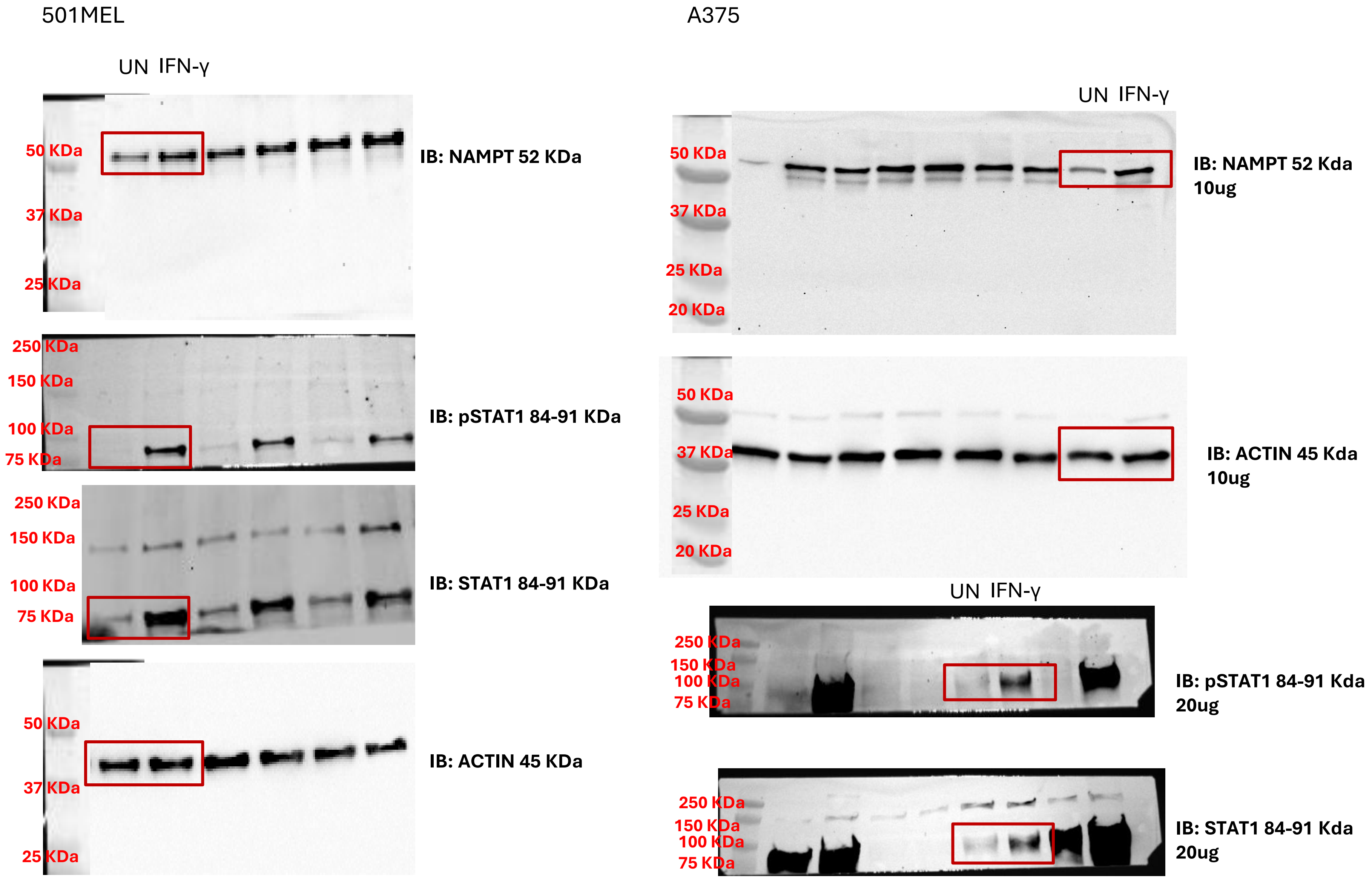

501MEL
UN IFN-γ
50 KDa
37 KDa
25 KDa
IB: NAMPT 52 KDa
250 KDa
150 KDa
100 KDa
75 KDa
IB: pSTAT1 84-91 KDa
250 KDa
150 KDa
100 KDa
75 KDa
IB: STAT1 84-91 KDa
50 KDa
37 KDa
25 KDa
IB: ACTIN 45 KDa
A375
UN IFN-γ
50 KDa
37 KDa
25 KDa
20 KDa
IB: NAMPT 52 Kda
10ug
50 KDa
37 KDa
25 KDa
20 KDa
IB: ACTIN 45 Kda
10ug
UN IFN-γ
250 KDa
150 KDa
100 KDa
75 KDa
IB: pSTAT1 84-91 Kda
20ug
250 KDa
150 KDa
100 KDa
75 KDa
IB: STAT1 84-91 Kda
20ug

FIGURE 1H

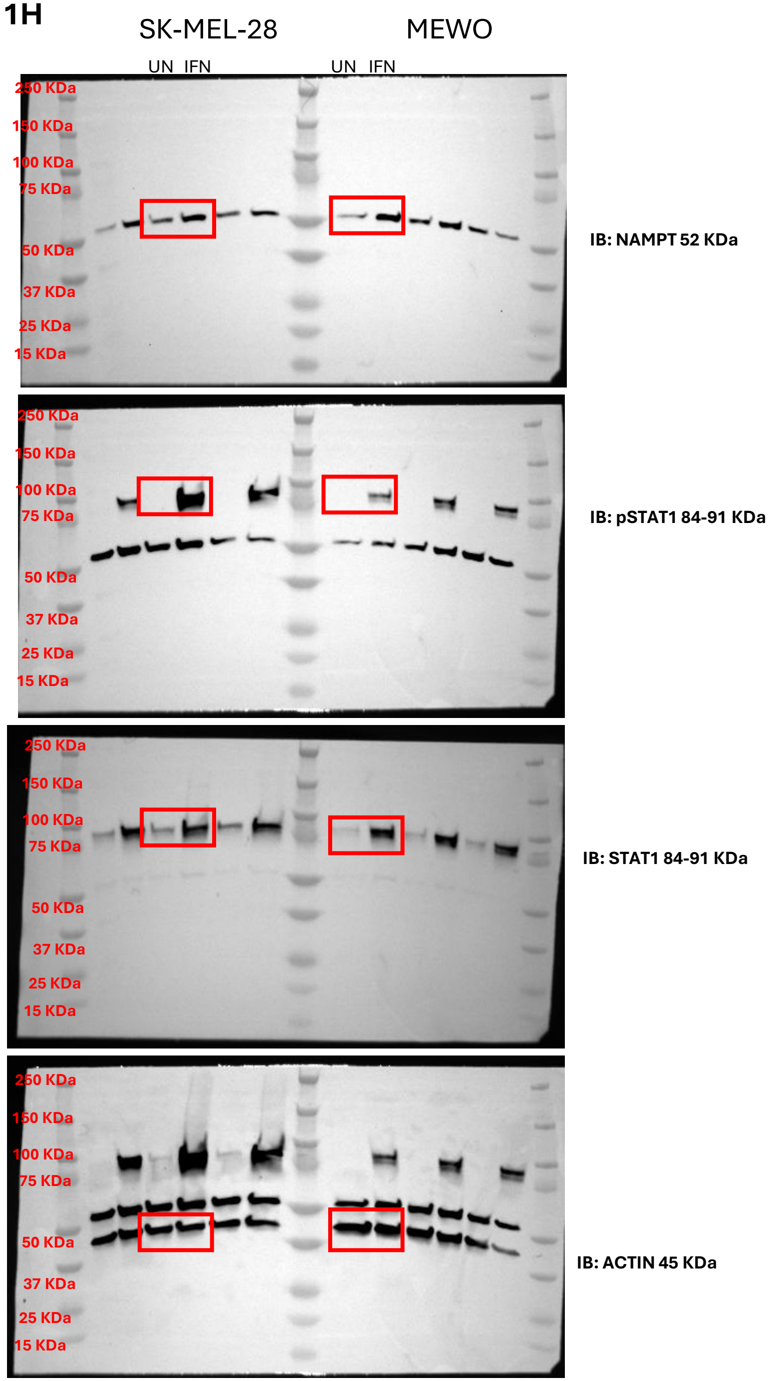

FIGURE 1I

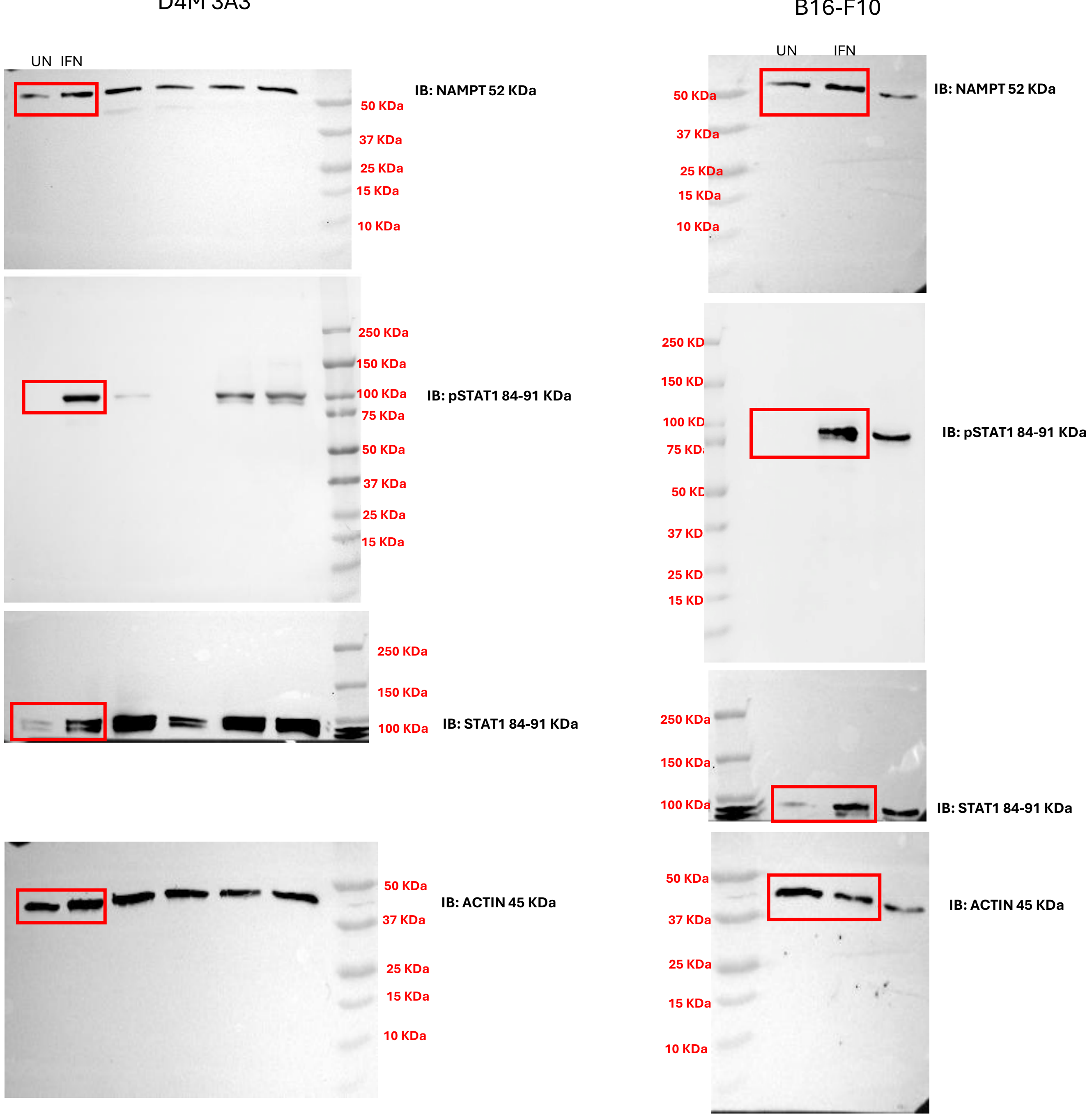

FIGURE 1K

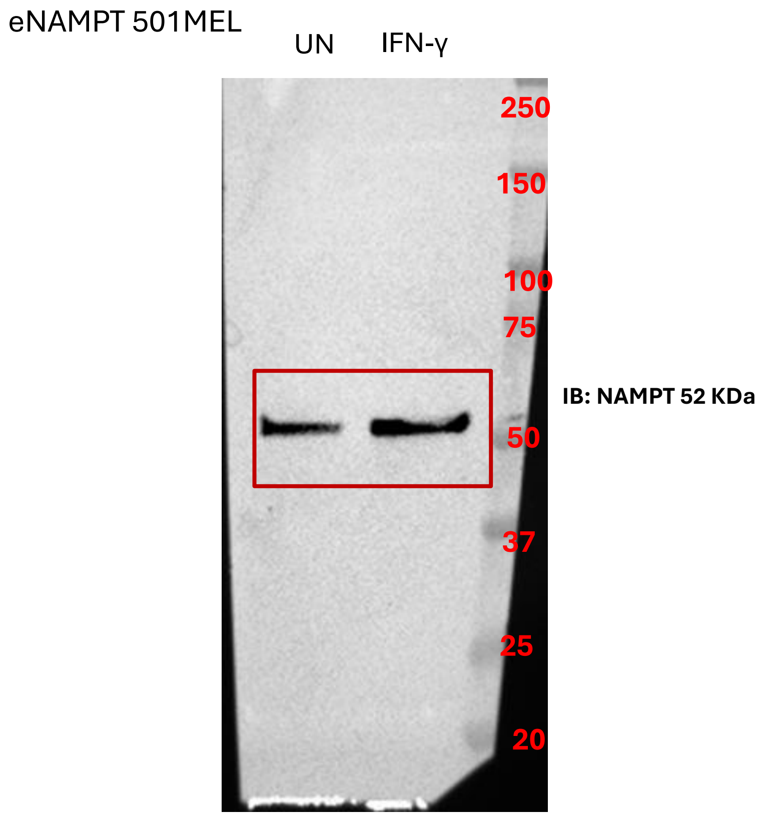

FIGURE 1L

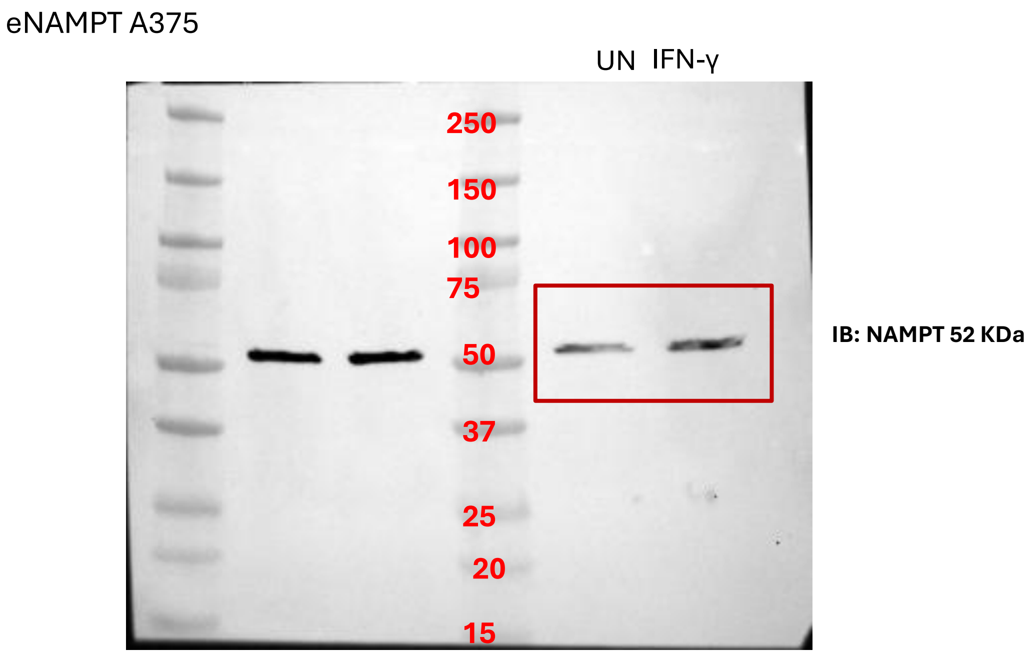

FIGURE 3B

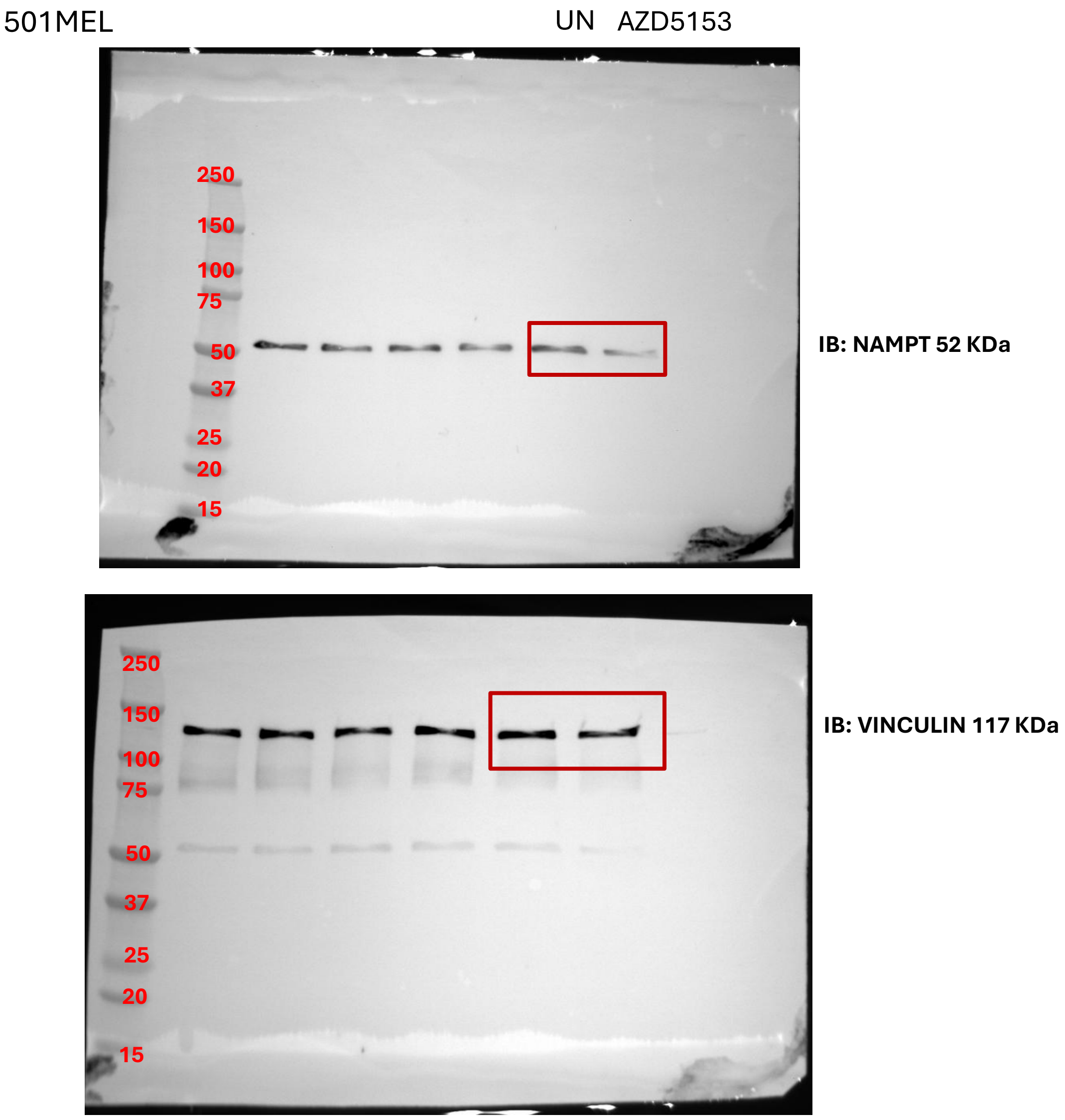

Fig. Suppl 5A

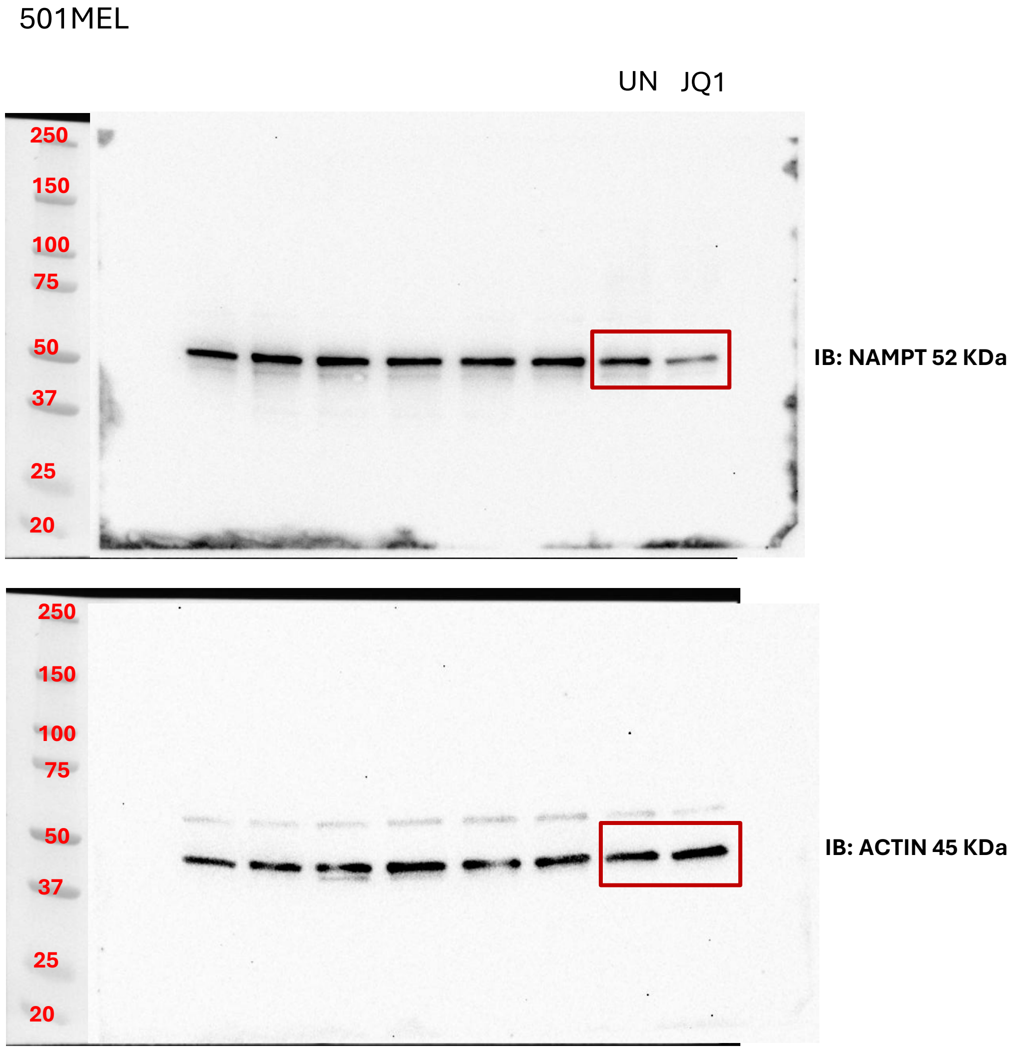

FIGURE 3B and Fig. Suppl 5A

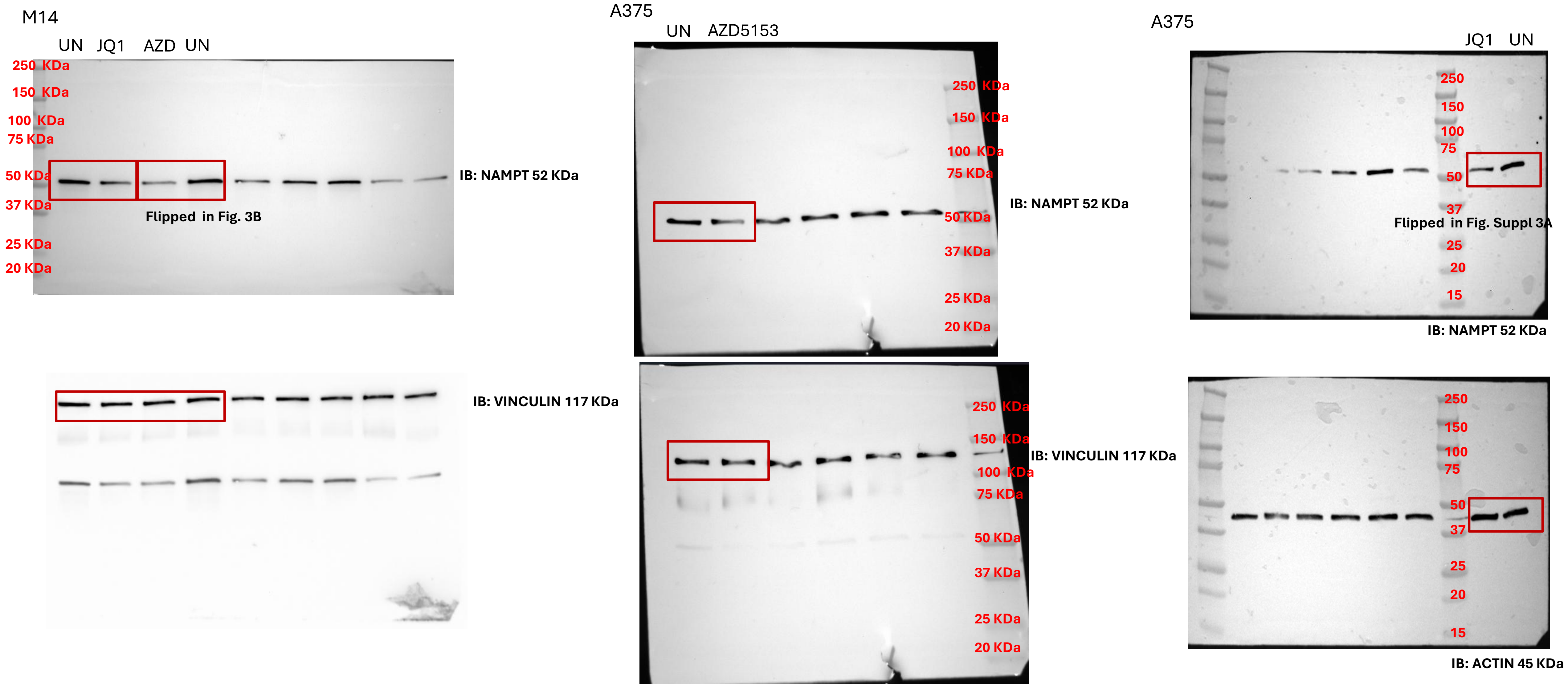

FIGURE 3E

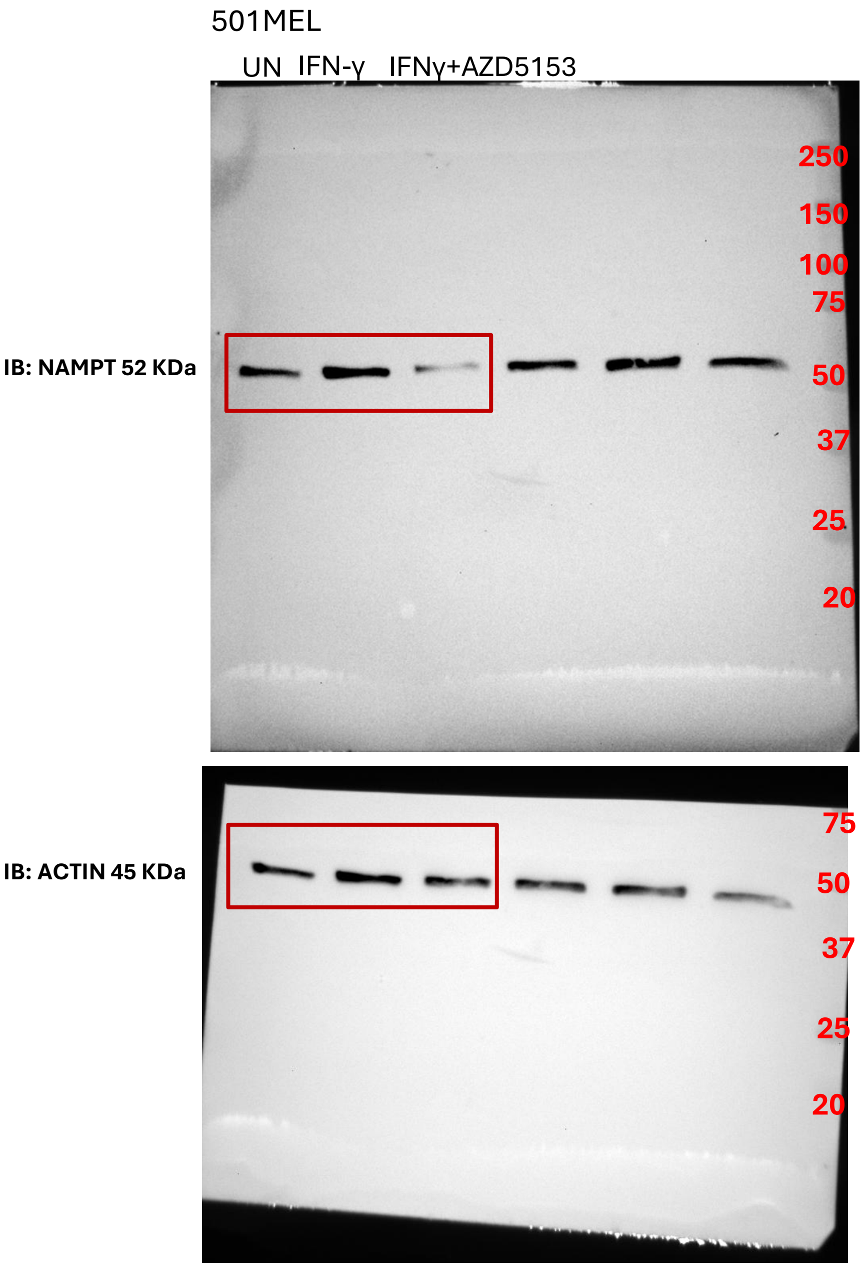

Suppl. FIGURE 5C

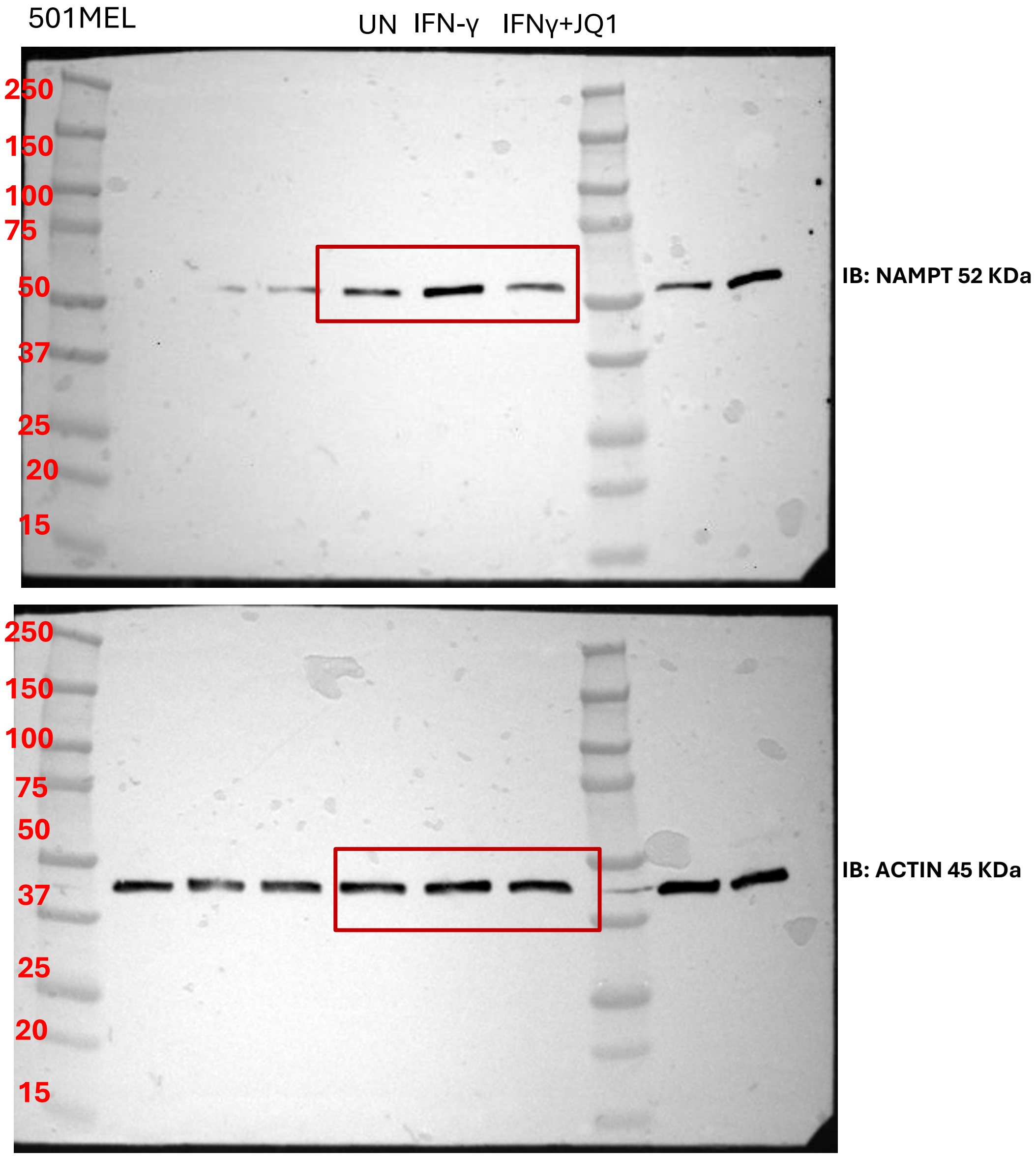

SUPPLEMENTARY FIGURE 3F

501MEL

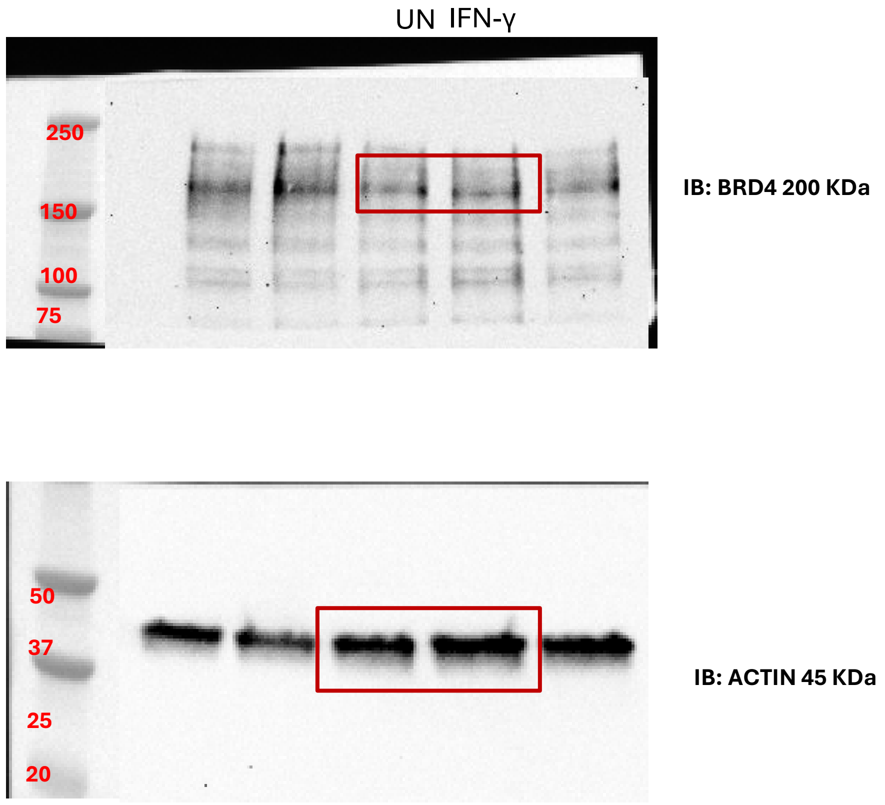

FIGURE 5A

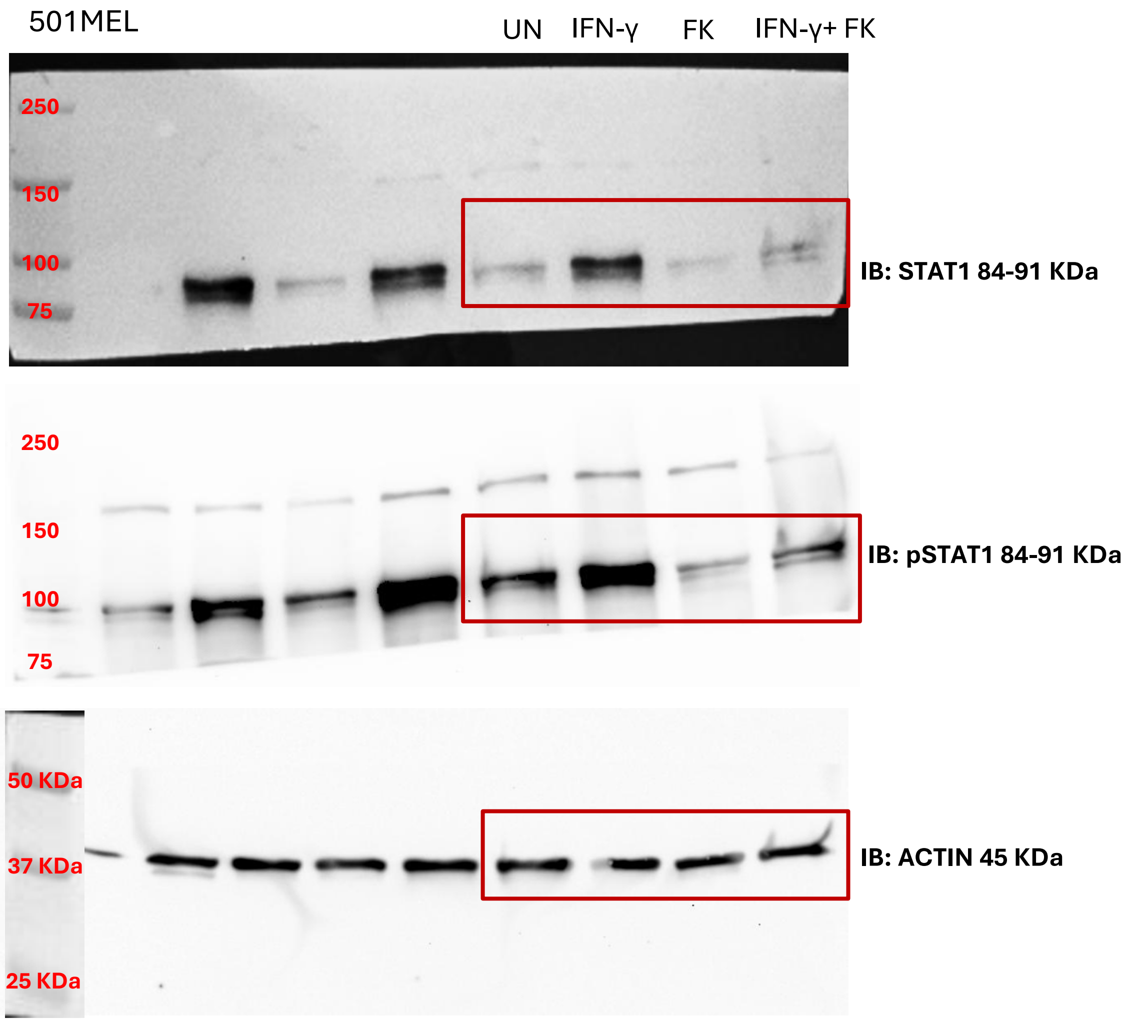

FIGURE 5B

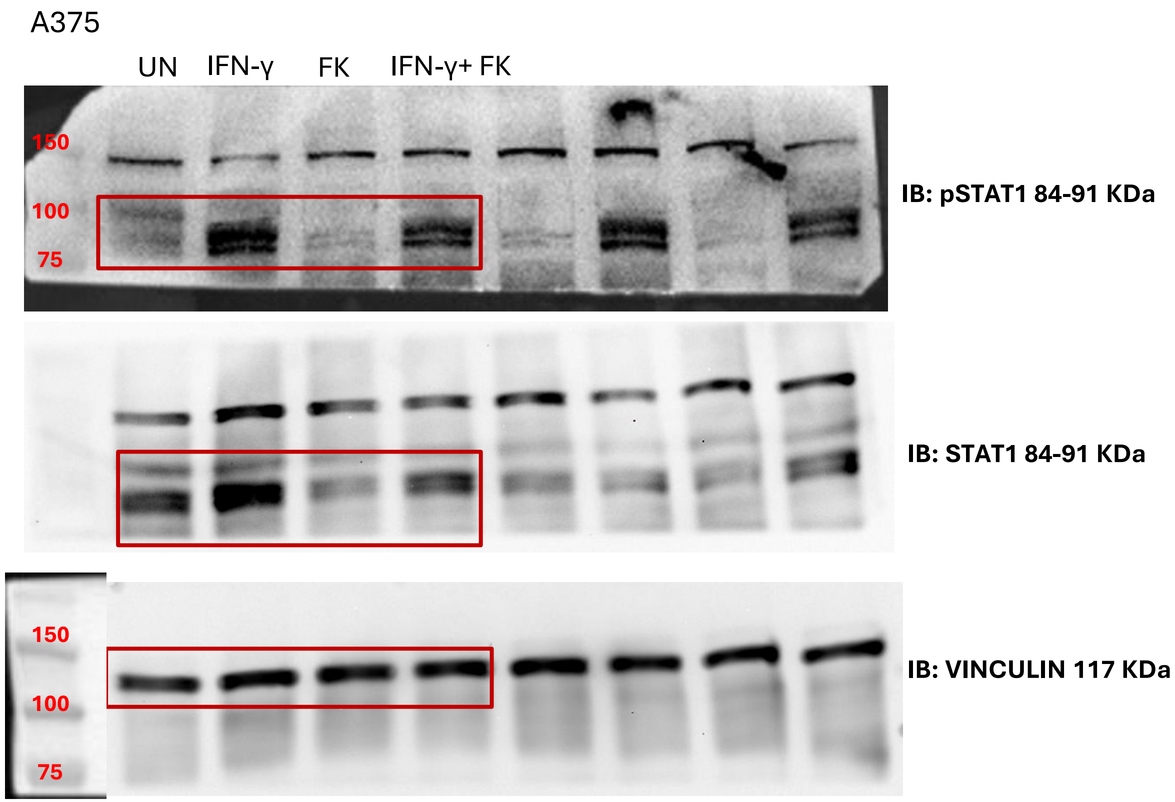

FIGURE 5C

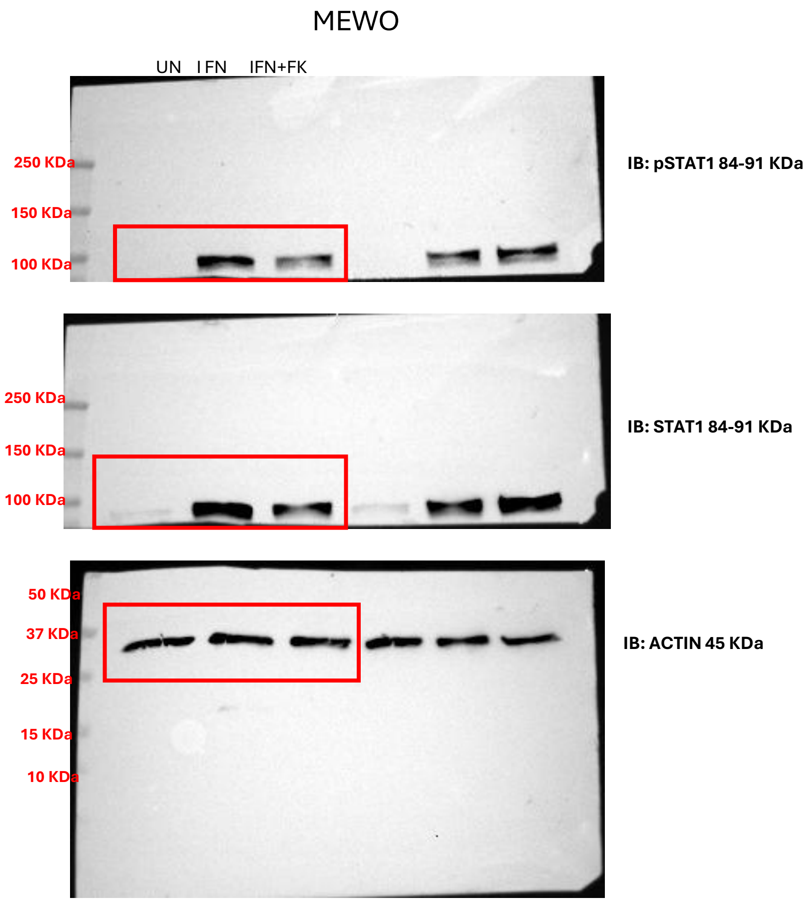

FIGURE 5D

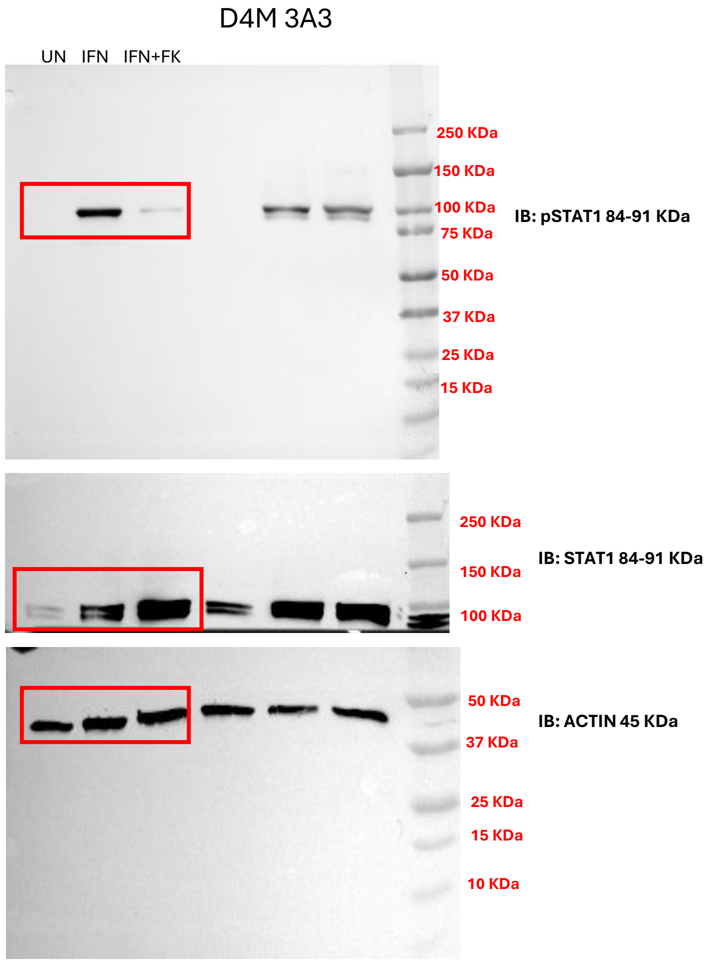

FIGURE 5E

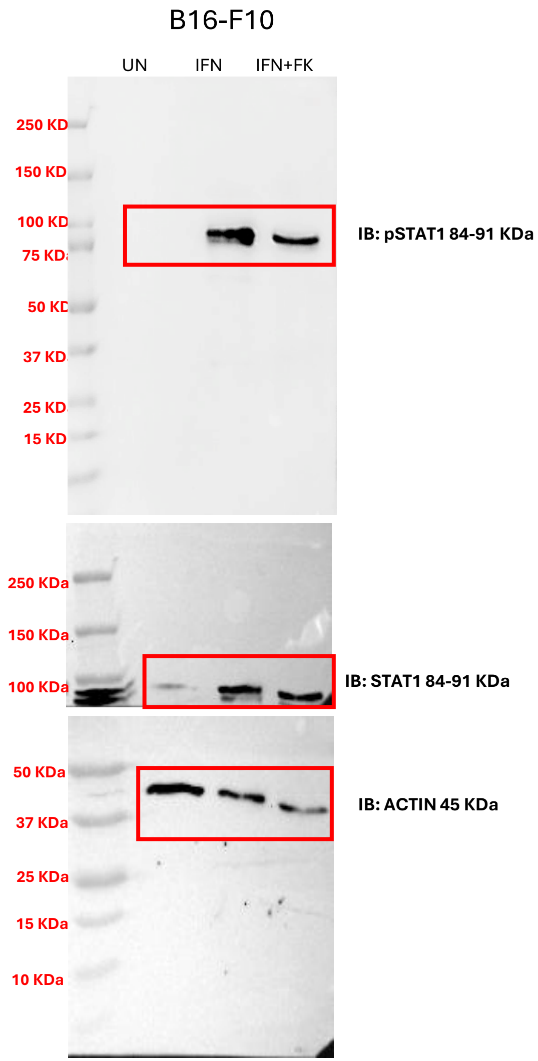

FIGURE 7F

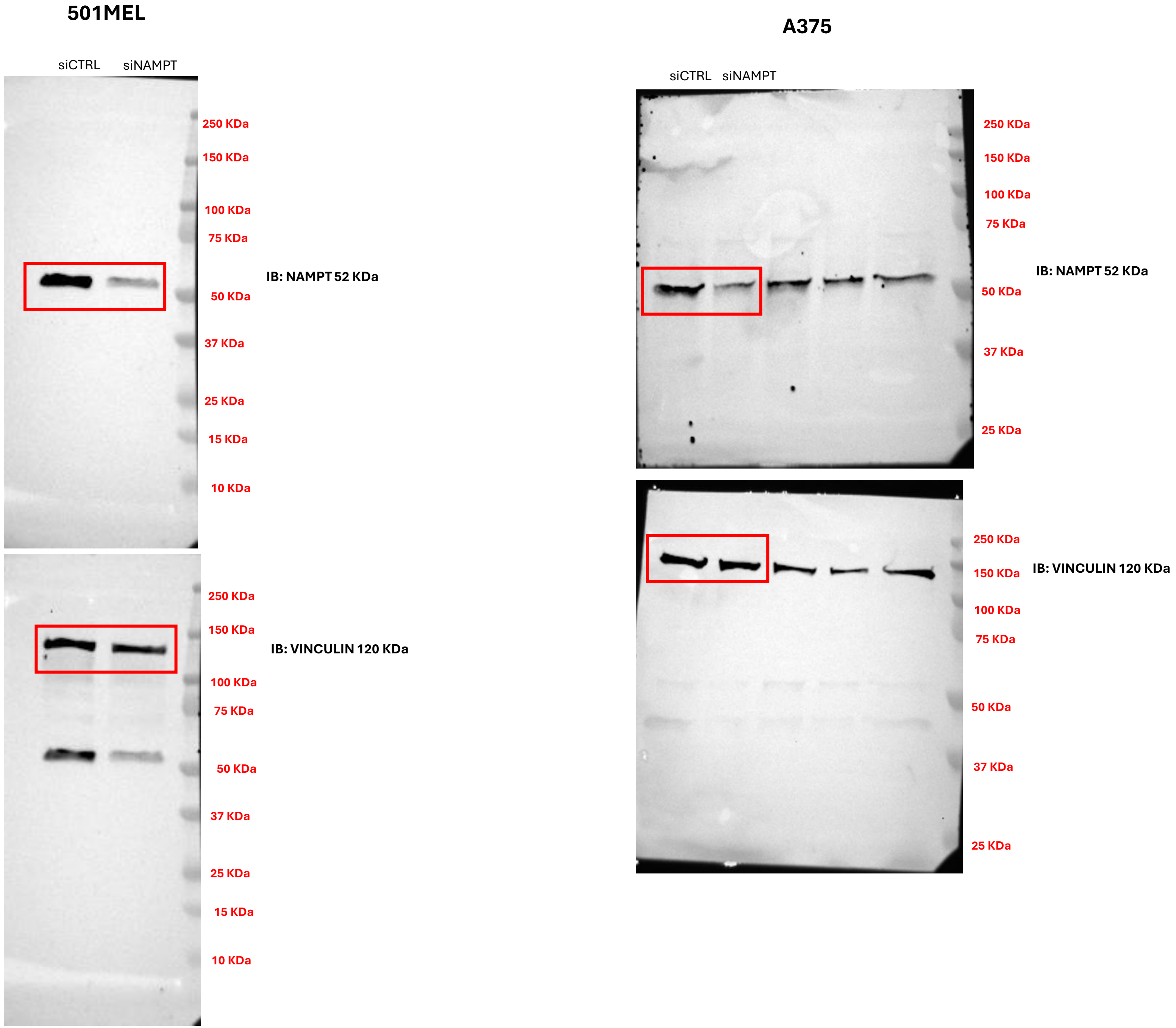
501MEL
siCTRL
siNAMPT
250 KDa
150 KDa
100 KDa
75 KDa
IB: NAMPT 52 KDa
50 KDa
37 KDa
25 KDa
15 KDa
10 KDa
250 KDa
150 KDa
IB: VINCULIN 120 KDa
100 KDa
75 KDa
50 KDa
37 KDa
25 KDa
15 KDa
10 KDa
A375
siCTRL
siNAMPT
250 KDa
150 KDa
100 KDa
75 KDa
IB: NAMPT 52 KDa
50 KDa
37 KDa
25 KDa
250 KDa
IB: VINCULIN 120 KDa
150 KDa
100 KDa
75 KDa
50 KDa
37 KDa
25 KDa

SUPPLEMENTARY FIGURE 7B

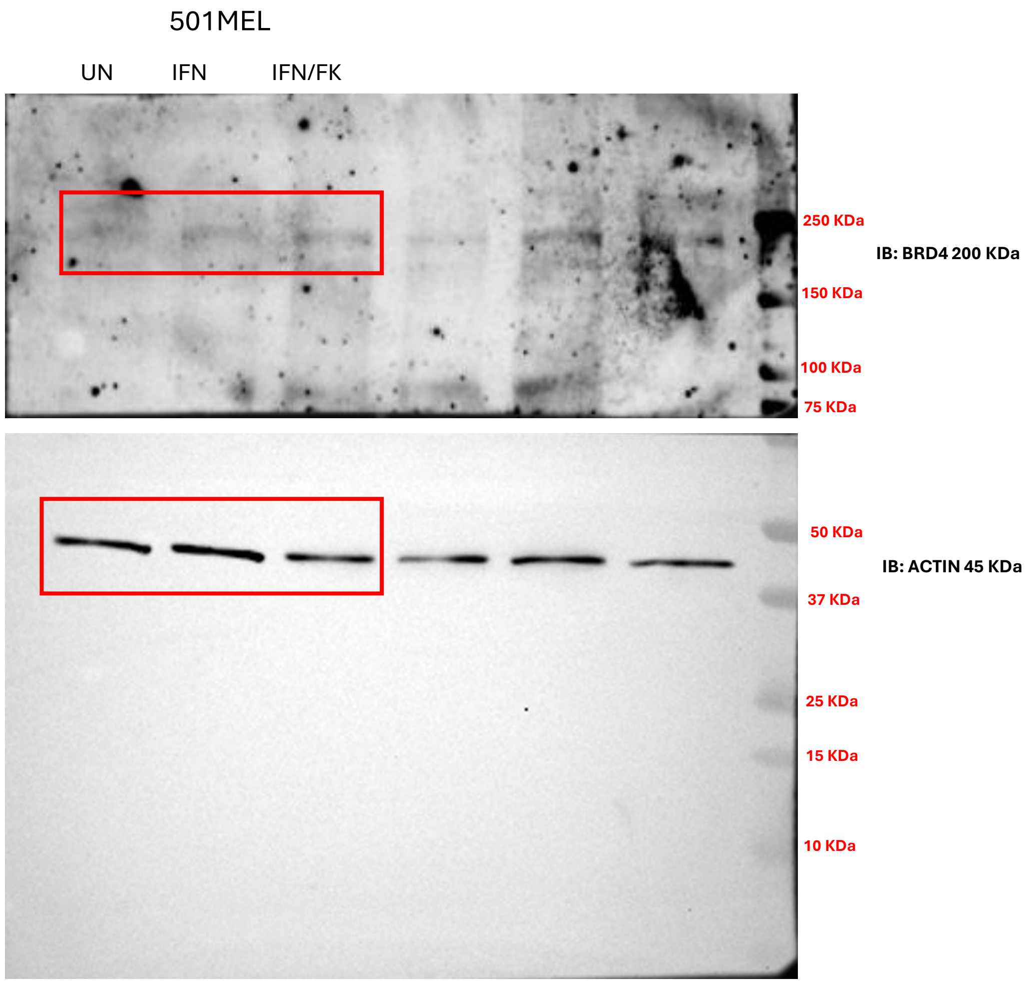

SUPPLEMENTARI FIGURE 9A

501MEL

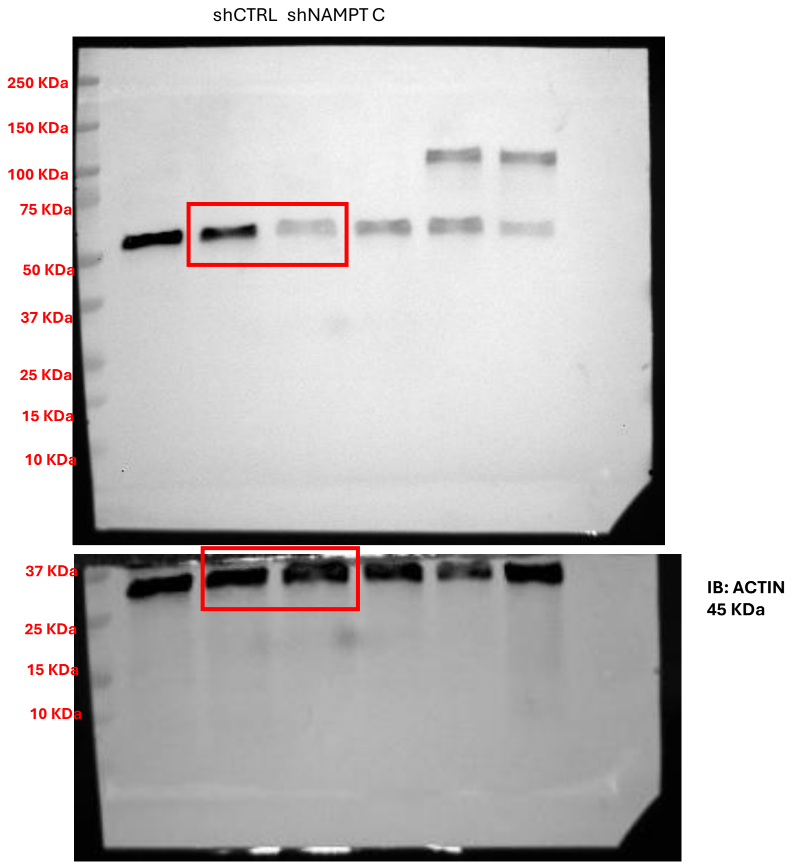

A375

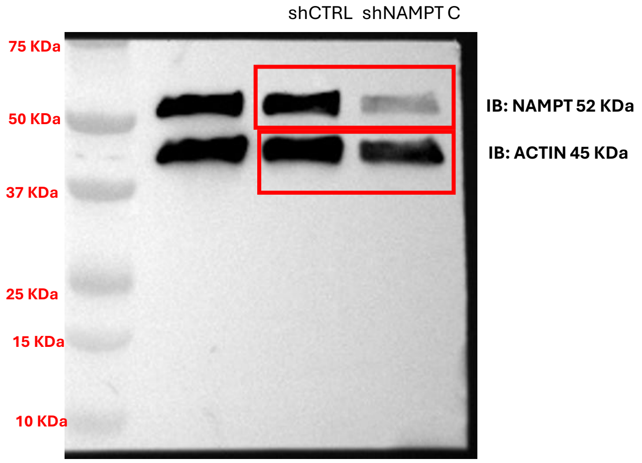

SUPPLEMENTARY FIGURE 11D

501MEL

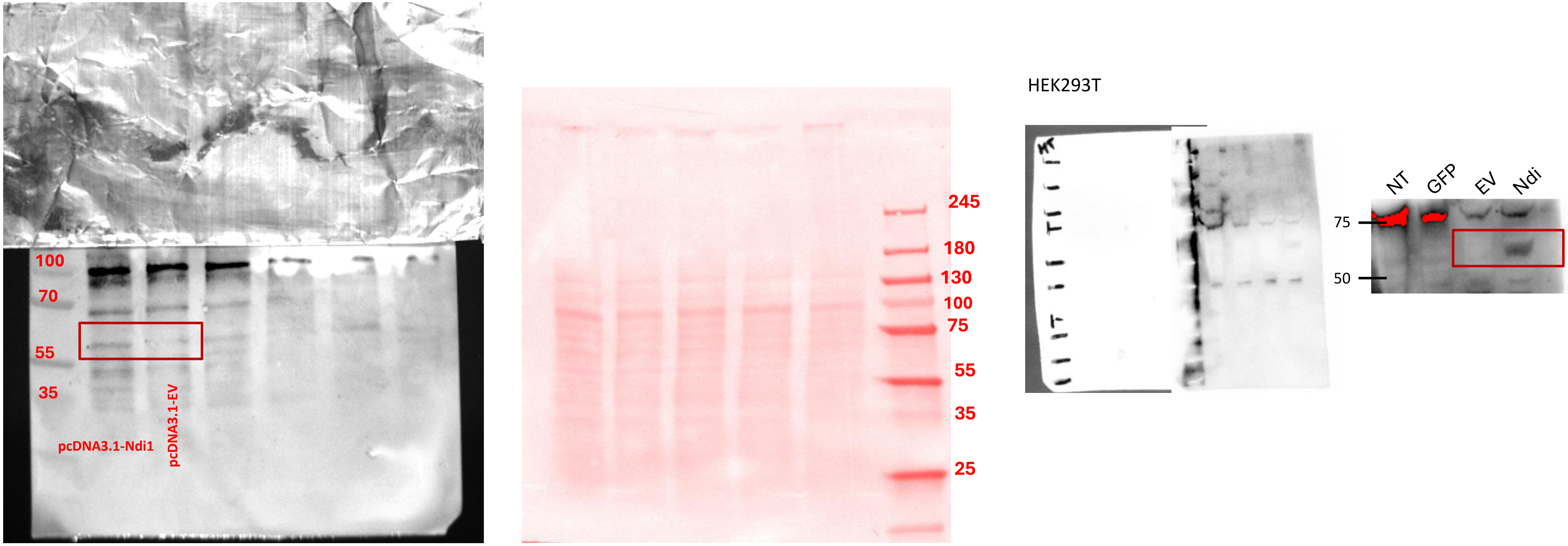

Expected Mw:
S.Cerevisiae Ndi1 + tags = 561aa, 61kDa ca
Hs c-myc = 62 kDa
*ab 2276 recognizes an aspecific protein at 90kDa (unknown) see datasheet
