# Supplementary material for: Bi-directional regulation between NAD/NAMPT and IFN-γ/PD-L1 axes via BRD4/IRF1 and mitochondrial respiration in metastatic cutaneous melanoma
Source: J Exp Clin Cancer Res. 2026 May 14;45:147. doi: 10.1186/s13046-026-03734-2 (PMC13326467; doi:10.1186/s13046-026-03734-2)
Supplement: Supplementary file 5 — Supplementary Material 5. [file 13046_2026_3734_MOESM5_ESM.pdf]

Supplementary Figures and Legends

SUPPLEMENTARY FIGURE 1

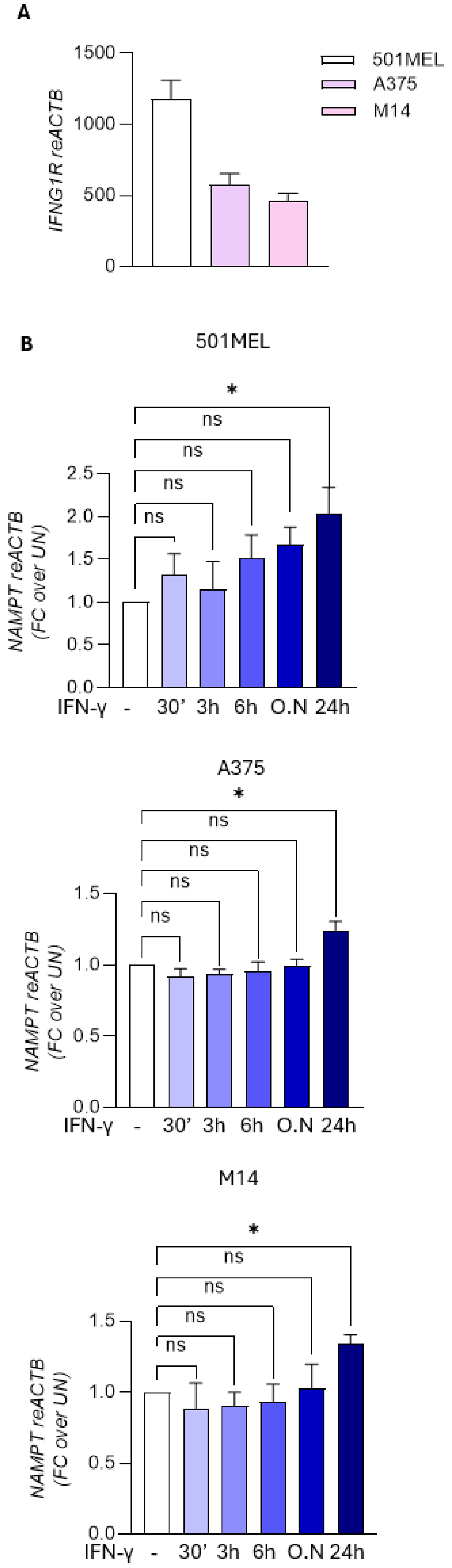

Supplementary Figure 1. **Interferon receptor expression and kinetic of NAMPT induction upon IFN-γ exposure.** (**A**) Histogram representing *IFNG1R* mRNA levels in 501MEL, A375 and M14 cells (n = 6). Data are reported as relative expression values (*IFNG1R/ACTB*). (**B**) Histograms showing relative *NAMPT* mRNA levels at the indicated time points after IFN-γ stimulation (24h): - (untreated, UN), 30 min, 3 h, 6 h, overnight (O.N.) in 501MEL, A375 and M14 cells. Values are expressed as fold change relative to the UN condition. Data are reported as relative expression values (*NAMPT/ACTB*) compared with UN condition. Statistical analysis: one-way ANOVA multiple comparison test vs UN cells; (*$p < 0.05$, **$p < 0.01$, ***$p < 0.001$). Data are presented as mean ± SEM from three independent experiments.

SUPPLEMENTARY FIGURE 2

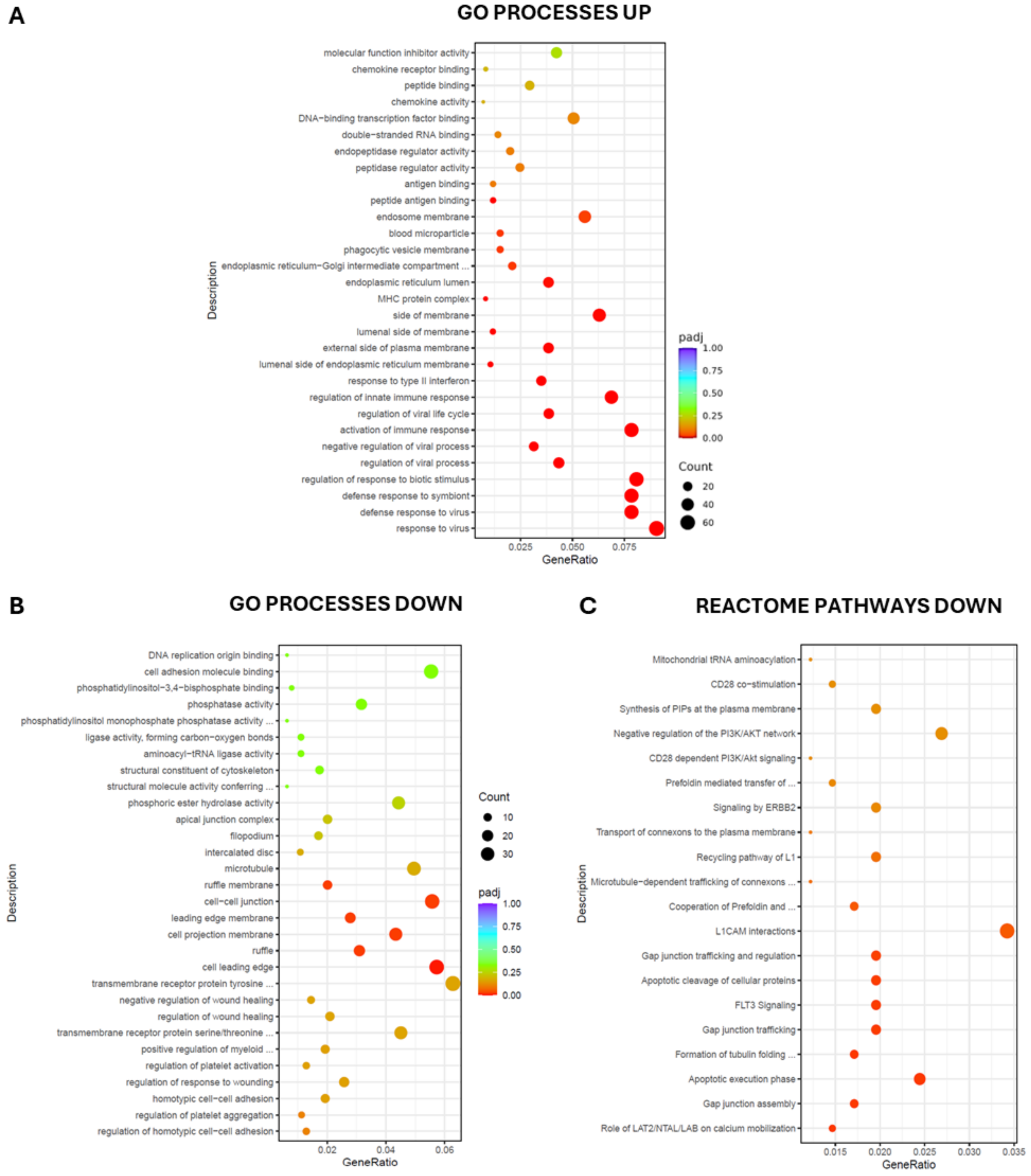

Supplementary Figure 2. IFN-γ treatment analysis. (A) Bubble plots of upregulated genes enriching Gene Ontology (GO) processes in IFN-γ–treated 501MEL cells (IFN-γ 100 ng/ml, 24h). Bubble size and color indicate the number and significance of enriched genes, respectively. (**B-C**) Bubble plots of downregulated genes enriching Gene Ontology (GO, **B**) and Reactome pathways (**C**) in IFN-γ–treated 501MEL cells (100 ng/ml, 24h). Bubble size and color indicate the number and significance of enriched genes, respectively.

SUPPLEMENTARY FIGURE 3

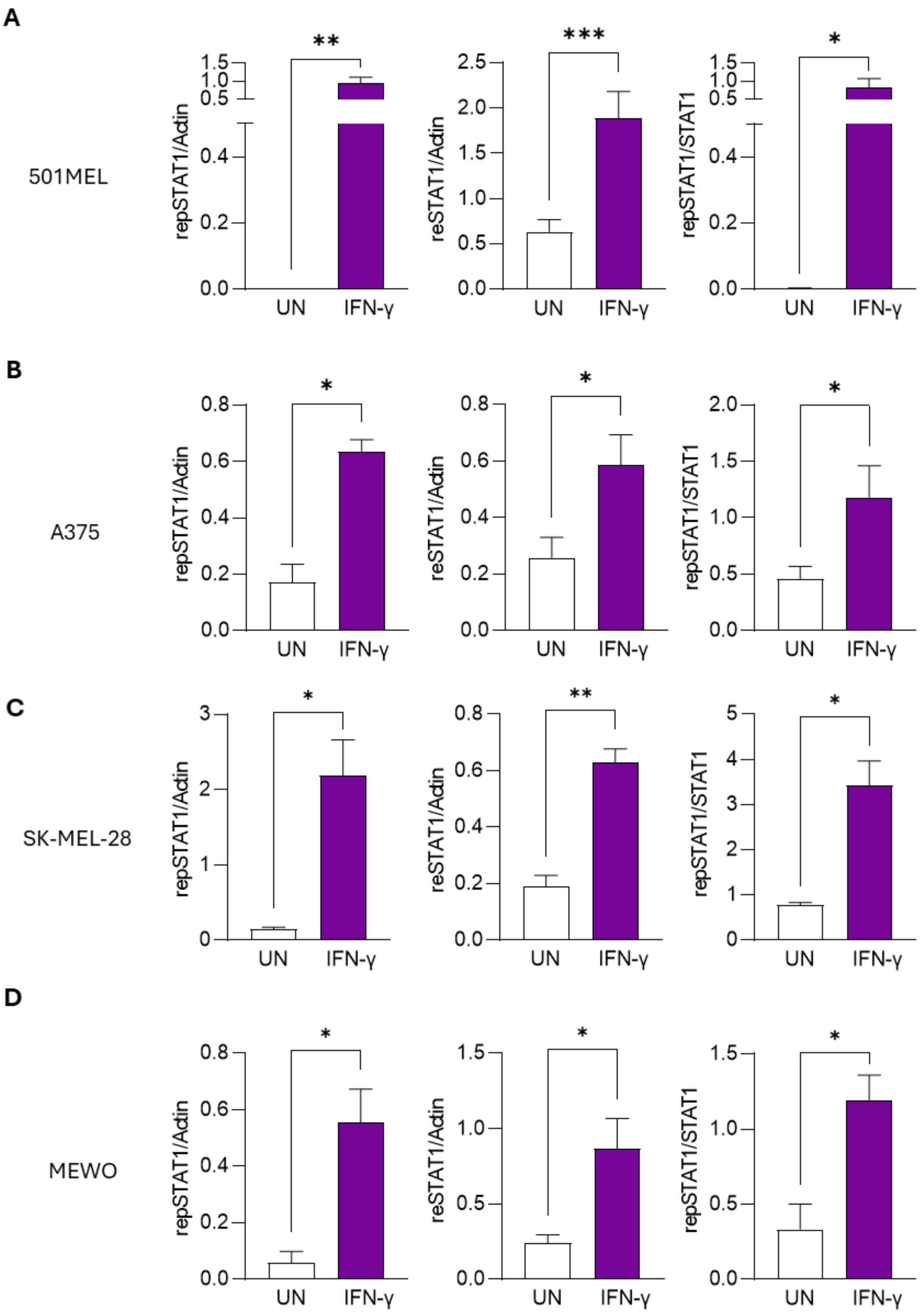

Supplementary Figure 3. IFN-γ signaling activation in human MCM cell lines. Histograms representing pSTAT1 and STAT1 protein levels detected by western blot analysis in 501MEL **(A)**, A375 **(B)**, SK-MEL-28 **(C),** and MEWO **(D)** cells treated with IFN-γ 100 ng/ml for 24h. Data are reported as relative expression values (target gene/Actin). pSTAT1 protein levels are also reported as relative expression values over total STAT1(pSTAT1/STAT1). Statistical analysis: paired t-test (*$p \leq 0.05$, **$p \leq 0.01$, ***$p \leq 0.001$). Bar graphs show mean ± SEM (≥ 4 replicates).

SUPPLEMENTARY FIGURE 4

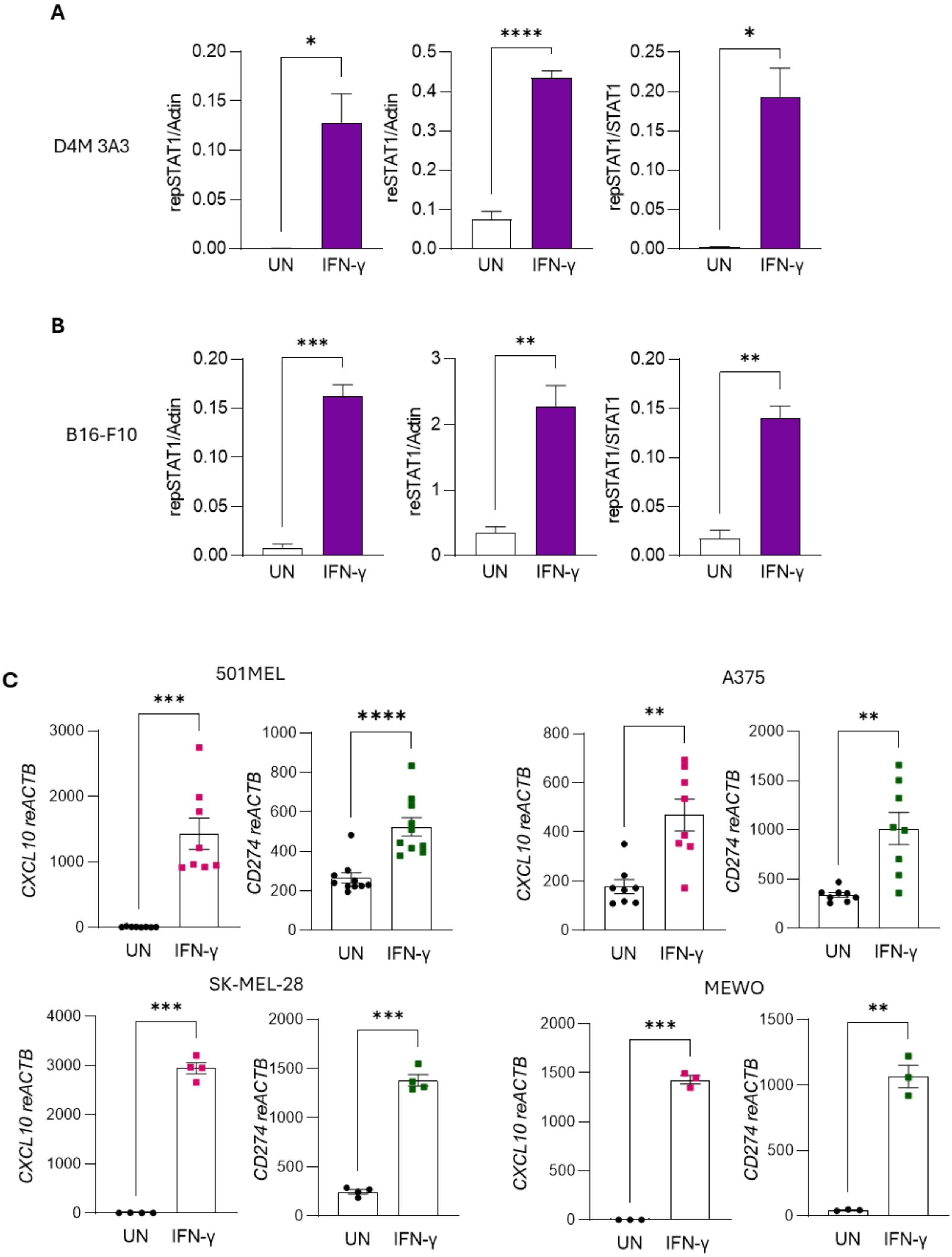

Supplementary Figure 4. IFN-γ signaling activation in MCM cell lines. Histograms representing pSTAT1 and STAT1 protein levels detected by western blot analysis in D4M 3A3 **(A)** and B16-F10 **(B)** cells treated with IFN-γ 100 ng/ml for 24h. Data are reported as relative expression values (target gene/Actin). pSTAT1 protein levels are also reported as relative expression values over total STAT1(pSTAT1/STAT1). Statistical analysis: paired t-test (*p $\leq$ 0.05, **p $\leq$ 0.01, ***p $\leq$ 0.001). Bar graphs show mean ± SEM ($\geq$ 4 replicates). (C) Histograms representing *CXCL10* and *CD274* mRNA in 501MEL (n $\geq$ 8); A375 (n = 8); SM-MEL-28 (n = 4) and MEWO (n = 3) cells after IFN-γ treatment (100 ng/ml, 24 h) vs. untreated (UN). Data are reported as relative expression values (target gene/ACTB). Statistical analysis: paired t-test (*p $\leq$ 0.05, **p $\leq$ 0.01, ***p $\leq$ 0.001). Bar graphs show mean ± SEM.

SUPPLEMENTARY FIGURE 5

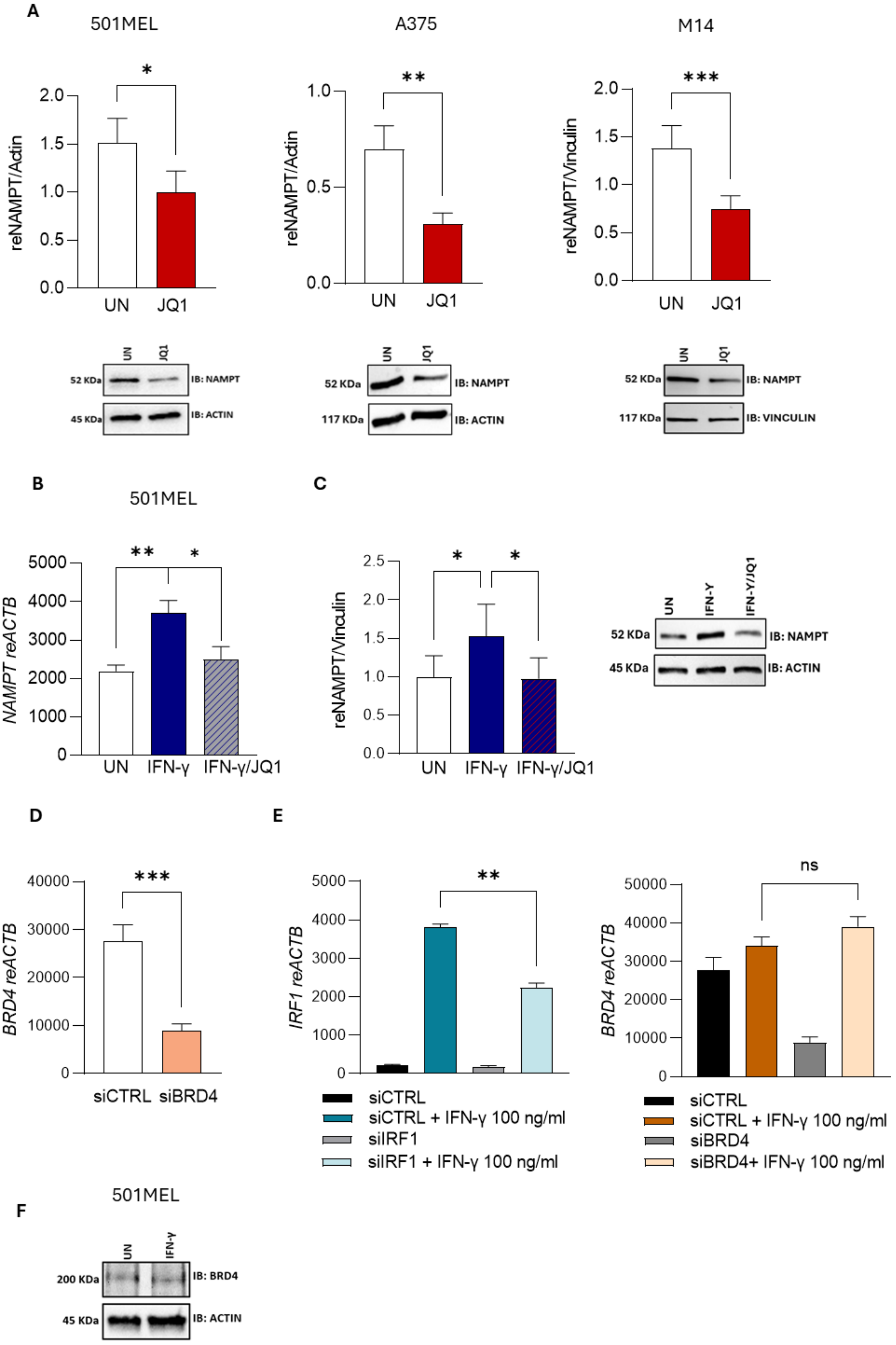

Supplementary Figure 5. NAMPT expression is regulated by BRD4/IRF1/STAT1 modulation. (**A**) NAMPT protein levels in 501MEL, A375 and M14 cells by immunoblot after JQ1 treatment (5 μM; 24 h); data are reported as relative expression values (NAMPT/Actin or NAMPT/Vinculin). Bar graphs show mean ± SEM (≥ 4 replicates). Statistical analysis: paired t-test (*p ≤ 0.05, **p ≤ 0.01, ***p ≤ 0.001). Below graphs the representative blots. (**B**) Histograms showing *NAMPT* mRNA expression in 501MEL cells after treatment with IFN-γ (100 ng/ml) alone or combined with JQ1 (5 μM) for 24 h (n ≥ 4). Bar graph shows mean ± SEM (≥ 4 replicates). Data are reported as relative expression values (NAMPT/ACTB). One-way ANOVA, multiple comparisons over IFN-γ: *p ≤ 0.05, **p ≤ 0.01, ***p ≤ 0.001, ****p ≤ 0.0001. (**C**) NAMPT protein levels in 501MEL cells by immunoblot after treatment as described in B (n ≥ 4). Data are reported as relative expression values (NAMPT/Vinculin). Bar graph shows mean ± SEM (≥ 4 replicates). One-way ANOVA, multiple comparisons over IFN-γ: *p ≤ 0.05, **p ≤ 0.01, ***p ≤ 0.001, ****p ≤ 0.0001. On the right the representative blot. (**D**) Histogram showing *BRD4* mRNA expression in 501MEL cells after transient genetic silencing with siBRD4 (48 h). Data are reported as relative expression values (BRD4/ACTB). Data are represented as mean ± SEM, paired t-test: ***p ≤ 0.001. (**E**) Histograms showing *IRF1* and *BRD4* mRNA expression in 501MEL cells transfected for 48h with siIRF1 or siBRD4, followed by IFN-γ exposure (100 ng/ml) during the last 24 h. Data are reported as relative expression values (target gene/ACTB) n = 4; unpaired t-test: **p ≤ 0.01. Data are represented as mean ± SEM. (**F**) Representative blot of BRD4 protein levels in 501MEL cells after treatment with IFN-γ (100 ng/ml; 24 h); actin served as a loading control.

SUPPLEMENTARY FIGURE 6

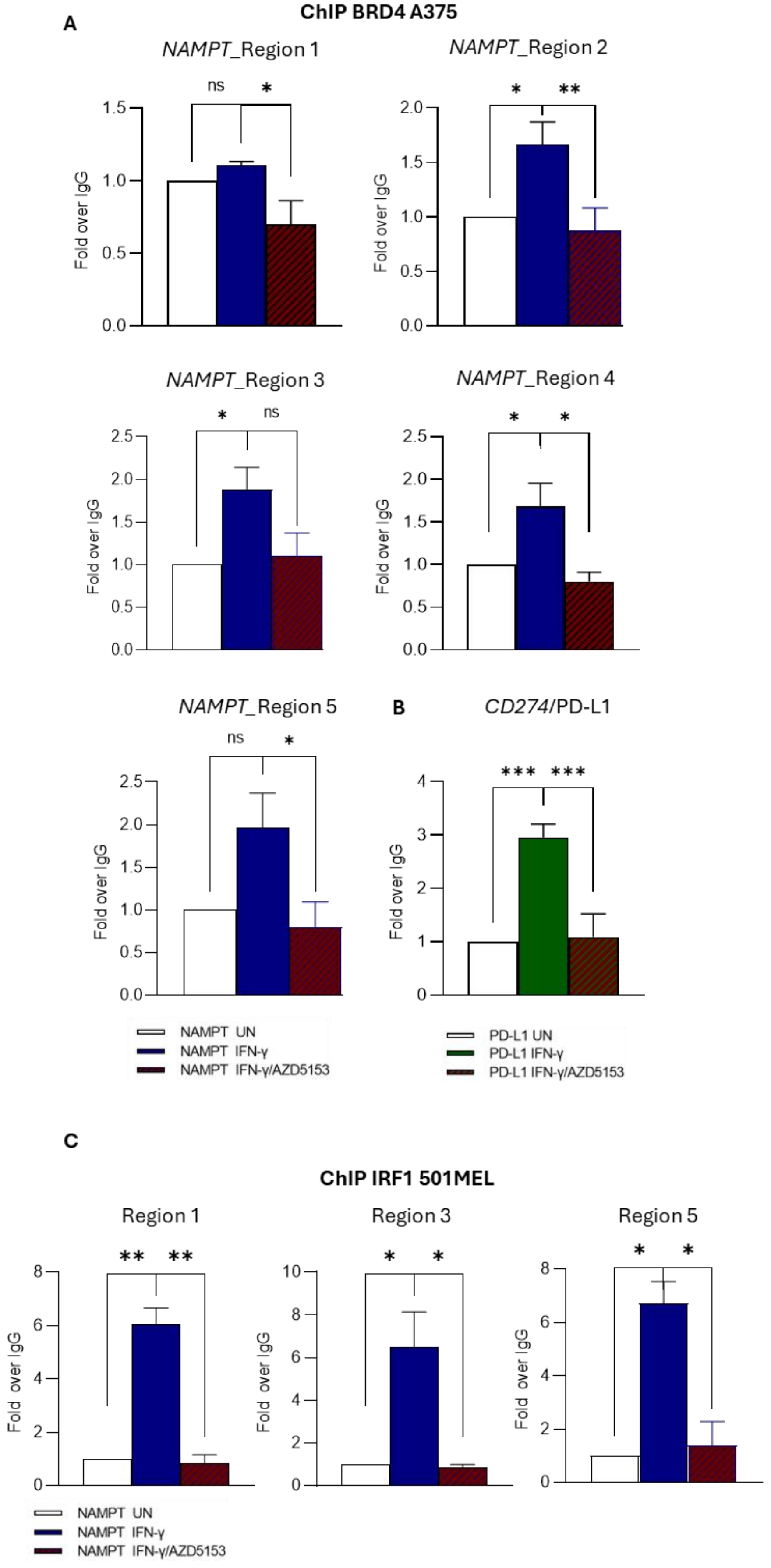

Supplementary Figure 6. *NAMPT* is a direct target gene of BRD4 and IRF1. (A) Graphs showing the results of BRD4 ChIP-qPCR experiment (n=2) in A375 cell line. Graphs represent the effects of IFN-γ (100 ng/ml) treatment alone or in combination with BRD4 inhibitor AZD5153 (100nM) for 24 h on BRD4 binding capacity to *NAMPT* promoter regions, as assessed by ChiP-qPCR. **(B)** ChiP-qPCR data for PD-L1 promoter, used as a positive control for BRD4 binding activity. Treatment conditions are the same as described in A. (**C**) Graphs showing the results of IRF1 ChIP-qPCR experiment (n=3) in 501MEL cell line. Graphs represent the effects of IFN-γ (100 ng/ml) treatment alone or in combination with AZD5153 (100nM) for 24 h on IRF1 occupancy on *NAMPT* promoter regions (region 1, region 3 and 5), as assessed by ChiP-qPCR. In all ChIP–RT-qPCR experiments, results are expressed as fold enrichment over negative control IgG used in ChIP and normalized to untreated cells (UN). Bar graphs show mean ± SEM. One-way ANOVA was used to assess statistical significance, multiple comparisons over the IFN-γ condition (*$p \leq 0.05$, **$p \leq 0.01$, ***$p \leq 0.001$).

SUPPLEMENTARY FIGURE 7

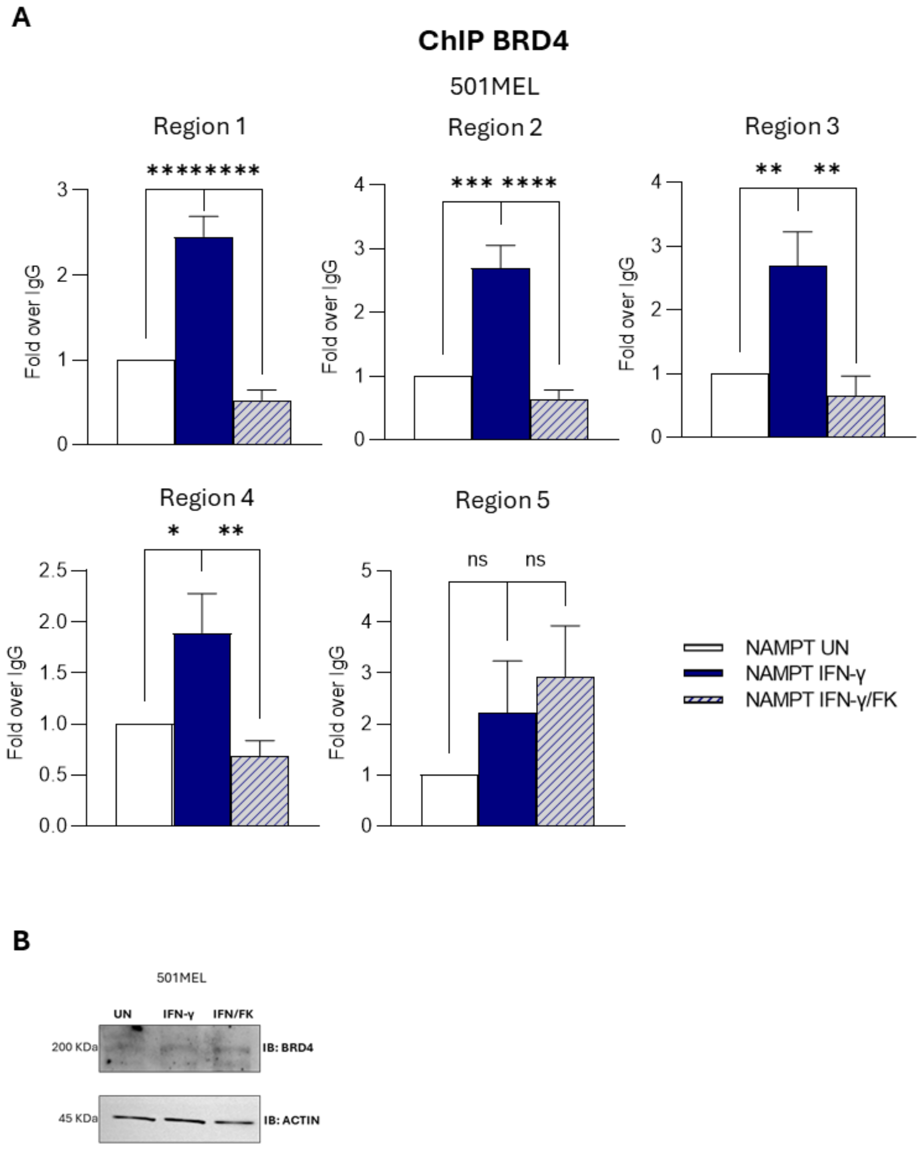

Supplementary Figure 7. BRD4 ChIP in cells treated with IFN-γ and NAMPT inhibitor. (A) Graphs showing the results of BRD4 ChIP-qPCR experiment (n=3) in 501MEL cell line. ChIP–RT-qPCR results are expressed as fold enrichment over negative control IgG used in ChIP and normalized to untreated cells (UN). Graphs represent the effects of IFN-γ (100 ng/ml) treatment for 24 h alone or in combination with FK866 (FK, 25nM) on BRD4 occupancy to *NAMPT* promoter regions, as assessed by ChiP-qPCR). Bar graphs show mean ± SEM. One-way ANOVA was used to assess statistical significance, multiple comparisons over the IFN-γ condition (*$p \leq 0.05$, **$p \leq 0.01$, ***$p \leq 0.001$, ****$p \leq 0.001$).

(B) Representative blot of BRD4 protein levels in 501MEL cells after IFN-γ (100 ng/ml) treatment alone or in combination with FK866 (25nM) for 24 h. Actin served as a loading control.

SUPPLEMENTARY FIGURE 8

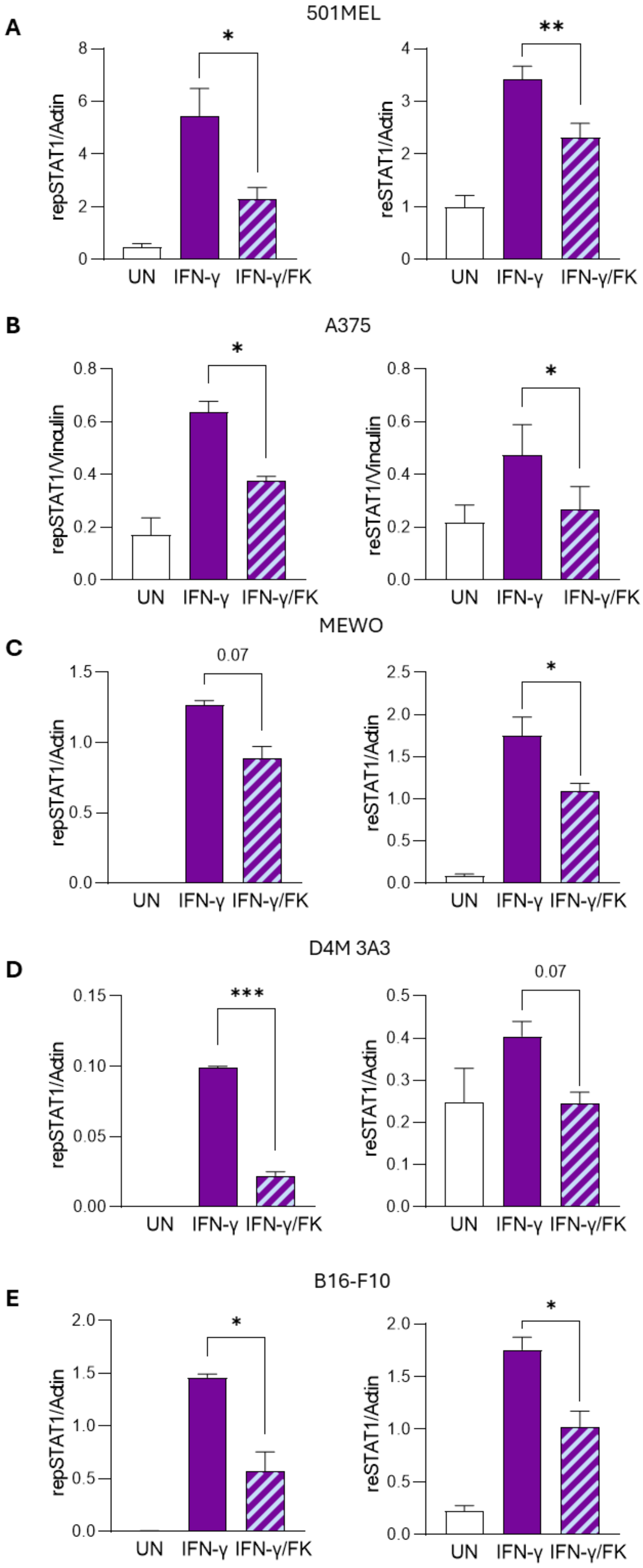

Supplementary Figure 8. Pharmacological NAMPT inhibition impacts on STAT1 activation and expression. Histograms showing cumulative data of western blot analysis of pSTAT1 and STAT1 expression in MCM human and murine cell lines (501MEL, **A**; A375, **B**; MEWO, **C**; D4M 3A3, **D** and B16, **E**) treated with IFN-γ (24 h; 100 ng/ml) alone or in combination with FK866 (FK, 25 nM). Data are reported as relative expression values (target gene/Actin or target gene/Vinculin). Bar graphs show mean ± SEM. Four independent biological replicates were performed for each cell line. Paired T-test was used to calculate statistical significance (*p≤0.05, ** p≤0.01, *** p≤0.001).

SUPPLEMENTARY FIGURE 9

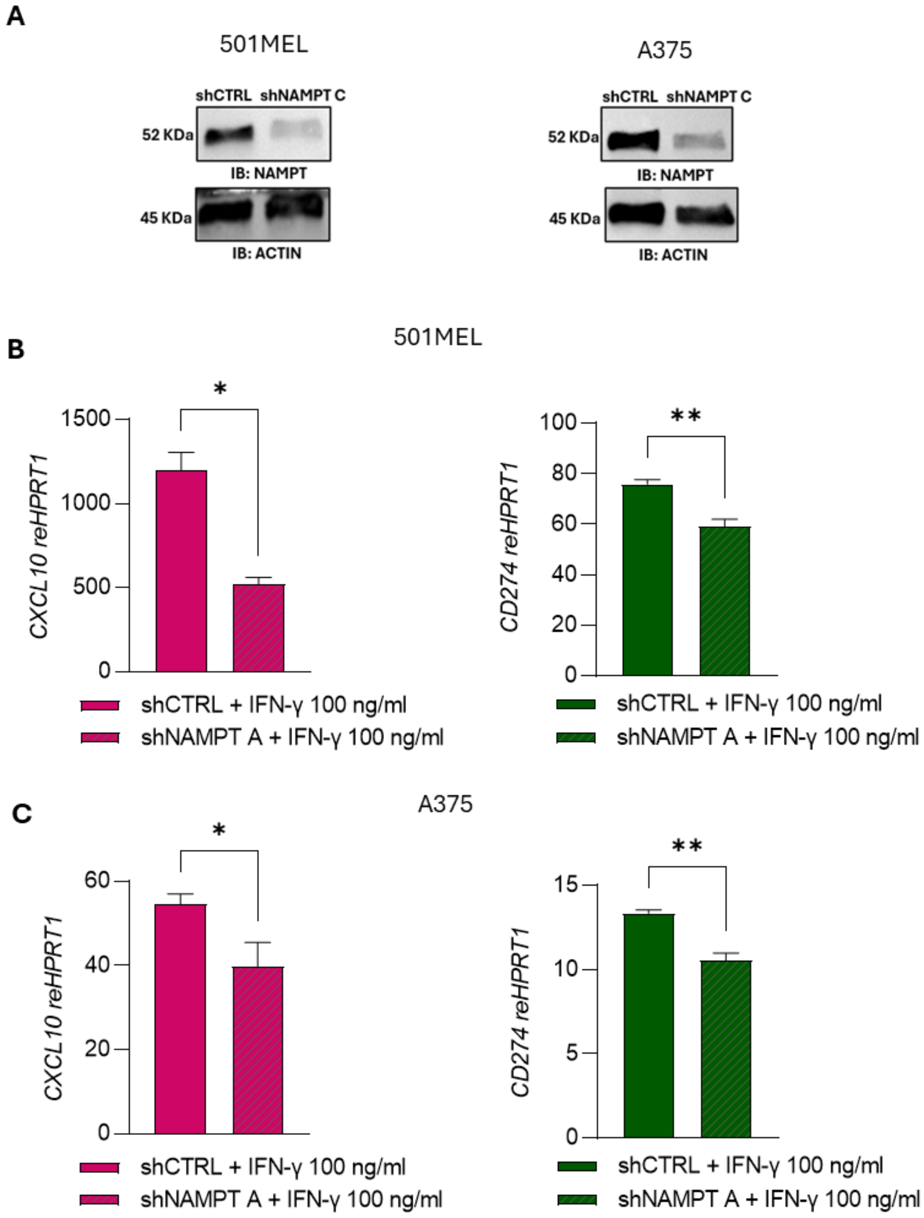

Supplementary Figure 9. Genetic NAMPT silencing and functional effects. (A) Representative blots of NAMPT protein levels in 501MEL and A375 cells after 48 h of transient *NAMPT* genetic silencing with shNAMPT C vs shCTRL; actin served as a loading control. **(B-C)** Histograms reporting *CXCL10* and *CD274* mRNA expression levels in 501MEL (B) and A375 (C) cells after 48

h of transient *NAMPT* genetic silencing with shNAMPT A or shCTRL followed by IFN-γ exposure (100 ng/ml) during the last 24 h. Data are reported as relative expression values (target gene/HPRT1) in both cell lines. Bar graphs show mean ± SEM Data from 4 independent experiments for each line. Paired T-test was used to calculate statistical significance (*p ≤ 0.05, **p ≤ 0.01, ***p ≤ 0.001, ****p ≤ 0.0001).

SUPPLEMENTARY FIGURE 10

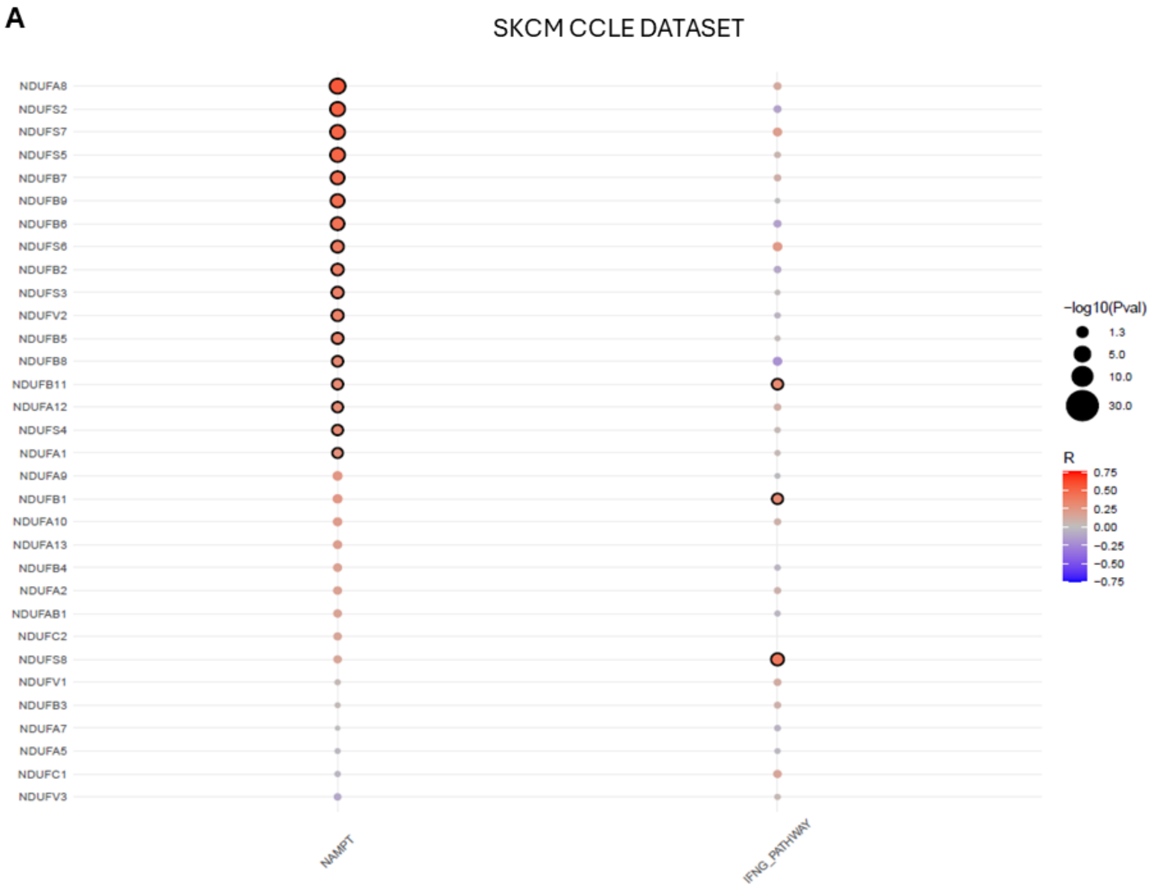

Supplementary Figure 10. MCI genes and NAMPT/IFN correlation. (A) Heatmap-scatter plot correlating NAMPT expression or IFNG pathway with MCI genes in cancer cell line encyclopedia (CCLE) Skin Cutaneous Melanoma SKCM cell lines (n=57). Pearson (R) correlation and –log10(p-value) are shown.

SUPPLEMENTARY FIGURE 11

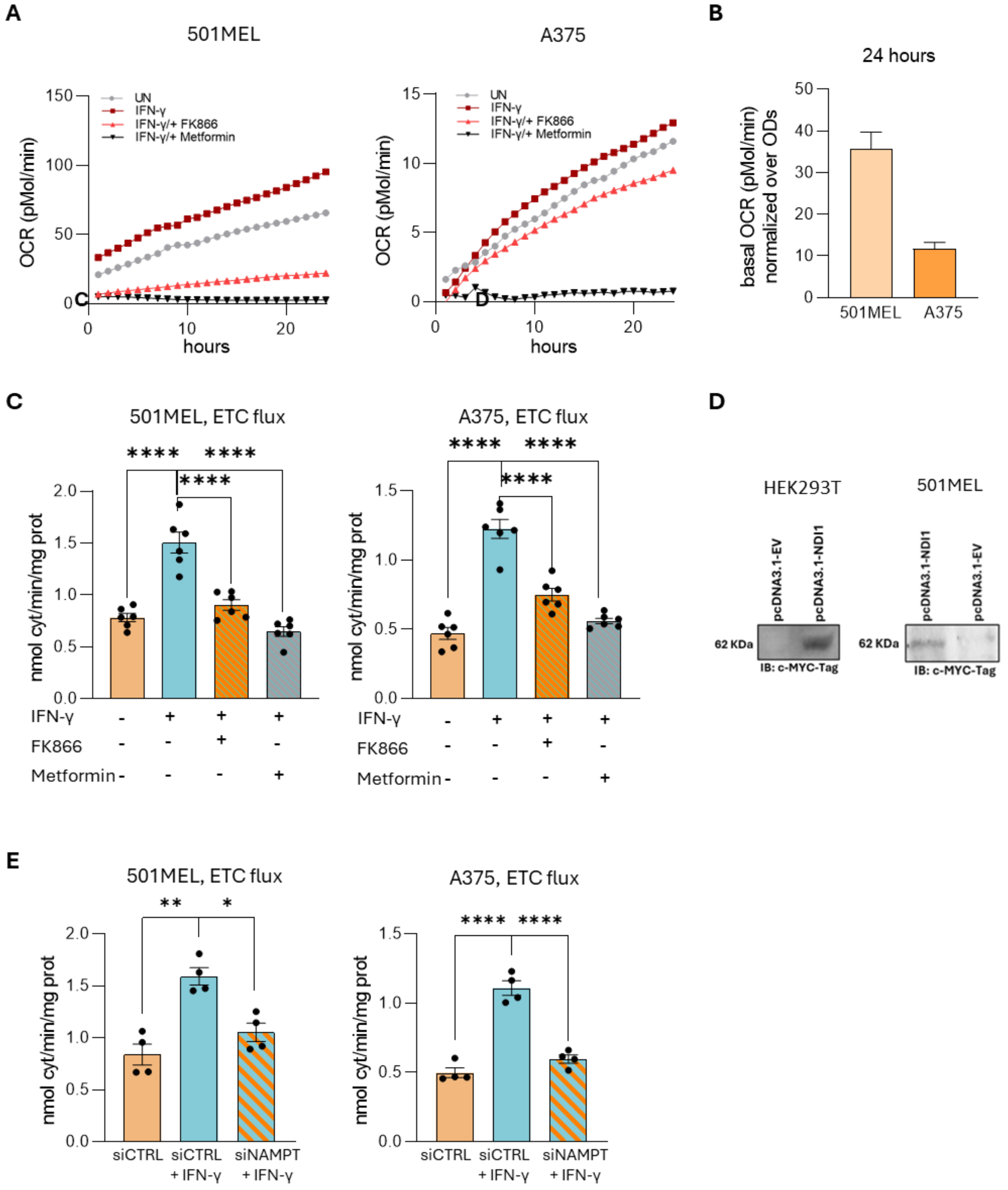

Supplementary Figure 11. Basal OCR, NDI1 expression, ETC flux measurement. (A) Kinetic evolution graphs 0-24 hours of the oxygen consumption rate (OCR, pMol/min) measured using Resipher in 501MEL and A375 in basal/untreated UN condition (grey line), after IFN-γ (100 ng/ml,

red line); IFN-γ/FK866 (25 nM, pink line) and IFN-γ/Metformin (5 mM, black line) treatment. **(B)** Histogram showing basal OCR (pMol/min, 24 h) measured using Resipher system in 501MEL and A375 cells normalized over optical density (ODs) for each sample. Data are represented as mean ± SEM (n=4 replicates). **(C)** Histograms showing the mitochondrial electron flux ETC activity (nmol cyt/min/mg protein) in 501MEL and A375 measured after 24 h of treatment as described previously **(A)** Bar graphs show mean ± SEM. Data from 3 independent experiments for each line, 2 technical replicates. One-way ANOVA, multiple comparisons vs. IFN-γ condition, was used to assess statistical significance (*p $\leq$ 0.05, **p $\leq$ 0.01, ***p $\leq$ 0.001, ****p $\leq$ 0.0001). **(D)** Representative blot confirming the expression of c-MYC-Tag-NDI1 after pcDNA3.1-NDI1 transfection for 48h in 501MEL and HEK293T cells (control) compared to empty vector (pcDNA3.1-EV). **(E)** Histograms report cumulative data of ETC activity in 501MEL and A375 after 48 h of NAMPT genetic silencing adding IFN-γ (100 ng/ml) for the last 24h (siNAMPT/+IFN-γ) compared to cells transfected for 48h with siCTRL (untreated) or siCTRL+IFN-γ adding IFN-γ (100 ng/ml) for the last 24h. OCR data were normalized over optical density (ODs) for each sample. Bar graphs show mean ± SEM Data from 2 independent experiments for each line, 2 technical replicates. One-way ANOVA, multiple comparisons vs. siCTRL/+IFN-γ condition, was used to assess statistical significance (*p $\leq$ 0.05, **p $\leq$ 0.01, ***p $\leq$ 0.001, ****p $\leq$ 0.0001).

SUPPLEMENTARY FIGURE 12

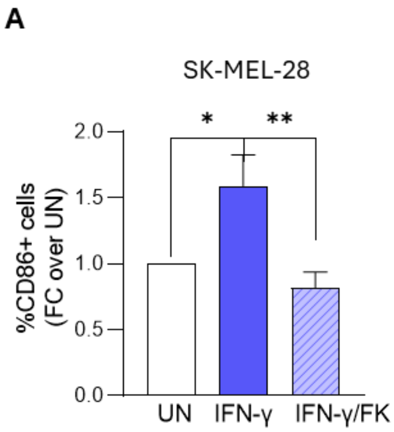

Supplementary Figure 12. CD86 modulation. (A) Histogram shows FACS analysis of the percentage of CD86-positive SK-MEL-28 cells after treatment with IFN-γ (100 ng/ml) alone or combined with FK866 (FK, 25 nM) for 24 h. Data are reported as fold change (FC) compared untreated condition. Bar graphs show mean ± SEM (n=4). One-way ANOVA, multiple comparisons vs. IFN-γ condition, was used to assess statistical significance (*p ≤ 0.05, **p ≤ 0.01).

Supplementary Table

SUPPLEMENTARY TABLE 1

Tumor	R	R_pval	rho	rho_pval
Adrenocortical Carcinoma (ACC)	0.2644	0.0185	0.2366	0.0358
Bladder Urothelial Carcinoma (BLCA)	0.3925	1.78E-16	0.4255	2.27E-19
Breast Invasive Carcinoma (BRCA)	0.3227	6.65E-28	0.3029	1.27E-24
Cervical Squamous Cell Carcinoma and Endocervical Adenocarcinoma (CESC)	0.2635	3.19E-06	0.2958	1.70E-07
Cholangiocarcinoma (CHOL)	0.6292	3.95E-05	0.5773	0.000293
Colon Adenocarcinoma (COAD)	0.2606	8.33E-06	0.2691	4.07E-06
Colon and Rectum Adenocarcinoma (COADREAD)	0.3014	2.11E-09	0.3028	1.77E-09
Diffuse Large B-cell Lymphoma (DLBC)	0.5495	5.24E-05	0.5142	0.000226
Esophageal Carcinoma (ESCA)	0.3185	1.05E-05	0.2989	4.08E-05
Glioblastoma Multiforme (GBM)	0.4218	5.64E-08	0.4416	1.59E-08
Brain Lower Grade Glioma (GBMLGG)	0.6292	4.96E-75	0.6136	1.91E-70
Head and Neck Squamous Cell Carcinoma (HNSC)	0.1623	0.000202	0.1650	0.000159
Kidney Chromophobe (KICH)	0.2010	0.1057	0.2584	0.0365
Pan-Kidney Cohort (KIPAN)	0.2345	1.44E-12	0.2215	2.37E-11
Kidney Renal Clear Cell Carcinoma (KIRC)	0.1592	0.000223	0.1081	0.0125
Kidney Renal Papillary Cell Carcinoma (KIRP)	0.2413	3.29E-05	0.2612	7.07E-06
Brain Lower Grade Glioma (LGG)	0.6053	6.91E-53	0.5104	1.44E-35
Liver Hepatocellular Carcinoma (LIHC)	0.2357	4.44E-06	0.2643	2.41E-07
Lung Adenocarcinoma (LUAD)	0.2581	3.72E-09	0.2421	2.64E-08
Lung Squamous Cell Carcinoma (LUSC)	0.3776	2.02E-18	0.3650	3.08E-17
Mesothelioma (MESO)	0.4055	9.78E-05	0.3870	0.000238
Ovarian Serous Cystadenocarcinoma (OV)	0.2564	6.16E-06	0.1985	0.000520
Pancreatic Adenocarcinoma (PAAD)	0.4335	1.49E-09	0.4191	5.78E-09
Prostate Adenocarcinoma (PRAD)	0.3554	3.03E-16	0.3557	2.87E-16
Rectum Adenocarcinoma (READ)	0.4173	2.86E-05	0.3678	0.000292
Sarcoma (SARC)	0.3800	2.53E-10	0.3895	8.22E-11
Skin Cutaneous Melanoma (SKCM)	0.2521	0.0102	0.2730	0.00541
Stomach Adenocarcinoma (STAD)	0.2683	2.85E-08	0.2732	1.78E-08
Stomach and Esophageal Carcinoma (STES)	0.2719	1.28E-11	0.2805	3.40E-12
Thyroid Carcinoma (THCA)	0.7365	9.08E-87	0.7351	2.82E-86
Thymoma (THYM)	0.6890	3.37E-18	0.6565	~0
Uterine Corpus Endometrial Carcinoma (UCEC)	0.4272	3.35E-09	0.4142	1.10E-08
Uterine Carcinosarcoma (UCS)	0.4443	0.000536	0.4047	0.00180
Uveal Melanoma (UVM)	0.3846	0.000428	0.3306	0.00287

Supplementary Table 1. Correlation between *NAMPT* and *CD274*/PD-L1 across human cencers. Pancancer association analysis between *CD274* and *NAMPT* expression levels derived from TCGA dataset. Correlation was found to be positive and significant across all tumor types, except for Pheochromocytoma, Paraganglioma (PCPG) and Testicular Germ Cell Tumors (TGCT). Correlation analysis was performed with both methods 'Spearman' (rho) and 'Pearson' (R) and was reported the relative statistical significance (pval).
