# Supplementary material for: Bi-directional regulation between NAD/NAMPT and IFN-γ/PD-L1 axes via BRD4/IRF1 and mitochondrial respiration in metastatic cutaneous melanoma
Source: J Exp Clin Cancer Res. 2026 May 14;45:147. doi: 10.1186/s13046-026-03734-2 (PMC13326467; doi:10.1186/s13046-026-03734-2)
Supplement: Supplementary file 6 — Supplementary Material 6. [file 13046_2026_3734_MOESM6_ESM.pdf]

Supplementary Materials and Methods

qRT-PCR list of primers

Commercially available primers [TaqMan Gene Expression Assays (Thermo Fisher Scientific) used were: Hs00237184_m1 (*NAMPT*), Hs04188087_m1 (*BRD4*), Hs00171042_m1 (*CXCL10*), Hs00971965_m1 (*IRF1*), Hs01013996_m1 (*STAT1*), Hs00204257_m1 (*CD274*) and Hs00988304_m1 (*IFNG1R*). Hs99999903_m1 (*ACTB*) and Hs02800695_m1 (*HPRT1*) were used as housekeeping genes.

RNA sequencing

Total RNAs extracted by 501MEL cells were quantified using two different Thermo Fisher instruments Varioskan (spectrophotometry at 260 nm) / Qubit (fluorescent probes). The quality control (QC) was performed by Novogene using Agilent 5400 Fragment Analyzer system (Agilent, Santa Clara, CA, USA). All the 9 samples possessed an integrity value >9.7.

RNA Library Preparation and Sequencing

mRNA was purified using poly-T oligo-attached magnetic beads. Libraries were prepared either as non-strand-specific or strand-specific. For non-strand-specific libraries, first-strand cDNA was synthesized with random hexamer primers followed by second-strand synthesis. For strand-specific libraries, dUTP was used instead of dTTP during second-strand synthesis, allowing directional information retention. Libraries underwent end-repair, A-tailing, adapter ligation, size selection, PCR amplification, and purification. Quality and quantity were assessed using Qubit fluorometry, real-time PCR, and an Agilent Bioanalyzer. Libraries were pooled according to effective concentration and sequenced on an Illumina platform NovaSeq X Plus using the Sequencing-by-Synthesis method to generate paired-end reads (PE150 technology).

Bioinformatic Analysis

Raw FASTQ reads were processed with *fastp* to remove adapter sequences and low-quality reads.

Clean reads were aligned to the reference genome hg38 using *HISAT2* (v2.2.1) (1) with gene model annotation to enable splice-aware mapping. Gene-level read counts were obtained with *featureCounts* (v2.0.6), and expression levels were normalized as FPKM values. Differentially expressed genes (DEGs) were identified using *DESeq2* (v1.42.0) (2) for datasets with biological replicates. Genes with *padj* ≤ 0.05 and $|\log_2 FC| \geq 0$ were considered significantly differentially expressed. Functional enrichment analyses of DEGs were performed using *clusterProfiler* (v4.8.1) (3) for Gene Ontology (GO) and Kyoto Encyclopedia of Genes and Genomes (KEGG) pathway enrichment, with corrected *P* values < 0.05 considered significant.

Antibodies used for western blot

The following antibodies were used for western blot: anti-NAMPT (A300-779A, Bethyl Laboratories, Montgomery, TX, USA), anti-STAT1 (#14994, DIK9Y), anti-p-STAT1 (Y701) (#9167, DA47), anti-BRD4 (#13440) all from Cell Signaling Technology (Danvers, MA, USA); anti-β-Actin (sc-47778) and anti-Vinculin (H-10, sc-25336) both from Santa Cruz Biotechnology (Dallas, TX, USA). Secondary reagents were anti-mouse-IgGk BP-HRP (Santa Cruz Biotechnology #sc-516102) and goat anti-rabbit IgG HRP-conjugated (BioRad Cat.#1706515).

Melanoma Tissue Microarray (TMA)

A total of 129 formalin-fixed and paraffin-embedded (FFPE) samples of MCM, characterized for the BRAF mutational status (56 BRAF-wild-type, 73 BRAF-mutated), were collected from patients diagnosed and treated from 2010 to 2019 at Maria Sklodowska-Curie National Research Institute of Oncology, Warsaw, Poland. High-density tissue microarrays (TMAs) were constructed from archival FFPE left-overs after MCM diagnosis, including three representative 1.0 mm cores from each case. Moreover, in all TMAs, positive and negative controls were incorporated (tonsil, testis, liver, appendix, and normal skin). The use of FFPE sections was conducted in accordance with ethical standards, having received approval from the Local Ethics Committee (16275_bio) under the principles outlined in the Declaration of Helsinki. To ensure the quality and accuracy of subsequent

analyses, 3-micron-thick tissue sections were stained with hematoxylin and eosin (H&E) and carefully reviewed to confirm the histopathological diagnosis and assess tissue integrity.

The NAMPT scoring method involved calculating the product of the percentage of NAMPT- positive cells—ranging from 0% to 100%—and the dominant staining intensity, classified as weak (1), moderate (2), or strong (3). This resulted in an overall H-score ranging from 0 to 300, with scores below 1% positive cells interpreted as negative. Additionally, PD-L1 expression was assessed solely within tumor cells and reported using the Tumor Proportion Score (TPS). The TPS reflects the percentage of tumor cells displaying any level of membranous staining—complete or partial—of positive melanoma cells, regardless of staining intensity.

Preparation of culture supernatants (SN) for eNAMPT quantification by enzyme-linked immunosorbent assay (ELISA) and WB

1,5 x 10^5 MCM cells (A375 and 501MEL) were seeded in 6 well culture plate in complete medium. Cells were treated with IFN-γ (200 ng/ml) for 48 hours. The collected media was centrifuged at 2,800 rcf for 10 minutes to eliminate cell debris and larger cellular-derived particles. Culture SN (diluted 1:2) were used to dose eNAMPT using human NAMPT ELISA kit (Adipogen, Epalinges, Switzerland; AG-45A-0006YEK-KI01). eNAMPT levels (ng/ml) were normalized on optical density (OD) measurements.

For eNAMPT measurement in SN by WB analysis melanoma cells ($5x10^5$) were seeded in T75 flasks in media with 10% FBS. After 48 hours complete media is replaced with media without FBS, and cells were treated with IFN-γ (200 ng/ml) for 48 hours. Spent media was then collected and concentrated 10x using 30 kDa cut off filters (Amicon Ultra centrifugal Filters, Millipore, Milan, Italy) and then analyzed by western blot as described previous.

FACS Analysis

PD-L1 expression was detected using Anti-PD-L1-PE antibody (Anti-human CD274 eBioscience, Thermo Fisher, PE #12-5983-42). CD80 and CD86 expression was detected using Anti-CD80-PE and anti-CD86-FITC antibodies (Anti-human CD80 PE, #305208, anti-CD86 FITC #374204, both from Biolegend, San Diego, CA). HLA-I was detected using Anti-HLA-PE antibody (Anti-human HLA-I, #1p-242-c100, EXBIO, Praha, A.S, CZE). Data were acquired and analyzed using Sysmex-Partec Flow Cytometer (Sismex Europe, Hamburg, Germany). Data were reported as a fold change over untreated condition of the percentage of PD-L1$^+$, CD80$^+$, CD86$^+$ positive cells or of HLA-I Gmean.

Genetic silencing

Cells were seeded in 24-well plates (2.5 µg shRNA/well), 6 well plates (3 µg siRNA/well) and 96 well plates (0.3 5 µg siRNA/well) and transfected with specific shRNAs and siRNAs, using Lipofectamine 2000 (#11668-027 Invitrogen) in Opti-MEM (#11058-021 Thermo Fisher Scientific) medium without antibiotics, following the manufacturer's instructions. After 6 hours, the transfection medium was replaced with complete DMEM containing 10% FBS and antibiotics. Cells were collected after 48 hours for downstream analyses. In a set of experiment 24h after transfection cells were treated for the subsequent 24h with IFN-γ (100 ng/ml). Transfection efficiencies were determined by quantitative real-time PCR (RT-qPCR) and western blot. Each transfection was performed in three technical replicates and in two independent experiments.

Two shRNA sequences targeting NAMPT (shA and shC), along with a control shRNA sequence, are listed:

TYPE	SEQUENCE NAME	SEQUENCE
DNA	shNAMPT A Fw	CCGGCCACCTTATCTTAGAGTTATTCTCGAGAATAACTCTAAGATAAGGTGGTTTTT
DNA	shNAMPT A Rv	AATTAAAAACCACCTTATCTTAGAGTTATTCTCGAGAATAACTCTAAGATAAGGTGG
DNA	shNAMPT C Fw	CCGGGTAACTTAGATGGTCTGGAATCTCGAGATTCCAGACCATCTAAGTTACTTTTT
DNA	shNAMPT C Rv	AATTAAAAAGTAACTTAGATGGTCTGGAATCTCGAGATTCCAGACCATCTAAGTTAC
DNA	hCTR Fw	CCGGCAACAAGATGAAGAGCACCAACTCGAGTTGGTGCTCTTCATCTTGTTGTTTTT
DNA	hCTR Rv	CCGGCAACAAGATGAAGAGCACCAACTCGAGTTGGTGCTCTTCATCTTGTTGTTTTT

Chromatin Immunoprecipitation

ChIP was performed on cultured cells using a standard protocol, starting from cell cultures at approximately 80% confluency for each cell line (~20*10^6/condition) treated with IFN-γ (100 ng/ml) alone and in combination with AZD5153 (100 nM) or FK866 (25 nM) using a standard protocol. Briefly, cells were cross-linked with 1% formaldehyde (Sigma Aldrich 252549-1L) for 15 minutes at room temperature (501MEL) or 10 minutes at room temperature (A375) and quenched 5-10 minutes with 0.125 M PlusOne Glycine (GE Healthcare Life Science 17-1323-01). Nuclei were isolated and lysed, and chromatin was sheared by sonication using Bioruptor twin (diagenode) for 10 cycles (30 s ON/30 s OFF for 501MEL) or 8 cycles for A375 to obtain DNA fragments of approximately 300–500 bp, as verified by agarose gel electrophoresis. Chromatin corresponding to 300 µg of total DNA was used for each immunoprecipitation. Samples were pre-cleared with Pierce™ Protein A Magnetic Beads (Thermo Fisher Scientific #88845), then incubated overnight at 4 °C with either anti-BRD4 antibody (1:50, #13440 Cell Signaling Technology), anti-IRF1 (1:50; #8478 Cell Signaling Technology) or normal rabbit IgG (Cell Signaling Technology #2729) as a control. Immunocomplexes were captured using Dynabeads™ Protein A (Thermo Fisher Scientific #10001D), followed by sequential washes (low salt, high salt, LiCl, and TE buffers). Cross-linking was reversed overnight at 65 °C, and samples were treated with proteinase K (Sigma Aldrich, #P2308-5M**)** (100 µg/mL) for 1 h at 37 °C. DNA was purified by phenol–chloroform extraction using Trizol (Invitrogen, Thermo Fisher) and ethanol precipitation. Specific primers for qRT-PCR to amplify NAMPT promoter regions and PD-L1 were:

NAMPT promoter regions:

R1: FW_1: ACTTGTGCAGGCCTCACTCC; RW_1: TGCTGGGATTACAGACGTGAGCC;
R2: FW_2: GCTCACGTCTGTAATCCCAGCAC; RW_2: GGAGGCATGGCTGAGACTTCT;
R3: FW_3: CCCCGGTAAAACACAGGGAA; RW_3: TCATGCGTGGTTGCGTTTTT ;
R4: FW_4: AAAAACGCAACCACGCATGA; RW_5: AGCCTCCTACTGCCCATCTT;
R5: FW_5: GACGGGAAGATGGGCAGTAG; RW_5: TTAAGTCACTGCTCGGTCGG.

PD-L1 promoter:

FW_: 5′-AAGCCATATGGGTCTGCTC-3′; RW_: 5′-TTATCAGAAAGGCGTCCCCC-3′.

NDI1 overexpression and treatment

Experimental design

Cells were seeded in 96-well plates at approximately 60–70% confluence and transfected with 0.3 μg/well (96-well plate) of the NDI1 expression vector using Lipofectamine 2000 (Thermo Fisher Scientific, #11668019) following the manufacturer's protocol. After 6 h, the transfection medium was replaced with fresh complete growth medium. 24 hours post-transfection, cells were treated with IFN-γ (100 ng/ml) in combination with FK866 (FK, 25 nM), and IFN-γ with metformin (Met, 5 mM) for 24 hours (48 hours total), cells transfected with empty vector were used as control.

Oxygen consumption rate (OCR) was monitored in real time using the Resipher system (Lucid Scientific) as described in the main text.

Supplementary References

1. Mortazavi A, Williams BA, McCue K, Schaeffer L, Wold B. Mapping and quantifying mammalian transcriptomes by RNA-Seq. Nat Methods. 2008; 5(7):621–8. Available from: https://pubmed.ncbi.nlm.nih.gov/18516045/
2. Anders S, Huber W. Differential expression analysis for sequence count data. Genome Biol 2010 Oct 27; 11(10). Available from: https://pubmed.ncbi.nlm.nih.gov/20979621/
3. Yu G, Wang LG, Han Y, He QY. clusterProfiler: an R package for comparing biological themes among gene clusters. OMICS. 2012 May 1; 16(5):284–7. Available from: https://pubmed.ncbi.nlm.nih.gov/22455463/
